# A General-Purpose Model Adsorption Isotherm and Whole Isotherm Surface Area Measurement Method

**DOI:** 10.1021/acsomega.5c13463

**Published:** 2026-03-31

**Authors:** Thomas A. Manz, W. Nicholas Delgass

**Affiliations:** † School of Chemical Engineering, Purdue University, West Lafayette, Indiana 47907, United States

## Abstract

A popular way to measure the surface areas of porous solids involves fitting a small section of the experimentally measured liquid nitrogen, liquid argon, or moisture adsorption isotherm to the Brunauer–Emmett–Teller (BET) model. A small section is used, because the BET isotherm’s shape differs dramatically from the experimental isotherm’s shape. Several studies proposed consistency criteria to automate choosing the small isotherm portion fitted during BET analysis. In this article, we follow a different approach that constructs a general-purpose model adsorption isotherm with enough flexibility to fit the experimental adsorption isotherm across its entire pressure range. This enables the whole adsorption isotherm to be used to extract surface areas of porous materials. Our MD model isotherm is formulated to apply to fluids both below and above the critical temperature and critical pressure. Mathematical derivations revealed this isotherm is non-negative, monotonically increasing with x_A_, and has correct limiting behaviors. We explain relationships between the MD model isotherm and many model isotherms described in prior literature. When using a single sitegroup, the MD model has five fitted parameters. We introduce a bootstrapping algorithm that computes 95% confidence intervals on the optimized model parameters and whole isotherm surface area. We present a detailed derivation of this new model isotherm and illustrate its utility with diverse examples: (a) liquid N_2_ adsorption onto several porous nickel alloy sponges and metal–organic frameworks (MOFs), (b) diverse examples of gas adsorption in MOFs, (c) moisture adsorption onto various hydrophilic and hydrophobic materials, (d) adsorption of several proteins onto hydrophobic interaction chromatography resins, (e) CO_2_ adsorption on Zeolite 13X, (f) butylamine adsorption from methanol–water solution onto sponge nickel catalysts, (g) aqueous red dye adsorption onto beechwood, (h) step-like layer-by-layer adsorption of methane onto a MgO surface at 87 K. These examples spanned: (i) each of the six IUPAC physisorption isotherm types, (ii) positive cooperative, negative cooperative, and noncooperative adsorption, (iii) single-layer and multilayer adsorption, (iv) adsorption with and without capillary condensation, and (v) adsorption modeled by a single sitegroup and by multiple sitegroups. Results showed the MD isotherm is a good general-purpose model for physical adsorption under diverse conditions on diverse materials.

## Graphical abstract

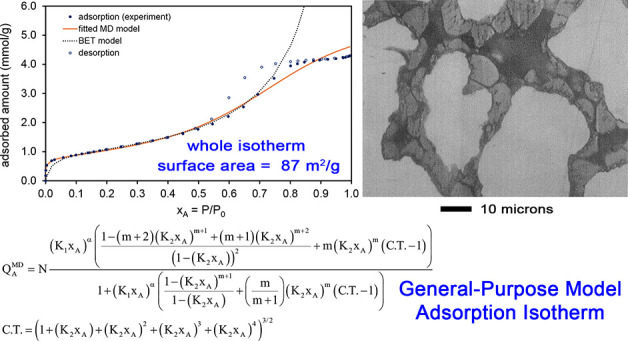

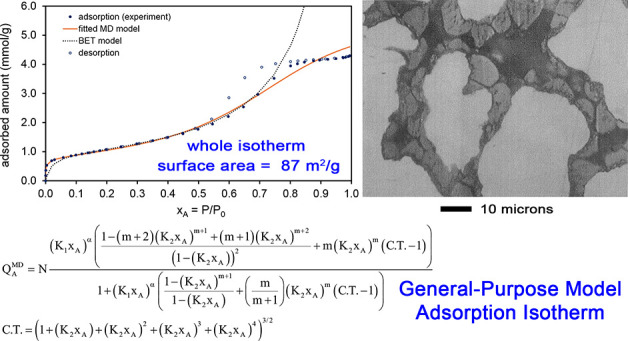

## 1 Introduction

Brunauer et al. introduced a classification of physisorption isotherms into five basic types.^1^ These are included in the 2015 International Union of Pure and Applied Chemistry (IUPAC) classification of physisorption isotherms that also adds a sixth type as shown in Figure 1.^2^ Briefly, Type 1 isotherms are exhibited by single-layer adsorption and by multilayer adsorption into small nanopores that fill at low P_A_/P_A_^sat^ values. Here, P_A_ is the (partial or total) pressure of species A, and P_A_^sat^ (also called P_0_) is the saturation vapor pressure of species A at the adsorption temperature. Type 2 isotherms are characteristic of multilayer adsorption onto nonporous or macroporous materials that have more attractive adsorbate–adsorbent interactions than adsorbate–adsorbate interactions. In these isotherms, the point marked B indicates near monolayer coverage. Type 3 isotherms are characteristic of multilayer adsorption onto nonporous or macroporous materials that have much weaker adsorbate–adsorbent interactions than adsorbate–adsorbate interactions. Type 4 isotherms are characteristic of multilayer adsorption onto mesoporous materials that have more attractive adsorbate–adsorbent interactions than adsorbate–adsorbate interactions. Due to capillary condensation, these micropores saturate at P_A_/P_A_^sat^ values significantly less that one. In Type 4a isotherms, there is hysteresis between the adsorption and desorption branches, which occurs in mesopores wider than about 4 nm.^2^ In Type 4b isotherms, there is no hysteresis between the adsorption and desorption branches. Type 5 isotherms are characteristic of multilayer adsorption onto mesoporous materials that have weaker adsorbate–adsorbent interactions than adsorbate–adsorbate interactions. “For instance, Type V isotherms are observed for water adsorption on hydrophobic microporous and mesoporous adsorbents.”^2^ Finally, Type 6 isotherms are observed in situations where the adsorption isotherm exhibits a stairstep structure for which the adsorbed amount (aka ‘uptake’) increases in multiple distinct steps as P_A_/P_A_^sat^ increases.

**Figure 1:**
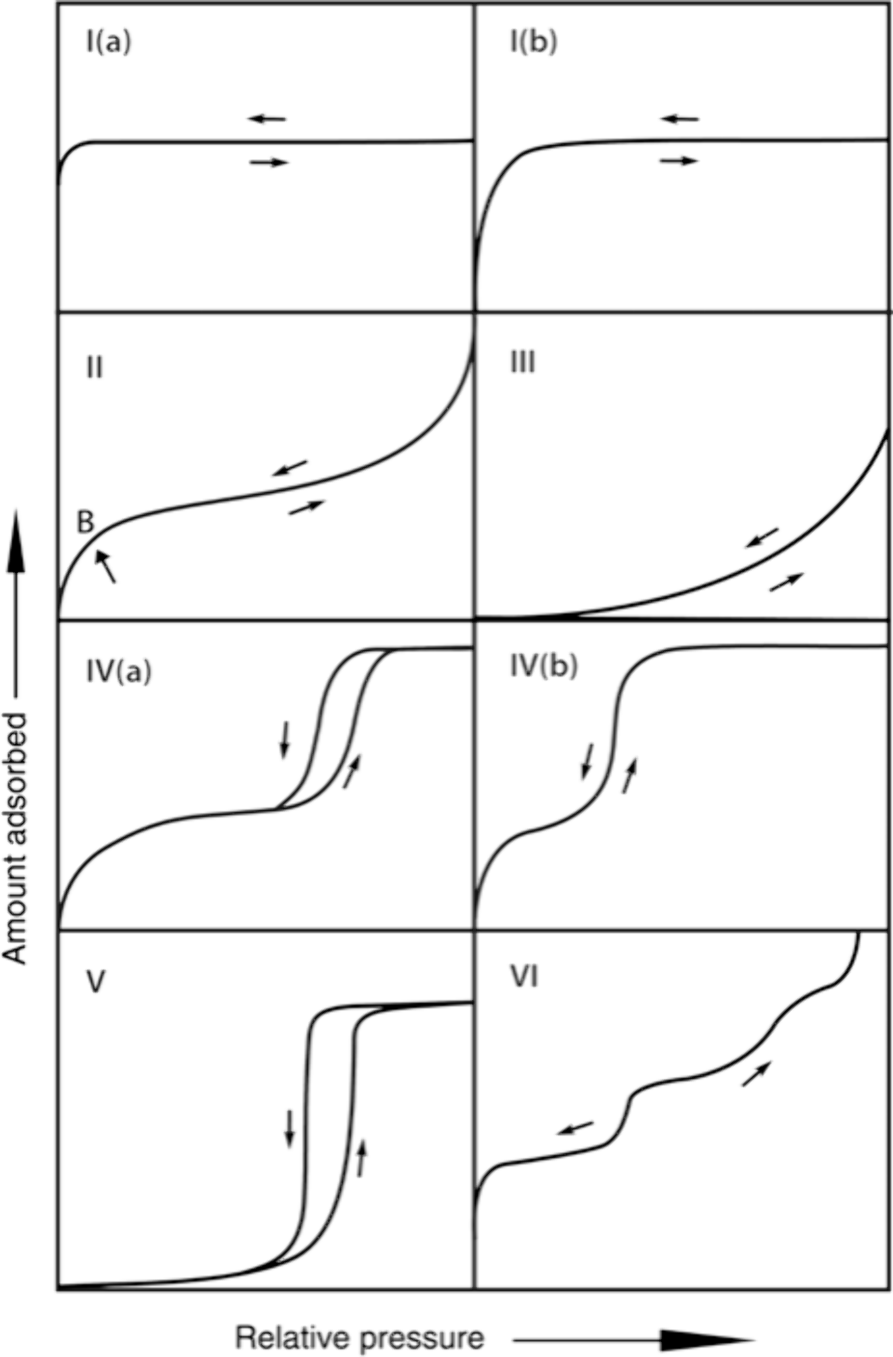
2015 IUPAC classification of physisorption isotherm types. Reproduced with permission from Thommes et al. *Pure Appl. Chem.* **2015**, *87*, 1051–1069. Copyright 2015 International Union of Pure and Applied Chemistry (IUPAC) and De Gruyter.

As discussed in several isotherm comparison and review articles, a large number of adsorption model isotherms were introduced that contain 1 (i.e., Henry’s constant), 2 (e.g., Langmuir, Freundlich, etc.), 3, 4, 5, or more adjustable fitted parameters.^3–13^ Several authors also proposed statistical thermodynamic models of adsorption.^14–23^ These models can be applied to adsorption of molecules from either a gas phase or a liquid phase onto a (porous or nonporous) solid surface.^12,20,24,25^ Following are some key questions to consider when evaluating each these models. How physically interpretable are the model parameters involved? How flexible is the model isotherm for describing various adsorption isotherms? Typically, there is a trade-off between having fewer model parameters to keep the model simpler and having more model parameters to enhance its flexibility for fitting various isotherm shapes.

In recent years, there has been increasing interest in developing unification model isotherms that can describe all six physisorption isotherm types. Ng and co-workers developed a universal isotherm model that combines (a) the Homotattic Patch Approximation, (b) a Langmuir model rewritten in terms of adsorption site energies, and (c) the probability distribution of site energies.^26–28^ They used this to model all six IUPAC physisorption isotherm types.^26–28^ Kong and Adidharma proposed an adsorption theory based on a canonical partition function model applied to attractive regions.^16^ They used this to model all six IUPAC physisorption isotherm types, including both with and without hysteresis for Types IV and V.^16^ Shimizu and co-workers developed the ABC isotherm based on statistical-thermodynamic-fluctuation theory and applied it to study various sorption types and material surface areas.^18–20,29–31^

In this article, we introduce a new general-purpose model adsorption isotherm that is conceptually simple, theoretically well-motivated, flexible enough to reproduce common adsorption isotherm shapes for all six isotherm types, has five model parameters (per surface patch type) that are easy to interpret physically, and reduces to at least eight different commonly used model adsorption isotherms in various limiting cases. Section 2 presents the theory behind this new general-purpose model adsorption isotherm. Section 3 presents the results of fitting this model isotherm to a diverse set of experimentally measured adsorption isotherms. Some of these adsorption isotherms were experimentally measured by us and some were taken from the published literature. Section 4 summarizes our conclusions.

This new general-purpose model isotherm enables the surface areas of porous solids to be measured by fitting the adsorption isotherm over the whole pressure range:

1 $$0<P_{A}/P_{A}^{\mathrm{sat}}<1$$

A previous study by Osterrieth et al. showed the surface area measured using the BET method depends on the pressure segment (i.e., starting and ending pressure) used in the fitting process.^32^ This led to the undesirable situation in which different research groups extracted different BET surface area values by choosing different pressure segments, even though they analyzed exactly the same underlying data set.^32^ Our whole isotherm surface area measurement method avoids this problem by fitting the entire adsorption isotherm to our general-purpose model isotherm.

## 2 Theory

### 2.1 Our General-Purpose Model Adsorption Isotherm

Our unified adsorption isotherm is based on the following model. For adsorption from the gas phase, x_A_ = P_A_/P_A_^sat^, and in this case

2 $$0\leq x_{A}\leq 1$$

For a liquid phase in which A is a solute dissolved in some solvent, x_A_ could be the mass fraction or the mole fraction of species A in the solution or the concentration of species A in the solution. In this article, we consider the case in which A is the only species from the gas phase that adsorbs on the adsorbent material, or in which A is the only liquid-phase solute molecule that displaces solvent molecules to adsorb on the adsorbent material. Other than A and the liquid-phase solvent, the gas or liquid phase may (but is not required to) contain various nonadsorbing chemical species.

For a gas-phase adsorption, let S denote an empty surface adsorption ‘region’. For a liquid-phase adsorption, let S denote a solvent-covered surface adsorption ‘region’. Let AS denote a molecule of species A that has been nondissociatively adsorbed onto the surface. Fractional coverages are denoted by [] brackets. For example, [S] denotes the fractional coverage of free regions, and [AS] denotes the fractional coverage of AS regions.

Here, the term ‘region’ represents a surface piece that can adsorb one molecule of chemical species A in the first adsorption step. The focus here is on a surface unit that adsorbs one molecule of species A rather than focusing on individual atoms of the surface. For example, on a pure metal crystal face, the focus here is not on the individual atoms of the crystal face but rather on a piece of the surface that is capable of adsorbing one A molecule in the first adsorption step. Because a molecule of benzene typically has a larger cross-sectional area than a molecule of N_2_, the ‘S’ for benzene (i.e., benzene + S → benzene–S) accommodates its cross-sectional area and is larger than the ‘S’ for N_2_ which accommodates its cross-sectional area (i.e., N_2_ + S → N_2_–S). This notation is required to account for the different cross-sectional areas of different adsorbing molecules. An adsorption ‘region’ can also be called an adsorption ‘site’; however, it is important to remember these are defined in terms of units of surface that adsorb one A molecule in the first adsorption step, rather than being defined as individual surface atoms.

Let A_j_S denote a surface region onto which j molecules of species A have been adsorbed. To keep this analysis as general as possible, we do not *a priori* restrict the geometric configuration of A_j_S.

Let K_1_ denote the adsorption equilibrium constant for the first adsorption step:

3 $$A+S\overset{K_{1}}{↔}\mathrm{AS}$$

4 $$\left[\mathrm{AS}\right]={\left(K_{1}x_{A}\right)}^{\alpha }\left[S\right]$$

In eqn [Disp-formula eq4], the exponent α is reminiscent of the Freundlich^33,34^ and Sips^35,36^ (aka Langmuir–Freundlich) isotherms.

The range 0 < α < 1 corresponds to the situation in which the first adsorbate molecules adsorb on the most energetically favorable sites, while later ones adsorb on somewhat less favorable sites. This nonisotropy can be caused either by intrinsic properties of the sites themselves (i.e., energy heterogeneity of surface sites) or by lateral repulsive interactions between adsorbate molecules (even if all surface sites are equivalent).

Let K_2_ denote the effective adsorption equilibrium constant for the second adsorption step:

5 $$A+\mathrm{AS}\overset{K_{2}}{↔}A_{2}S$$

6 $$\left[A_{2}S\right]=K_{2}x_{A}\left[\mathrm{AS}\right]={\left(K_{1}x_{A}\right)}^{\alpha }K_{2}x_{A}\left[S\right]$$

To keep the mathematical model simple, we approximate the effective adsorption equilibrium constant for the j^th^ adsorption step except for the last step (i.e., for 1 < j < (1 + m)) as the same as for the second step:

7 $$A+A_{j-1}S\overset{K_{2}}{↔}A_{j}S$$

8 $$\left[A_{j}S\right]=K_{2}x_{A}\left[A_{j-1}S\right]={\left(K_{1}x_{A}\right)}^{\alpha }{\left(K_{2}x_{A}\right)}^{j-1}\left[S\right]$$

This approximation uses one first-step (i.e., K_1_) equilibrium constant and one subsequent-step (i.e., K_2_) equilibrium constant. With respect to a multistep binding process, Klotz presented an elementary adsorption model in which each step j of the process has its own distinct value of the adsorption equilibrium constant, K_j_.^37,38^ Of course, that model is more general and universal than the model we use here, which presumes that the second and subsequent adsorption steps have the same equilibrium constant K_2_.

The range α > 1 exhibits positive cooperativity. Examples of this case include attractive lateral interactions between adsorbate molecules in the first layer on a solid surface. Choosing α = 1 and K_2_ > K_1_ also exhibits positive cooperativity. This corresponds to a situation in which the second adsorption step has a greater affinity for adsorption than the first adsorption step. Examples of this case include: (i) multilayer adsorption in which adsorbate–adsorbate interactions are more attractive than adsorbate–surface interaction and (ii) cooperative binding of O_2_ to hemoglobin.

In materials having micropores or mesopores, capillary condensation occurs in the limit K_2_x_A_ → 1. When this occurs, the micropores and mesopores fill with liquid-phase A. This choice of imposing phase condensation in the limit K_2_x_A_ ≈ 1 instead of in the limit x_A_ ≈ 1 is made so that the isotherm can be applied even for supercritical fluids and solution-phase adsorption. Because supercritical fluids do not have a saturation vapor pressure, instead of defining x_A_ as P_A_/P_A_^sat^, one could instead use P_A_ itself in place of x_A_ (i.e., x_A_ = P_A_). In liquid-phase solutions, x_A_ could be set equal to the concentration (C_A_) or mass fraction (or mole fraction) of species A. The product K_2_x_A_ is dimensionless, because K_2_ has the same measurement units as 1/x_A_.

Let m denote the effective number of adsorption steps beyond the first layer; in other words, m is the effective number of layers that are governed by K_2_. This definition of m is required to match the positivity constraint:

9 m≥0

To model phase condensation (i.e., site saturation as K_2_x_A_ → 1), adsorption for the last step is modified as follows:

10 $$\left[A_{\left(m+1\right)}S\right]=\left(1+\left(\frac{m}{m+1}\right)\left(\mathrm{C.T.}-1\right)\right){\left(K_{1}x_{A}\right)}^{\alpha }{\left(K_{2}x_{A}\right)}^{m}\left[S\right]$$

where the ‘condensation term’ (C.T.) denotes the extra adsorption due to condensation effects. When m = 0, eqn [Disp-formula eq10] becomes equal to the monolayer adsorption governed by eqn [Disp-formula eq4], and in this case the condensation term in eqn [Disp-formula eq10] is multiplied by zero. This is appropriate, because monolayer (i.e., m = 0) adsorption should reduce to the Langmuir or Sips isotherms that have no phase condensation. When m > 0, eqn [Disp-formula eq10] enhances adsorption for the last step to model phase condensation.

The condensation term (C.T.) should be approximately equal to one when K_2_x_A_ ≈ 0 and much larger than one when K_2_x_A_ ≥ 1. Please see Supporting Information Section S4 for a derivation of the following form:

11 $$\mathrm{C.T.}={\left(1+\left(K_{2}x_{A}\right)+{\left(K_{2}x_{A}\right)}^{2}+{\left(K_{2}x_{A}\right)}^{3}+{\left(K_{2}x_{A}\right)}^{4}\right)}^{3/2}$$

This leads to the following region (aka ‘site’) balance:

12 $$1=\left[S\right]+\left[\mathrm{AS}\right]+\left[A_{2}S\right]+\left[A_{3}S\right]+...\left[A_{m+1}S\right]$$

13 $$1=\left[S\right]+{\left(K_{1}x_{A}\right)}^{\alpha }\left[S\right]\left(1+\left(K_{2}x_{A}\right)+{\left(K_{2}x_{A}\right)}^{2}+...{\left(K_{2}x_{A}\right)}^{m-1}+{\left(K_{2}x_{A}\right)}^{m}\left(1+\left(\frac{m}{m+1}\right)\left(\mathrm{C.T.}-1\right)\right)\right)$$

14 $$1=\left[S\right]+{\left(K_{1}x_{A}\right)}^{\alpha }\left[S\right]\left(\frac{1-{\left(K_{2}x_{A}\right)}^{m+1}}{1-\left(K_{2}x_{A}\right)}+\left(\frac{m}{m+1}\right){\left(K_{2}x_{A}\right)}^{m}\left(\mathrm{C.T.}-1\right)\right)$$

Solving for [S] gives

15 $$\left[S\right]=\frac{1}{1+{\left(K_{1}x_{A}\right)}^{\alpha }\left(\frac{1-{\left(K_{2}x_{A}\right)}^{m+1}}{1-\left(K_{2}x_{A}\right)}+\left(\frac{m}{m+1}\right){\left(K_{2}x_{A}\right)}^{m}\left(\mathrm{C.T.}-1\right)\right)}$$

The adsorbed amount, Q_A_, of A on the surface is given by

16 $$Q_{A}=N\left(\left[\mathrm{AS}\right]+2\left[A_{2}S\right]+3\left[A_{3}S\right]+...+\left(m+1\right)\left[A_{m+1}S\right]\right)$$

where N is the number of adsorption regions. Substituting eqns [Disp-formula eq4], 8, and 10 into eqn [Disp-formula eq16] gives

17 $$Q_{A}=N{\left(K_{1}x_{A}\right)}^{\alpha }\left[S\right]\left(1+2\left(K_{2}x_{A}\right)+3{\left(K_{2}x_{A}\right)}^{2}+...m{\left(K_{2}x_{A}\right)}^{m-1}+\left(m+1\right){\left(K_{2}x_{A}\right)}^{m}\left(1+\left(\frac{m}{m+1}\right)\left(\mathrm{C.T.}-1\right)\right)\right)$$

which can be rewritten as

18 $$Q_{A}=N{\left(K_{1}x_{A}\right)}^{\alpha }\left[S\right]\left(\frac{1}{K_{2}}\frac{d}{{\mathrm{dx}}_{A}}\left(\frac{1-{\left(K_{2}x_{A}\right)}^{m+2}}{1-\left(K_{2}x_{A}\right)}\right)+m{\left(K_{2}x_{A}\right)}^{m}\left(\mathrm{C.T.}-1\right)\right)$$

Evaluating this derivative yields

19 $$Q_{A}=N{\left(K_{1}x_{A}\right)}^{\alpha }\left[S\right]\times \left(\frac{1-\left(m+2\right){\left(K_{2}x_{A}\right)}^{m+1}+\left(m+1\right){\left(K_{2}x_{A}\right)}^{m+2}}{{\left(1-\left(K_{2}x_{A}\right)\right)}^{2}}+m{\left(K_{2}x_{A}\right)}^{m}\left(\mathrm{C.T.}-1\right)\right)$$

To make this analysis as general as possible, we allow for the possibility of multiple sitegroups that have different Manz-Delgass (MD) model isotherm parameters values, as described in Section 2.3 below. Let α_g_, K1_g_, K2_g_, m_g_, and N_g_ be the parameter values for the g^th^ sitegroup. Substituting eqn [Disp-formula eq15] into eqn [Disp-formula eq19] then gives

20 $$Q_{A,g}^{MD}=N_{g}\frac{{\left(K1_{g}x_{A}\right)}^{\alpha_{g}}\left(\frac{1-\left(m_{g}+2\right){\left(K2_{g}x_{A}\right)}^{m_{g}+1}+\left(m_{g}+1\right){\left(K2_{g}x_{A}\right)}^{m_{g}+2}}{{\left(1-\left(K2_{g}x_{A}\right)\right)}^{2}}+m_{g}{\left(K2_{g}x_{A}\right)}^{m_{g}}\left(\mathrm{C.T.}-1\right)\right)}{1+{\left(K1_{g}x_{A}\right)}^{\alpha_{g}}\left(\frac{1-{\left(K2_{g}x_{A}\right)}^{m_{g}+1}}{1-\left(K2_{g}x_{A}\right)}+\left(\frac{m_{g}}{m_{g}+1}\right){\left(K2_{g}x_{A}\right)}^{m_{g}}\left(\mathrm{C.T.}-1\right)\right)}$$

Here, N_g_ specifically means ‘the number of molecules of species A that can adsorb onto surface regions of type (i.e., sitegroup) g in the first adsorption step for that sitegroup’. Let S_g_ denote a surface region (aka ‘site’) of type g. To reiterate, N_g_ refers to the number of molecules of species A that can adsorb in the first adsorption step (i.e., A_j_S_g_ where j = 1), and it does not directly refer to an individual atom comprising the adsorbent surface.

Divisions by zero are avoided by using the following limits:

21 $$\underset{\left(K_{2}x_{A}\right)\to 1}{\lim}\left(\frac{1-{\left(K_{2}x_{A}\right)}^{m+1}}{1-\left(K_{2}x_{A}\right)}\right)=\left(m+1\right)$$

22 $$\underset{\left(K_{2}x_{A}\right)\to 1}{\lim}\left(\frac{1-\left(m+2\right){\left(K_{2}x_{A}\right)}^{m+1}+\left(m+1\right){\left(K_{2}x_{A}\right)}^{m+2}}{{\left(1-\left(K_{2}x_{A}\right)\right)}^{2}}\right)=\frac{\left(m+1\right)\left(m+2\right)}{2}$$

If m_g_ = 0, then Q_A,g_^MD^ reduces to the Langmuir isotherm (eqn [Disp-formula eq26]). If m_g_ > 0, then

23 $$\underset{\left(K_{2}x_{A}\right)\to \infty }{\lim}Q_{A,g}^{\mathrm{MD}}=\left(1+m_{g}\right)N_{g}$$

It follows that m_g_ should be physically interpreted as the adsorption capacity-averaged effective number of adsorption layers beyond the first layer. Accordingly, m_g_ should be treated as a non-negative continuous fitting parameter. For example, if the pore shape allows only half as many molecules to adsorb beyond the first layer as in the first layer, then completely filling the pores yields a total adsorbed amount of Q_A,g_^MD^ = 1.5N_g_. This means m_g_ = 0.5 for these pores. In practice, one does not perform experiments at infinite pressure (or infinite concentration), so the limit K_2_x_A_ → ∞ is an extrapolation, and the optimum m_g_ value is actually obtained through regression as described in Section 2.4 below.

### 2.2 Limiting Cases Related to Prior Adsorption Isotherms

In the extremely low-coverage limit, choosing α = 1 recovers the Henry’s isotherm^39^

24 $$Q_{A}^{\mathrm{Henry}}={\mathrm{NK}}_{1}x_{A}$$

In the same low-coverage limit, placing no restrictions on α recovers the Freundlich isotherm:^33,34^

25 $$Q_{A}^{\mathrm{Freundlich}}=N{\left(K_{1}x_{A}\right)}^{\alpha }$$

As shown in Figure 2, three basic kinds of Type 1 isotherms can be recovered from Q_A_^MD^. The Langmuir adsorption isotherm^40^ is recovered from Q_A_^MD^ by choosing m = 0 (or K_2_ = 0) and α = 1:

26 $$Q_{A}^{\mathrm{Langmuir}}=N\frac{K_{1}x_{A}}{1+K_{1}x_{A}}$$

The Langmuir isotherm assumes that adsorbates cannot stack. The Sips^35,36,41^ (aka Langmuir–Freundlich) isotherm is recovered by choosing m = 0 (or K_2_ = 0) without restricting α:

27 $$Q_{A}^{\mathrm{Sips}}=N\frac{{\left(K_{1}x_{A}\right)}^{\alpha }}{1+{\left(K_{1}x_{A}\right)}^{\alpha }}$$

Dissociative adsorption (e.g., $H_{2}+2S\overset{K_{H}}{↔}2\mathrm{HS}$ ) on an ideal isotropic surface corresponds to m = 0 (or K_2_ = 0) and α = 1/2:

28 $$Q_{A}^{H}=N\frac{\sqrt{K_{1}P_{H_{2}}}}{1+\sqrt{K_{1}P_{H_{2}}}}$$

**Figure 2:**
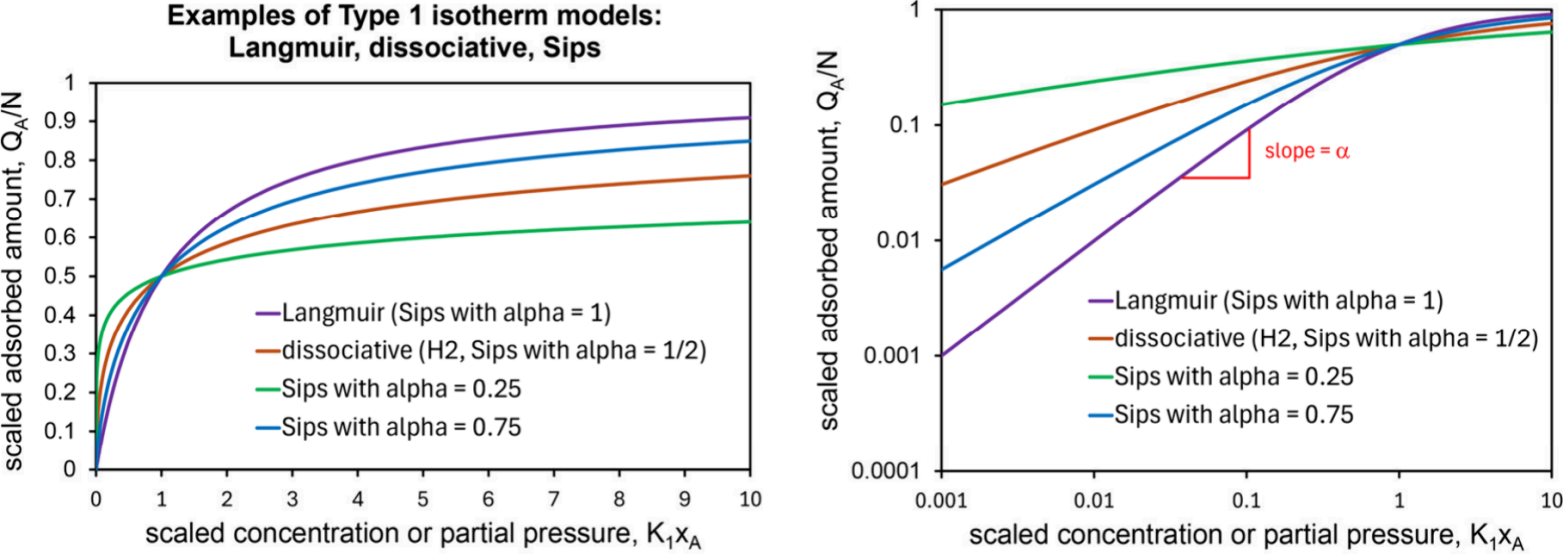
Examples of Type 1 isotherms that can be recovered from Q_A_^MD^. The left panel has linear axes while the right panel has logarithmic axes. The parameter α equals the initial slope on the log–log plot.

The Freundlich isotherm is the low-coverage limit of the Sips isotherm. At high coverages, the Sips isotherm approaches a saturation value, while the Freundlich isotherm does not. The Freundlich and Sips isotherms arise in several situations. Case #1: The Freundlich and Sips isotherms result by approximating the surface as heterogeneous with a distribution of varying surface site energies, where each individual site follows the Langmuir model.^41,42^ Case #2: The Freundlich isotherm results at low coverages when the adsorption and desorption kinetics have different orders.^34^ In some situations, the Sips isotherm results at all coverage values when the adsorption and desorption kinetics have different orders (see ref 34 and the dissociative H_2_ example in eqn [Disp-formula eq28] above). Case #3: Several authors used thermodynamic or statistical mechanical approaches to derive the Freundlich and Sips isotherms.^19,43–45^ These statistical mechanical approaches showed that the Freundlich and Sips isotherms can arise from lateral adsorbate–adsorbate repulsive interactions (even if all sites are homogeneous) and/or from energetic site heterogeneity.^19,43^

The BET adsorption isotherm^46^ is recovered from Q_A_^MD^ by choosing m → ∞, K_2_ = 1, α = 1, and x_A_ = p/p_0_, where p_0_ is the saturation pressure at the adsorption temperature:

29 $$Q_{A}^{\mathrm{BET}}=N\frac{K_{1}p/p_{0}}{\left(1-p/p_{0}\right)\left(1+\left(K_{1}-1\right)p/p_{0}\right)}$$

The BET isotherm assumes vertical stacking, such that molecules adsorbing in step 2 stack on top of molecules that already adsorbed in step 1. It also assumes parallel walls so that each vertically stacked layer has the same maximum capacity.

In the same 1938 article introducing the BET adsorption isotherm, Brunauer et al. also proposed an extension called the η-BET isotherm that limits the adsorption to a finite number (η) of vertically stacked layers.^46^ The η-BET isotherm can be written in the form:

30 $$Q_{A}^{\eta ‐\mathrm{BET}}=N\frac{K_{1}p/p_{0}}{\left(1-p/p_{0}\right)}\times \left(\frac{1-\left(\eta +1\right){\left(p/p_{0}\right)}^{\eta }+\eta {\left(p/p_{0}\right)}^{\eta +1}}{1+\left(K_{1}-1\right)\left(p/p_{0}\right)-K_{1}{\left(p/p_{0}\right)}^{\eta +1}}\right)$$

The η-BET isotherm assumes there is a finite maximum number (e.g., η) of vertically stacked layers.^46^ They also assumed parallel walls so that each vertically stacked layer has the same maximum capacity. The η-BET isotherm is not a special case of our Q_A_^MD^ isotherm, because the η-BET isotherm assumes that [A_η_S] = x_A_[A_η–1_S] instead of eqn [Disp-formula eq10].

A key limitation of the η-BET isotherm is that it does not properly describe capillary condensation.^1,47^ Specifically, the η-BET isotherm predicts a material’s pores will be substantially less than completely filled in the limit (p/p_0_) → 1.^47^ In 1940, Brunauer, Deming, Deming, and Teller (aka ‘BDDT’) introduced two new isotherms that resolve this problem by introducing an additional term that depends on the adsorbed liquid’s surface tension, σ. In multilayer adsorption with capillary condensation, the heat of adsorption of the final layer differs from the preceding layers, because the last adsorbed layer reduces the liquid’s surface area. Consequently, the effective heat of adsorption for the last layer is something like the heat of liquefaction plus the surface tension times the surface area reduction.^1^ Using this idea, Brunuaer et al. introduced two new adsorption isotherms (equations D and E of their 1940 article) that include capillary condensation.^1^ However, as the equations for those two isotherms are extremely complicated, they are not reproduced here.

Pickett proposed a mathematically simpler approach to including capillary condensation.^47^ Pickett proposed modifying the expression [A_η_S] = x_A_[A_η–1_S] (used in the η-BET isotherm) for the last adsorption step to [A_η_S] = x_A_[A_η–1_S]/(1 – x_A_). This forces complete capillary condensation (i.e., filling of the last layer) as x_A_ → 1. Pickett’s adsorption isotherm can be written as

31 $$Q_{A}^{\mathrm{Pickett}}=N\frac{K_{1}p/p_{0}}{\left(1-p/p_{0}\right)}\left(\frac{1-{\left(p/p_{0}\right)}^{\eta }}{1+\left(K_{1}-1\right)\left(p/p_{0}\right)}\right)$$

Pickett’s eqn [Disp-formula eq31] cannot be used for supercritical fluids, because supercritical fluids do not have a saturation vapor pressure. For this reason, we chose a different (see eqns [Disp-formula eq10] and 11) condensation term.

In 1946, Anderson proposed several modifications and extensions to the BET isotherm.^48^ One of these extensions handles the case in which the heat of adsorption for the second and subsequent layers has a smaller magnitude than the heat of liquefaction.^48^ In our notation, this corresponds to the situation for which m → ∞, 0 < K_2_ < 1, α = 1, and x_A_ = p/p_0_:

32 $$Q_{A}^{\mathrm{Anderson}}=N\frac{K_{1}x_{A}}{\left(1-K_{2}x_{A}\right)\left(1+\left(K_{1}-K_{2}\right)x_{A}\right)}$$

Anderson also proposed an extension to the BET isotherm in which the maximum capacity of each subsequent layer is some fraction 0 < f < 1 of the previous layer. This yielded the isotherm^48^

33 $$Q_{A}^{\mathrm{Anderson}}=\frac{V_{\mathrm{monolayer}}{\mathrm{cx}}_{A}}{\left(1-{\mathrm{fx}}_{A}\right)\left(1+\left(c-1\right)x_{A}\right)}$$

Close examination shows that eqns [Disp-formula eq32] and 33 are equivalent model isotherms if the following substitutions are made:

34 $$K_{2}=f$$

35 $$K_{1}=c-1+f$$

36 $$N=V_{\mathrm{monolayer}}c/K_{1}=V_{\mathrm{monolayer}}c/\left(c-1+f\right)$$

Anderson’s model isotherm is recovered from Q_A_^MD^ (eqn [Disp-formula eq20]) in the limit m → ∞ and α = 1, where K_2_x_A_ < 1. In 1948, Anderson and Hall^49^ proposed yet another form

37 $$Q_{A}^{\mathrm{Anderson}}=\frac{V_{m}{\mathrm{ckx}}_{A}}{\left(1-{\mathrm{jkx}}_{A}\right)\left(1+\left(c-j\right){\mathrm{kx}}_{A}\right)}$$

which again is equivalent to eqn [Disp-formula eq32] by making the substitutions N = V_m_, K_1_ = ck, K_2_ = jk.

The above discussion shows that 0 < K_2_ < 1 models both the situations for which (a) the heat of adsorption on second and subsequent layers has a smaller magnitude (i.e., is weaker) than the heat of liquefaction and (b) the maximum capacity of each subsequent layer is some fraction 0 < f < 1 of the previous layer. By extension, we can infer that K_2_ > 1 could model the situation in which the second and subsequent adsorption steps have stronger attractive interactions than the pure liquid phase; however, Anderson’s isotherm must be restricted to the region K_2_x_A_ < 1.

An adsorption isotherm similar to Q_A_^MD^ of eqn [Disp-formula eq20] except restricted to α = 1 and using [A_η_S] = K_2_x_A_[A_η–1_S] (instead of eqn [Disp-formula eq10]) was introduced by us in a 1998 thesis:^50^

38 $$Q_{A}^{\mathrm{Zeta}\text{\_}1998}=N\frac{K_{1}x_{A}}{1+K_{1}x_{A}\left(\frac{1-{\left(K_{2}x_{A}\right)}^{\eta }}{1-\left(K_{2}x_{A}\right)}\right)}\times \left(\frac{1-\left(\eta +1\right){\left(K_{2}x_{A}\right)}^{\eta }+\eta {\left(K_{2}x_{A}\right)}^{\eta +1}}{{\left(1-\left(K_{2}x_{A}\right)\right)}^{2}}\right)$$

The η-BET isotherm is recovered from that 1998 isotherm in the limit K_2_ → 1. The Anderson isotherm is recovered from that 1998 isotherm in the limit η → ∞. However, we consider Q_A_^MD^ (eqn [Disp-formula eq20]) to be generally more accurate and easier to interpret than our 1998 isotherm, because eqn [Disp-formula eq10] accounts for condensation in the limit (K_2_x_A_) → 1. Our 1998 adsorption isotherm has a functional form equivalent to the Zeta isotherm relation derived in 2007 by Ward and Wu:^51^

39 $$Q_{A}^{\mathrm{Zeta}\text{\_}2007}=\frac{{\mathrm{Mc}\alpha }_{Z}x^{V}\left(1-\left(1+\zeta \right){\left(\alpha_{Z}x^{V}\right)}^{\zeta }+\zeta {\left(\alpha_{Z}x^{V}\right)}^{1+\zeta }\right)}{\left(1-\alpha_{Z}x^{V}\right)\left(1+\left(c-1\right)\alpha_{Z}x^{V}-c{\left(\alpha_{Z}x^{V}\right)}^{1+\zeta }\right)}$$

Close examination reveals that Q_A_^Zeta_1998^ and Q_A_^Zeta_2007^ are completely equivalent and the same model isotherm. This examination shows that the two representations of the same isotherm are translated via M = N, α_Z_ = K_2_ c = K_1_/K_2_, ζ = η, x^V^ = x_A_.

Figure 3 illustrates these various model isotherms. The BET isotherm is a special case of each of the other isotherms illustrated Figure 3. A key advantage of our isotherm is that it covers Types 1, 2, 3, 4, 5, and even Type 6 (see Sections 2.3 and 3.4 below) in a manner that is easy to understand and interpret. Table 1 compares key properties of these adsorption model isotherms.

**Table 1:** Comparison of Key Properties of Adsorption Isotherms That Include the BET Isotherm as a Special Case

isotherm name	ref.	num. adjustable parameters	includes case with limited multilayers?	isotherm types^a^	includes capillary condensation?	includes lateral interactions and surface heterogeneity?
BET	^46^	2	no	1, 2, 3	no	no
η-BET	^46^	3	yes	1, 2, 3	no	no
BDDT	^1^	4	yes	1, 2, 3, 4, 5	yes	no
Pickett	^47^	3	yes	1, 2, 3	yes	no
Anderson	^48^	3	no	1, 2, 3	no	no
Zeta	^50^,^51^	4	yes	1, 2, 3, 4, 5	no	no
Manz-Delgass	this work	5	yes	1, 2, 3, 4, 5	yes	yes

a Type 6 isotherms can be modeled using multiple sitegroups.

**Figure 3:**
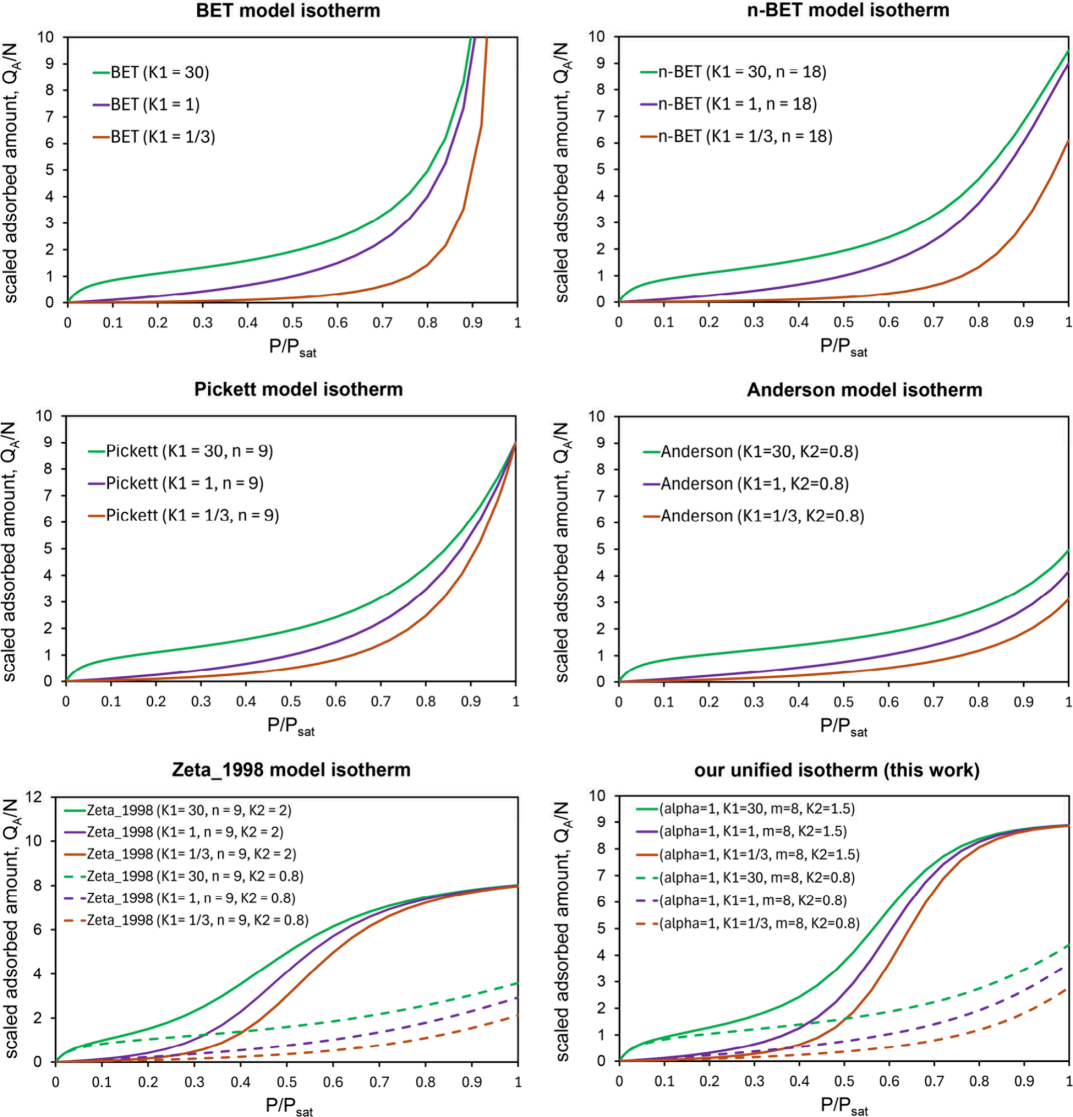
Example multilayer adsorption isotherm shapes generated by the BET, η-BET, Pickett, Anderson, Zeta, and this work’s MD (see eqn [Disp-formula eq20]) model isotherm equations. In this illustration, the lowercase ‘n’ is another name for the parameter ‘η’.

Some of the above-mentioned isotherms have been used to model water adsorption on materials. In this case,

40 $$x_{A}=a_{W}=P_{H_{2}O}/P_{H_{2}O}^{\mathrm{sat}}$$

is the water activity at the adsorption temperature. The Guggenheim, Anderson and de Boer (GAB) isotherm, which is often used to model water adsorption onto food and foodstuffs,^52,53^ has the form

41 $$Q_{A}^{\mathrm{GAB}}=Q_{\mathrm{monolayer}}^{\mathrm{GAB}}\frac{c_{G}{\mathrm{ka}}_{W}}{\left(1-{\mathrm{ka}}_{W}\right)\left(1-\left(c_{G}-1\right){\mathrm{ka}}_{W}\right)}$$

This is equivalent to the Anderson isotherm shown in eqn [Disp-formula eq32] by making the following substitutions: Q_monolayer_^GAB^ = N, K_1_ = c_G_k, and K_2_ = k. Moreover, the GAB isotherm is a limiting case of our Q_A_^MD^ isotherm (see eqn [Disp-formula eq20]) using α = 1, m → ∞, Q_monolayer_^GAB^ = N, K_1_ = c_G_k, and K_2_ = k, where the GAB isotherm must be restricted to the region ka_W_ < 1. In addition to the GAB isotherm, many other model adsorption isotherms are available to model moisture adsorption on materials.^13,54–58^

### 2.3 Multiple Sitegroups

A sitegroup is a collection of individual site (region) types that are grouped together and described by a single set of MD model isotherm parameter values. All sites within the same sitegroup have the same value of α, K_1_, K_2_, m, and N.

Occasionally, individual site (region) types have physical and/or chemical properties so different that they need to be classified into different sitegroups, where each sitegroup has its own MD model isotherm parameter values. Examples of this situation include the following:

(a) In some materials, there are different classes of pores: micropores (<2 nm in diameter), mesopores (2–50 nm in diameter), and macropores (>50 nm in diameter). In gas physisorption, micropores typically fill at low P/P_0_ values. In gas physisorption, mesopores typically fill at intermediate P/P_0_ values and exhibit adsorption–desorption hysteresis due to capillary condensation. In gas physisorption, macropores typically fill at P/P_0_ close to 1. Many materials have both micropores and mesopores, and it is sometimes necessary to use one sitegroup for micropores and another sitegroup for mesopores. For example, a powdered sample of small nanoporous crystals has micropores within each crystal and mesopores (or macropores) between the crystals.
(b) At submonolayer coverages, the adsorption isotherm may exhibit noticeable distinct steps if the distribution of surface-molecule (i.e., A + S → AS) adsorption energies is bimodal or multimodal. This can occur if one group of surface sites has adsorption energies that are much stronger than a different group of surface sites. In such case, it can be beneficial to fit the adsorption isotherm using two (or more) MD model sitegroups. Some heterogeneous materials are composites that have vastly different surface types owing to the multiple materials comprising the composite. For example, some heterogeneous catalysts contain metal particles supported on an oxide material. In these materials, it may be beneficial to model the metal surfaces as a first sitegroup and the oxide support surfaces as a second sitegroup. Plant-based materials, foods, and foodstuffs can also have heterogeneous surfaces that may necessitate using more than one sitegroup.
(c) Even if the surface and pore structure are uniform, in some cases the gas physisorption isotherm may exhibit multiple distinct steps due to layer-by-layer adsorption leading to Type VI isotherms as described in an IUPAC report.^2^ In this case, multiple sitegroups could be used in the MD model isotherm to reproduce the experimental isotherm’s multistep shape. An example is presented below in Section 3.4.

The full adsorption isotherm is the sum over all sitegroups. Using subscript g to represent a sitegroup, this leads to the following equation

42 $$Q_{A}^{\mathrm{MD}}=\overset{N_{\mathrm{sitegroups}}}{\underset{g=1}{\sum }}Q_{A,g}^{\mathrm{MD}}\left[\alpha_{g},K1_{g},K2_{g},m_{g},N_{g}\right]$$

where Q_A,g_^MD^[α_g_,K1_g_,K2_g_,m_g_,N_g_] is defined in eqn [Disp-formula eq20].

### 2.4 Method for Fitting the Model Isotherm to Experimental Data

Traditional error measures^3,4,12^ such as root-mean-squared error (RMSE), mean absolute error (MAE), mean absolute relative error (MARE), and R-squared (i.e., the correlation coefficient squared) are not optimal measures for fitting whole adsorption isotherms. RMSE and MAE penalize large deviations in the adsorbed amounts, and this ignores relative deviations. For example, an error of 0.2 mmol/g in the model-predicted adsorbed amount should be counted less significant in parts of the isotherm where the experimentally measured adsorbed amount is ∼10 mmol/g than where it is 0.01 mmol/g. On the other hand, if one uses a relative error measure (such as MARE), then this leads to instabilities (due to division by zero) for pressures where the experimentally measured adsorbed amount is negligible.

A comprise between the absolute error measure and the relative error measure can be achieved by defining a new point-wise error as (sqrt­[Q_model_] – sqrt­[Q_exp_])^2^. Consider the two pairs: (a) Q_model_ = 0.09, Q_exp_ = 0.16 and (b) Q_model_ = 0.64, Q_exp_ = 0.81. The former pair has a larger relative error but a smaller absolute error than the latter pair. Both pairs have the same value of (sqrt­[Q_model_] – sqrt­[Q_exp_])^2^. In contrast to relative error, this definition of the point-wise error never blows up, because it avoids division by zero even if Q_exp_ = 0.

Another issue that arises concerns the selection of pressure points for experimental measurements. If every experimentally used pressure point is weighted equally during the regression (i.e., model-fitting) procedure, then the model-fitting process will be prejudiced by which pressures were used in the experiments. Suppose experimentalist #1 uses 10 pressures between 3 and 4 bar and 20 pressures between 4 and 5 bar, while experimentalist #2 uses 20 pressures between 3 and 4 bar and 10 pressures between 4 and 5 bar. If every experimentally used pressure point is weighted equally during the regression (i.e., model-fitting) procedure, then experimentalist #1’s fitting results will put more emphasis on how closely the model isotherm fits the experimental isotherm over the pressure region 4–5 bar than over the pressure region 3–4 bar. In contrast, experimentalist #2’s fitting results will put more emphasis on how closely the model isotherm fits the experimental isotherm over the pressure region 3–4 bar than over the pressure region 4–5 bar.

To avoid this problem, the fitting procedure should be based on a total integrated error measure, Γ. By using such a total integrated error measure, experimentalist #1’s fitting results will place the same emphasis on the pressure region 3–4 bar as experimentalist #2’s fitting results. This makes the fitting result’s emphasis on each pressure region independent of how many experimental pressure values were used within that pressure region.

Depending on the material, the adsorbed amount may rise steeply at low pressures (e.g., around x_A_ = P/P_0_ = ∼10^–3^ to 10^–2^ as occurs for IRMOF-1 in Figure 9) or only when x_A_ = P/P_0_ reaches ∼0.4 (as occurs for water adsorption on Cr-MIL-101-NO_2_ as described in ref 59). To bring this two to 3 orders of magnitude difference in x_A_ onto a comparable scale, it is useful to use (x_A_)^1/3^ as the abscissa for integration. This ensures a robust error sampling irrespective of the particular pressure value at which the adsorption isotherm rises steeply. Moreover, plotting the adsorption isotherm with (x_A_)^1/3^ values on the horizontal axis allows the low-pressure region and high-pressure region to be accurately and simultaneously visualized on a single plot without having to create separate linear-scale and log-scale plots.

Putting this altogether, we define the fitscore as

43 $$\mathrm{fitscore}=\frac{\sqrt{\Lambda }}{\sqrt{\Lambda }+\sqrt{\Gamma }}$$

$$\Gamma =\int_{a}^{b}{\left(\sqrt{Q_{\exp}}-\sqrt{Q_{\mathrm{model}}}\right)}^{2}d\left(\sqrt[{3}]{x_{A}}\right)\geq 0$$ 44

$$\Lambda =\int_{a}^{b}Q_{\exp}d\left(\sqrt[{3}]{x_{A}}\right)\geq 0$$ 45

where the square roots are always taken to be ≥ 0. Γ is the total integrated error measure. In eqn [Disp-formula eq44], Γ is computed by integrating the error measure from the minimum value of (x_A_)^1/3^ to the maximum value of (x_A_)^1/3^. Most often, this corresponds to a = 0 and b = 1; however, there are situations in which a and b can have values that differ from these. Here, Q_A_ is always the absolute adsorption amount (never the excess adsorption amount) which is always non-negative:

46 $$Q_{\mathrm{model}},Q_{\exp}\geq 0$$

With these definitions, it directly follows that the fitscore value is always between zero and one:

47 0≤fitscore≤1

Fitscore = 0 means the model is infinitely bad. Fitscore = 1 means the model exactly reproduced the experimental data points. In practice, a fitscore ≥ 0.95 is good, and a fitscore ≥ 0.99 is excellent.

Since the adsorption data is in the form of tabulated points that may be unequally spaced, the integrals in eqns [Disp-formula eq44] and 45 are computed using multitrapezoid integration:

48 $$\Gamma =\frac{1}{2}\left({\left(\sqrt{Q_{\exp,1}}-\sqrt{Q_{\mathrm{model},1}}\right)}^{2}\left(\sqrt[{3}]{x_{A,2}}-\sqrt[{3}]{x_{A,1}}\right)+\overset{N_{\mathrm{points}}-1}{\underset{i=2}{\sum }}{\left(\sqrt{Q_{\exp,i}}-\sqrt{Q_{\mathrm{model},i}}\right)}^{2}\left(\sqrt[{3}]{x_{A,\left(i+1\right)}}-\sqrt[{3}]{x_{A,\left(i-1\right)}}\right)+{\left(\sqrt{Q_{\exp,N_{\mathrm{points}}}}-\sqrt{Q_{\mathrm{model},N_{\mathrm{points}}}}\right)}^{2}\left(\sqrt[{3}]{x_{A,N_{\mathrm{points}}}}-\sqrt[{3}]{x_{A,\left(N_{\mathrm{points}}-1\right)}}\right)\right)$$

49 $$\Lambda =\frac{1}{2}\left(Q_{\exp,1}\left(\sqrt[{3}]{x_{A,2}}-\sqrt[{3}]{x_{A,1}}\right)+\overset{N_{\mathrm{points}}-1}{\underset{i=2}{\sum }}Q_{\exp,i}\left(\sqrt[{3}]{x_{A,\left(i+1\right)}}-\sqrt[{3}]{x_{A,\left(i-1\right)}}\right)+Q_{\exp,N_{\mathrm{points}}}\left(\sqrt[{3}]{x_{A,N_{\mathrm{points}}}}-\sqrt[{3}]{x_{A,\left(N_{\mathrm{points}}-1\right)}}\right)\right)$$

We define the optimal values of the MD isotherm parameters as those values that maximize the fitscore subject to positivity bounds on all model parameters:

50 $$\alpha_{g},K1_{g},K2_{g},m_{g},N_{g}\geq 0$$

This defines a nonlinear optimization problem with positivity bounds that can readily be solved using standard methods. For the reasons described above, the MD model isotherm parameters should always be fitted to maximize the fitscore, and they should never be fitted to optimize the MAE, MARE, RMSE, or R-squared value of the unscaled Q values.

Since Λ is a constant for a specific experimental data set, maximizing the fitscore (see eqn [Disp-formula eq43]) corresponds to minimizing Γ. Examining eqn [Disp-formula eq48] shows that Γ is a weighted sum of squared errors

51 $$\Gamma =\overset{N_{\mathrm{points}}}{\underset{i=1}{\sum }}w_{i}{\left(y_{\exp,i}-y_{\mathrm{pred},i}\right)}^{2}$$

where the observation variables are

52 $$y_{\mathrm{pred},i}=\sqrt{Q_{\mathrm{model},i}}$$

53 $$y_{\exp,i}=\sqrt{Q_{\exp,i}}$$

and the observation weights are

54 $$w_{1}=\frac{1}{2}\left(\sqrt[{3}]{x_{A,2}}-\sqrt[{3}]{x_{A,1}}\right)$$

55 $$w_{1<i<N_{\mathrm{points}}}=\frac{1}{2}\left(\sqrt[{3}]{x_{A,\left(i+1\right)}}-\sqrt[{3}]{x_{A,\left(i-1\right)}}\right)$$

56 $$w_{N_{\mathrm{points}}}=\frac{1}{2}\left(\sqrt[{3}]{x_{A,N_{\mathrm{points}}}}-\sqrt[{3}]{x_{A,\left(N_{\mathrm{points}}-1\right)}}\right)$$

This has the form of a standard nonlinear least-squares fitting problem.

For optimizing the MD isotherm parameters when using one sitegroup, we used the fmincon solver in Matlab. Please see Supporting Information Section S6 for a discussion of the program and solver settings.

For optimizing the Sips and Langmuir model parameters, we used the Generalized Reduced Gradient (GRG) solver in Excel with central finite derivatives, non-negative constraints on all model parameters using a constraint precision of 10^–6^, and maximization of the fitscore value using a convergence tolerance of 10^–6^. This GRG solver was also used when the MD model used two sitegroups. For some systems using one sitegroup, the GRG solver in Excel was also tested with these settings to optimize the MD isotherm parameters and found to give results that agree to several decimal places with the Matlab implementation described above. The Matlab implementation was primarily used for the MD isotherm with one sitegroup, because this implementation facilitated computing the 95% confidence intervals using the bootstrap method described in Section 2.6.

The model fitting process proceeds as follows. First, the experimental adsorption isotherm data is tabulated. Then, appropriate initial guesses for the MD model parameters α, K1, K2, m, and N are formulated using one sitegroup. The model parameter estimates are then refined by the solver until the convergence criteria are met. The best local optimum is the one that achieves the highest fitscore value. A plot is then used to compare the fitted model to the experimental isotherm. If the agreement is satisfactory, then the process concludes.

Otherwise, a second sitegroup is added to the model. In this case, it is necessary to compose a suitable initial guess of the parameters α_g_, K1_g_, K2_g_, m_g_, and N_g_ for g = 1, 2. One or more of the parameters α_g_, K1_g_, K2_g_, m_g_ must be different for g = 1 than for g = 2; otherwise, both sitegroups would be identical (which would make the second sitegroup irrelevant). After formulating the initial guess, the model parameter estimates are then refined by the solver until the convergence criteria are met. This optimization process maximizes the fitscore, and a plot is used to compare the fitted model to the experimental isotherm. If the agreement is satisfactory, then the process concludes.

In the vast majority of cases, only one or two sitegroups are needed. If, however, three or more sitegroups are needed, then the process proceeds analogously to that described above for two sitegroups, except initial guesses must be prepared for all three or more sitegroups. Some of the parameters must be different for each sitegroup.

Caution should employed when using two or more sitegroups to fit an adsorption isotherm, because this can potentially lead to an overfitting problem. The overfitting problem can arise due to the large number of parameters. Specifically, for the MD isotherm two site-groups involve a total of ten adjustable parameters. It might be advantageous to reduce the number of adjustable parameters; however, it is sometimes unclear how to do this in the best way. Using all ten adjustable parameters will cause the model isotherm to closely track the experimental adsorption isotherm, but owing to the multicollinearity (aka ‘overfitting’) issue one should not overinterpret (i.e., ‘read too much into’) the specific values of these ten optimized parameters.

Please see Section 2.6 below for a bootstrapping method to compute confidence intervals on the parameter values.

### 2.5 Whole Isotherm Surface Areas

Cryogenic N_2_ (at 77 K) or Ar (at 87 K) adsorption is especially convenient for extracting experimentally measured surface areas.^2^ Water adsorption is sometimes used to quantify the surface areas of minerals.^60,61^

After the MD model parameters are optimized as described in the preceding section, the whole isotherm surface area is defined as

57 $$\mathrm{SA}\;\left(\mathrm{in}\;m^{2}/g\right)=602.214\overset{N_{\mathrm{sitegroups}}}{\underset{g=1}{\sum }}N_{g}\sigma_{g}$$

Here, σ_g_ is the effective cross-sectional area of the adsorbing molecule in nm^2^, which is often the same for all g values. In eqn [Disp-formula eq57], N_g_ is expressed in mmol/g. The prefactor 602.214 equals Avogadro’s number (6.02214 × 10^23^ particles/mol) times 10^–18^ m^2^/nm^2^ times 10^–3^ mol/mmol.

For N_2_ adsorption at 77 K, σ = 0.162 nm^2^ is often used.^2^ For Ar adsorption at 87 K, σ = 0.142 nm^2^ is often used.^2^ For water adsorption at room temperature, σ = 0.106 nm^2^ ^61,62^ or 0.114 nm^2^ ^31,63^ are sometimes used, although the effective cross-sectional area for a water molecule may vary (e.g., 0.079–0.165 nm^2^/molecule^60^). Blattmann and Plötze published a comparison of BET-derived surface areas for minerals using N_2_ versus water physisorption.^60^ Large differences are sometimes observed, especially in layered materials when one adsorbate molecule (e.g., water) penetrates between layers more effectively than another adsorbate molecule (e.g., N_2_).^60,61^

In computations designed to compute whole isotherm surface areas, the K_2_ value should be constrained within a physically reasonable range. For the pure bulk liquid, K_2_ = 1 by definition, because x_A_ = P/P_sat_ has the value x_A_ = 1 for the pure bulk liquid. For multilayer adsorption in a porous material, K_2_ may differ moderately from unity. For example, if K_2_ = 1.5, this means vapor pressure is reduced due to capillary action in the pores relative to the pure bulk liquid’s vapor pressure. In this work, the following constraint on K_2_ was used when computing whole isotherm surface areas:

58 $$\left(2/3\right)\leq K_{2}\leq \left(3/2\right)$$

Computational tests showed this constraint improves the reliability of whole isotherm surface area calculation. In these adsorption experiments for measuring surface areas, the highest experimentally measured x_A_ value always corresponds to more than half a monolayer of coverage, so the MD isotherm parameter N was constrained to be less than or equal to twice the highest experimentally measured Q_A_ value. These two constraints apply only when multilayer adsorption with surface area computation is included, such as for N_2_ adsorption at 77 K, Ar adsorption at 87 K, and room temperature moisture adsorption.

What is the sensitivity of the computed whole isotherm surface area to the specific form of the condensation term? The condensation term is approximately 1 for K_2_x_A_ ≪ 1 and becomes larger for larger K_2_x_A_ values. Accordingly, the specific form of the condensation term was selected mainly to fit adsorption at relatively large x_A_ values where the adsorption isotherm approaches saturation, while the whole isotherm surface area is mostly determined near the monolayer coverage. Thus, while the specific form of the condensation term is important for fitting the high coverage limit, it typically does not have a huge effect on the computed whole isotherm surface area value.

### 2.6 Estimating Confidence Intervals

The following bootstrapping algorithm was used to estimate confidence intervals on the parameter values and whole isotherm surface areas. Let ỹ_i_^j^ denote the bootstrapped value for y_exp,i_, where the index j runs from j = 1 to N_bootstrap_. This starts at the point $\sqrt[{3}]{x_{A,1}}=0$ with

59 $${ỹ}_{1}^{j}=\sqrt{Q_{\exp,1}}=0$$

For i = 2 to (N_data_ −1), the step size is chosen to be the smallest absolute value among the following:

60 $${\mathrm{stepsize}}_{i}=\min\left[\begin{array}{l} \mathrm{abs}\left[y_{\exp,i}-y_{\exp,\left(i-1\right)}\right],\\ \mathrm{abs}\left[y_{\exp,\left(i+1\right)}-y_{\exp,i}\right],\\ \mathrm{abs}\left[y_{\exp,i}-y_{\mathrm{pred},i}\right]\\ y_{\exp,i} \end{array}\right]$$

For the last data point,

61 $${\mathrm{stepsize}}_{N_{\mathrm{data}}}=\min\left[\begin{array}{l} \mathrm{abs}\left[y_{\exp,N_{\mathrm{data}}}-y_{\exp,\left(N_{\mathrm{data}}-1\right)}\right],\\ \mathrm{abs}\left[y_{\exp,N_{data}}-y_{\mathrm{pred},N_{\mathrm{data}}}\right]\\ y_{\exp,N_{\mathrm{data}}} \end{array}\right]$$

The step Δ_i_^j^ is taken in a positive or negative direction

62 $$\Delta_{i}^{j}=\pm {\mathrm{stepsize}}_{i}$$

where the sign (+1 or −1) was randomly chosen by

63 sign⁡=(2round[rand]‐1)

Here, rand is a random number drawn from a uniform distribution over the interval [0,1), and round is a function that rounds to the nearest integer. For each Δ_i_^j^, a separate new random number is drawn to compute the sign.

The bootstrapped value was set to

64 $${ỹ}_{i}^{j}=y_{\exp,i}+\Delta_{i}^{j}$$

The inclusion of y_exp,i_ in the min function of eqns [Disp-formula eq60] and 61 guarantees that

65 $${ỹ}_{i}^{j}\geq 0$$

For a specific j value, ỹ_j,i_ for i = 1 to N_points_ was used in place of y_exp,i_ with the same $\left\{\sqrt[{3}]{x_{A,i}}\right\}$ values as before and refitted to the isotherm model (using the same procedure as described in Section 2.4 above) to obtain a set of model isotherm parameters β̃_j,k_ (i.e., α, K_1_, K_2_, m, and N). (For each iteration j, the starting guesses for the parameters were the optimized parameter values for the nonbootstrapped data set.) This procedure was repeated for j = 1 to N_bootstrap._ to obtain N_bootstrap_ values of each model parameter. The 95% confidence interval was then computed as the interval from the 2.5th percentile to the 97.5th percentile in the data set of N_bootstrap_ values for each model parameter.

The rationale for this algorithm is as follows. The stepsize defined above is an approximate estimate in the error of each data point. It is reasonable to believe that a meaningful new bootstrapped data set could be formed by randomly adding or subtracting this step to the existing experimental values. By randomly choosing a +1 (i.e., addition) or −1 (i.e., subtraction) sign, the new bootstrapped experimental data maintains the same expectation value as the original experimental data; in other words, this bootstrapping procedure is unbiased on average. If the fitted model slightly overshoots the experimental data at low pressures and slightly undershoots the experimental data at higher pressures, then we believe it is more correct to assume the experimental values provide a better representation of the ‘true’ behavior than the fitted model. This explains why our bootstrapping procedure adds the step to the original experimental values rather than to the original model values.

A few tests were performed to identify a suitable N_bootstrap_ value. These tests are listed in Table 2. For example, S.A. (m^2^/g) of (113, 128) for run 1 means that 95% of the bootstrapped values were between 113 and 128 m^2^/g. Since N_bootstrap_ = 5000 provided reasonable reproducibility at reasonable computational cost, it was used for all results reported in this work.

**Table 2:** Three Runs for Computing the 95% Confidence Intervals Using N_bootstrap_ = 5000^a^

	α	K_1_	K_2_	m	N	S.A. (m^2^/g)
run 1	(0.35, 0.63)	(578, 877)	(1.26, 1.35)	(1.74, 2.04)	(1.16, 1.31)	(113, 128)
run 2	(0.36, 0.62)	(577, 878)	(1.26, 1.35)	(1.74, 2.04)	(1.16, 1.31)	(114, 128)
run 3	(0.35, 0.62)	(578, 878)	(1.26, 1.35)	(1.74, 2.04)	(1.16, 1.31)	(114, 128)

a Results are for fitting the MD isotherm to N_2_ adsorption at 77 K on a porous Al–Ni–Fe–Cr material after leaching in concentrated aqueous sodium hydroxide, as described in Section 3.1.

### 2.7 Domain Restrictions and Basic Sanity Checks

This section derives some basic mathematical properties to show the MD model isotherm is well-behaved across physically allowable ranges. It also discusses some corner cases that require limiting values to be inserted. It is useful to note the condensation term (C.T. in eqn [Disp-formula eq11]) equals 1 when K_2_x_A_ = 0 and monotonically increases with increasing K_2_x_A_. This means (C.T. – 1) > 0 when K_2_x_A_ > 0.

First, a brief clarification on bounds for the model parameters. As shown in eqn [Disp-formula eq50], α, K_1_, K_2_, m, and N are non-negative. For physical reasons, α should be strictly positive (i.e., greater than zero), because (K_1_x_A_)^α^ would become constant if α = 0 and this would effectively nullify the first adsorption step. If the first adsorption step is negligible, then one should set K_1_ = 0 and α > 0. This corresponds to the null isotherm (Q_A_ = 0 for all x_A_), which is ruled out if adsorption occurs, so the looser constraint α ≥ 0 (see eqn [Disp-formula eq50]) usually works fine in the solver’s settings. For the upper bounds, α, K_1_, K_2_, and N should be finite (i.e., less than infinity), while m may equal infinity:

66 0<α<∞

67 $$0\leq N,K_{1},K_{2}<\infty$$

68 0≤m≤∞

Setting some of the parameter values to zero effectively ‘tunes out’ other parameters. Here, the phrase ‘tunes out’ means values of the other parameters become irrelevant. For example, setting N = 0 effectively tunes out all other parameters. Setting m = 0 effectively tunes out K_2_. Setting K_2_ = 0 effectively tunes out m.

*The behavior at x*_*A*_ *= 0:* (a) When x_A_ = 0 and 0 < m ≤ ∞, then all terms in eqn [Disp-formula eq20] are straightforward to evaluate and give Q_A_^MD^ = 0. (b) When x_A_ = 0 and m = 0, then eqn [Disp-formula eq20] involves a (K_2_x_A_)^m^ = 0^0^ subterm and although this may appear at first to be ambiguous (should 0^0^ = 0 or 1?) this apparent ambiguity is irrelevant because it is multiplied by (C.T. – 1) = 0. Hence, this becomes 0 × 0^0^ = 0. Thus, for any allowable parameter values (see eqns [Disp-formula eq66]–[Disp-formula eq68]), Q_A_^MD^ is zero at x_A_ = 0.

*What is the range of input x*_*A*_ *values for which* *Q*_*A*_^*MD*^ *is well-defined?* (a) When m = ∞, then Q_A_^MD^ is only well-defined for 0 ≤ K_2_x_A_ < 1. In this case, the value of Q_A_^MD^ at K_2_x_A_ = 1 can be inferred as infinite if N, K_1_, α > 0. (b) When 0 < m < ∞, then the adsorption is limited to (1 + m) total steps and remains finite for all 0 ≤ K_2_x_A_ ≤ ∞. In this case, eqns [Disp-formula eq21] and 22 are used to evaluate specific terms when K_2_x_A_ = 1.

*What are the correct low coverage asymptotes?* These can be recovered by taking the limit x_A_ → 0 and retaining just the leading nonconstant term in the series expansions of numerator and denominator in eqn [Disp-formula eq20]. Using a series expansion gives

69 $$Q_{A,g}^{\mathrm{MD}}=N_{g}\frac{{\left(K1_{g}x_{A}\right)}^{\alpha_{g}}\left(1+2\left(K2_{g}x_{A}\right)+3{\left(K2_{g}x_{A}\right)}^{2}+...\left(m_{g}+1\right){\left(K2_{g}x_{A}\right)}^{m_{g}}+m_{g}{\left(K2_{g}x_{A}\right)}^{m_{g}}\left(\mathrm{C.T.}-1\right)\right)}{1+{\left(K1_{g}x_{A}\right)}^{\alpha_{g}}\left(1+\left(K2_{g}x_{A}\right)+{\left(K2_{g}x_{A}\right)}^{2}+...{\left(K2_{g}x_{A}\right)}^{m_{g}}+\left(\frac{m_{g}}{m_{g}+1}\right){\left(K2_{g}x_{A}\right)}^{m_{g}}\left(\mathrm{C.T.}-1\right)\right)}\geq 0$$

Retaining just the leading terms gives the low coverage limit:

70 $$Q_{A,g}^{MD}\left[x_{A}\approx 0\right]\approx N_{g}\frac{{\left(K1_{g}x_{A}\right)}^{\alpha_{g}}}{1+{\left(K1_{g}x_{A}\right)}^{\alpha_{g}}}$$

This low-coverage limit depends only on N_g_, K1_g_, and α_g_ and has the same form as the SIPS isotherm. Neglecting the second term in the denominator recovers the Freundlich isotherm.

As derived in Supporting Information Section S5.1, the value of Q_A,g_^MD^ is always non-negative. As demonstrated in Supporting Information Section S5.2, the value of Q_A,g_^MD^ always increases monotonically with increasing x_A_.

## 3 Results and Discussion

### 3.1 Liquid Nitrogen Adsorption in Porous Nickel Alloy Sponges

We prepared several sponge nickel catalysts by leaching Mn–Ni and Al–Ni alloys. Synthesis and characterization details of the Mn–Ni alloys and leached materials are described in refs 50 and 64 . The Mn–Ni alloys were leached in concentrated acetic acid aqueous solution. The Al–Ni alloys were made by W.R. Grace & Co. We leached the Al–Ni alloys in concentrated sodium hydroxide aqueous solution.^65–67^

Alloy compositions and leaching conditions are summarized in Table 3. All of these materials were thoroughly rinsed in deionized water after leaching and pumped to high vacuum prior to N_2_ adsorption. We previously reported the compositions of the after-leached Al–Ni-based catalysts in ref 68. Using the procedure described in Supporting Information Section S1, we measured N_2_ adsorption isotherms at 77 K. The MD model isotherm was then fitted to the experimental data using the procedure described in Section 2.4 above. The fitted MD model parameters, resulting whole isotherm surface areas, and fitscores are listed in Table 3. The BET isotherm was also fitted to the experimental data over limited pressure ranges using the BET Surface Identification (BETSI^32^) procedure. BETSI plots are presented in Supporting Information Section S3. The BET surface areas and fitscores are listed in Table 3.

**Table 3:** Comparison of MD Model Isotherm Parameters, Whole Isotherm Surface Areas, and Fitscores for Various Porous Nickel Alloy Sponges^a^

alloy	Mn–Ni	Mn–Ni	Al–Ni	Al–Ni–Fe	Al–Ni–Fe–Cr
**alloy**wt %	72.5% Mn, 27.5% Ni	72.5% Mn, 27.5% Ni	50% Al, 50% Ni^a^	48–50% Al, 48–50% Ni, 2% Fe^b^	48–50% Al, 48–50% Ni, 1.3% Fe, 1.4% Cr^c^
**leached in**	2.5 M aq. acetic acid	2.5 M aq. acetic acid	10 M aq. NaOH	10 M aq. NaOH	10 M aq. NaOH
**leaching T**	25 °C	25 °C	90 °C	90 °C	90 °C
**leaching time**	7 days	14 days	1 h	1 h	1 h
**N**_**2**_ **isotherm**	run 1 [run 2]	**run 1**	**run 1**	**run 1**	**run 1**
**alpha (unitless)**	0.49 [0.45]	0.44	0.43	0.67	0.48
	(0.41, 0.55)	(0.40, 0.49)	(0.40, 0.46)	(0.48, 0.95)	(0.35, 0.63)
**K**_**1**_ **(unitless)**	29 [15]	30	698	473	727
	(12, 36)	(15, 50)	(532, 890)	(342, 629)	(578, 877)
**K**_**2**_ **(unitless)**	1.5 [1.5]	0.78	1.08	1.10	1.31
	(1.5,1.5)	(0.74, 0.81)	(1.04, 1.12)	(1.06, 1.14)	(1.26, 1.35)
**m (unitless)**	0.67 [0.88]	3.9 × 10^4^ ^d^	4.9	5.4	1.9
	(0.58,0.98)		(4.6, 5.1)	(5.1, 5.7)	(1.7, 2.0)
**N** **(mmol/g)**	0.11 [0.13]	0.38	0.90	0.78	1.21
	(0.105,0.135)	(0.34, 0.43)	(0.86, 0.94)	(0.75, 0.84)	(1.16, 1.31)
**fitscore (unitless)**	0.967 [0.974]	0.955	0.980	0.981	0.994
**surface area** **(m** ^**2**^ **/g)**	11 [13]	37	87	76	118
	(10,13)	(33, 42)	(84, 92)	(73, 82)	(113, 128)
**BET**S.A. **(m** ^**2**^ **/g)**	10 [11]	32	91	84	126
**BET fitscore**	0.493 [0.493]	0.604	0.621	0.629	0.784

a The BET surface area and fitscore for the BET isotherm are also listed for comparison. The alloy compositions are given in wt % before leaching. The Al–Ni based alloys were from W.R. Grace & Company. The 95% confidence intervals are shown in parentheses. For the Mn–Ni alloy leached 7 days, confidence intervals for the two runs were combined to form the widest span.

b W.R. Grace product number 16151-40.

c W.R. Grace product number 14528-90B.

d W.R. Grace product number 15820-48.

e For this material, the m value is ‘large’ and its precise value does not significantly impact the adsorbed amount at any pressure.

Figure 4 shows the N_2_ adsorption isotherm, model fits, and partly leached Mn–Ni alloy structure. The partly leached material had a brick-like microstructure. Two independent runs of N_2_ adsorption were measured on the material after leaching 7 days in 2.5 M aqueous acetic acid at room temperature. This leaching procedure oxidizes and removes some of the Mn atoms from the Mn–Ni alloy, thereby creating a porous structure. The MD model isotherm provided a good fit to entire experimentally measured isotherms with fitscores of 0.967 (run 1) and 0.974 (run 2). As shown in Figure 4, the BET model isotherm fit only a limited pressure range and had low fitscores of 0.493 (same for run 1 and run 2) for the entire pressure range. For this material, the extracted surface areas were in the range of 10–13 m^2^/g. This material has a Type IV isotherm with a small but nonzero amount of hysteresis due to the presence of some mesopores that filled via capillary condensation; however, the vast majority of the adsorption was within micropores that are too small to experience capillary condensation.

**Figure 4:**
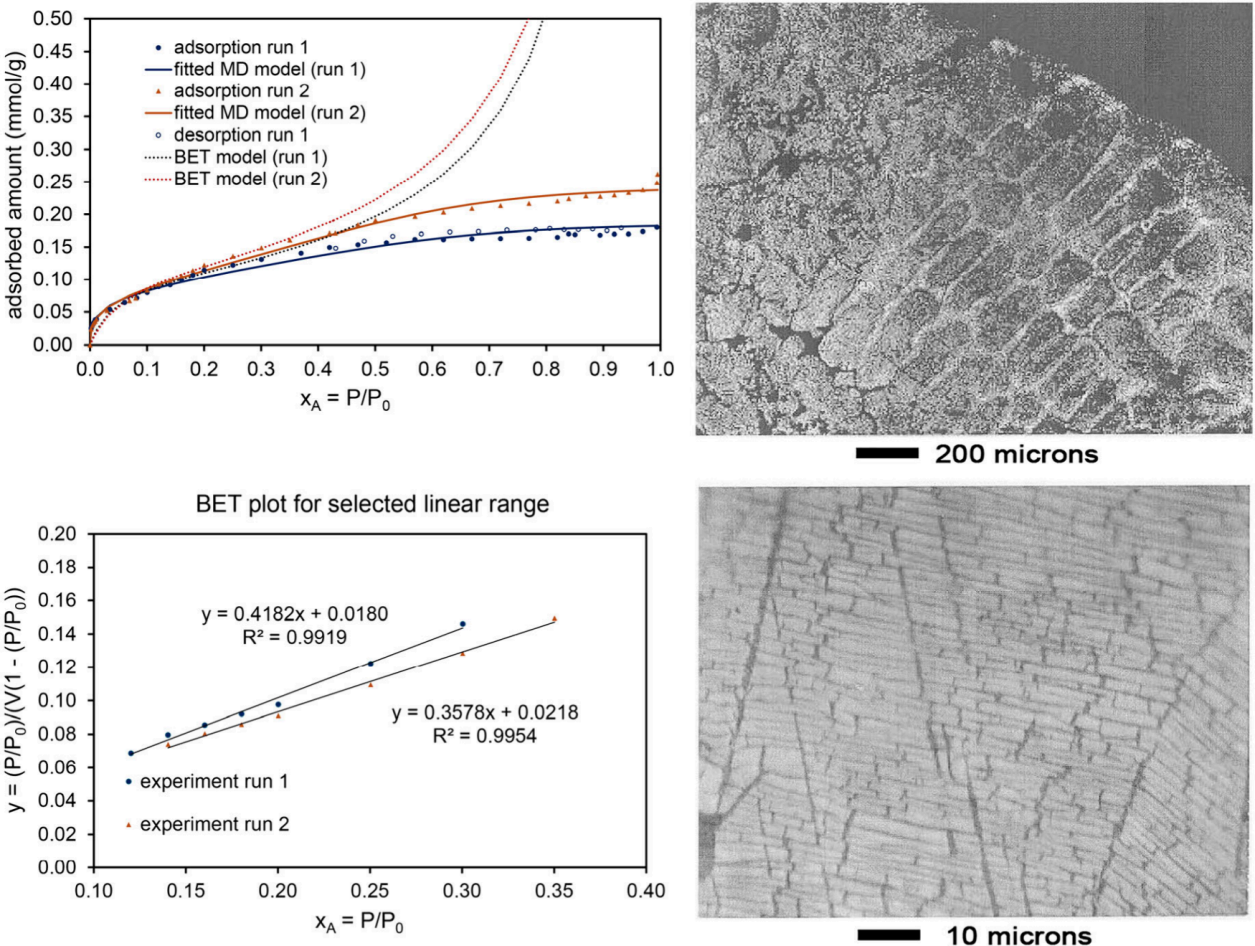
Analysis of N_2_ adsorption at 77.4 K on a porous Mn–Ni material after 7 days leaching in concentrated aqueous acetic acid. This material has a Type IV isotherm with a small but nonzero amount of hysteresis due to the presence of some mesopores that filled via capillary condensation; however, the vast majority of the adsorption was within micropores that are too small to experience capillary condensation. *Top left:* Comparison of fitted MD and BET model isotherms to experimental adsorption isotherms for two experimental runs. The MD model isotherm fits the experimental data much better than the BET model isotherm. *Bottom left:* Linear regression to determine the BET parameters. *Top right and lower right:* Optical micrographs showing the structure of the partially leached material after 10 min leaching in 1 wt % acetic acid aqueous solution.

Figure 5 shows the N_2_ adsorption isotherm model fits after leaching the Mn–Ni alloy for 14 days total in 2.5 M aqueous acetic acid at room temperature. In contrast to Figure 4, this long leaching time produced a porous Ni–Mn structure containing large open pores that had a Type II N_2_ adsorption isotherm at 77 K. As shown in Table 3, the MD whole isotherm (37 m^2^/g) and BET partial isotherm (32 m^2^/g) surface areas were similar. The MD model (fitscore = 0.955) provided a much better fit to the experimental data than the BET model (fitscore = 0.604).

**Figure 5:**
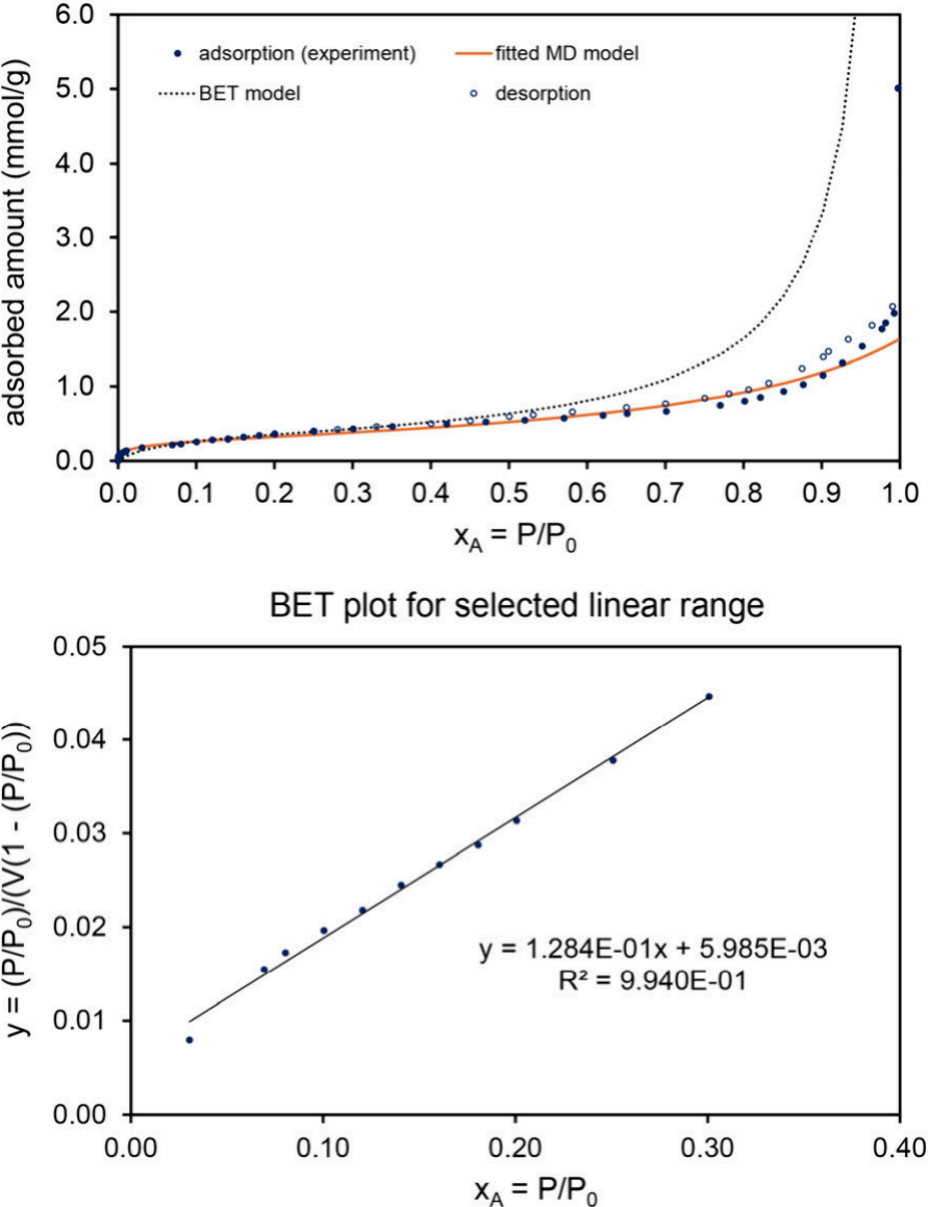
Analysis of N_2_ adsorption at 77.4 K on a porous Mn–Ni material after leaching in concentrated aqueous acetic acid for 14 days. These results suggest a structure containing large open pores. *Top panel:* Comparison of fitted MD and BET model isotherms to the experimental adsorption isotherm. The MD model isotherm fits the experimental data much better than the BET model isotherm. *Bottom panel:* Linear regression to determine the BET parameters.

Figure 6 shows the shows the N_2_ adsorption isotherm, model fits, and partly leached Al–Ni alloy structure. The partly leached material had a barnacle-like microstructure. N_2_ adsorption was measured on the material after leaching 1 h in 10 M aqueous sodium hydroxide at 90 °C. The MD model isotherm provided a good fit to the entire experimentally measured isotherm with a fitscore of 0.980. As shown in Figure 6, the BET model isotherm fit only a limited pressure range and had a low fitscore of 0.621 for the entire pressure range. The MD whole isotherm surface area of 87 m^2^/g was similar to the BET surface area of 91 m^2^/g. This material had a Type IV isotherm with hysteresis due to capillary condensation in mesopores.

**Figure 6:**
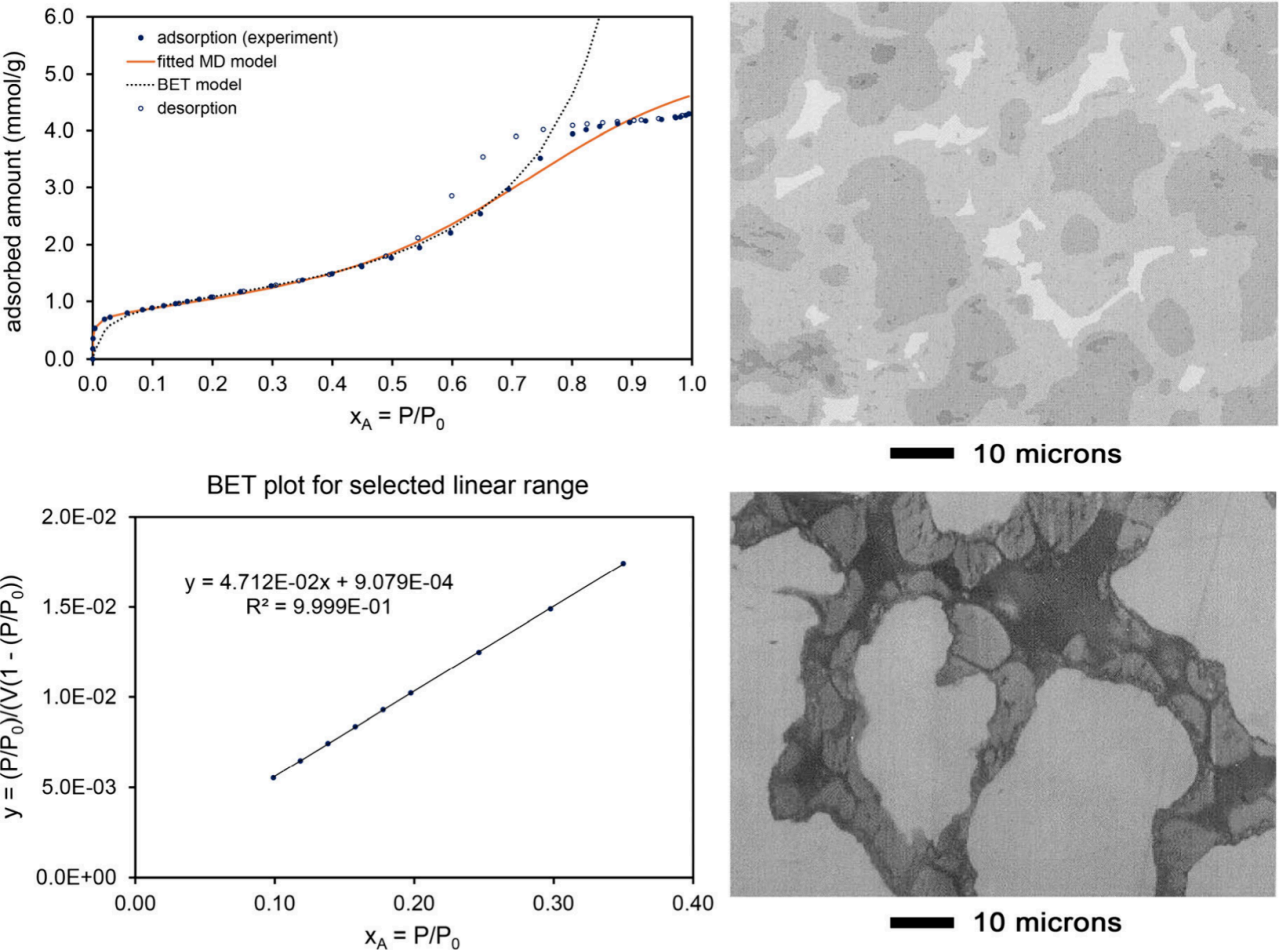
Analysis of N_2_ adsorption at 77.4 K on a porous Al–Ni material after leaching in concentrated aqueous sodium hydroxide. This is a Type IV isotherm with hysteresis due to capillary condensation in mesopores. *Top left:* Comparison of fitted MD and BET model isotherms to the experimental adsorption isotherm. The MD model isotherm fits the experimental data much better than the BET model isotherm. *Bottom left:* Linear regression to determine the BET parameters. *Top right:* Optical micrograph showing the Ni–Al alloy structure before leaching. The three phases are eutectic (lightest), NiAl_3_ (medium), and Ni_2_Al_3_ (darkest). *Bottom right:* Optical micrograph showing the structure of the partially leached material after 16 min leaching in 3 wt % NaOH aqueous solution.

As described in prior literature, small amounts of Fe and/or Cr and/or other metals are often added to the precursor Al–Ni-based alloy as ‘promoters’ to improve the sponge-nickel catalyst’s performance for hydrogenation reactions.^68–74^ Experimentally measured N_2_ adsorption isotherms at 77 K along with MD and BET model isotherm fits are displayed in Figure 7 for a Fe-promoted and Fe–Cr doubly promoted Raney nickel catalysts. These are Type IV isotherms with hysteresis due to capillary condensation in mesopores. As shown in Table 3, the whole isotherm (118 m^2^/g) and BET (126 m^2^/g) surface areas for the Fe–Cr doubly promoted catalyst were significantly higher than for the unpromoted and Fe-promoted catalysts. For all three catalysts, the MD isotherm fitscores (0.980–0.994) were substantially higher than the BET isotherm fitscores (0.621–0.784).

**Figure 7:**
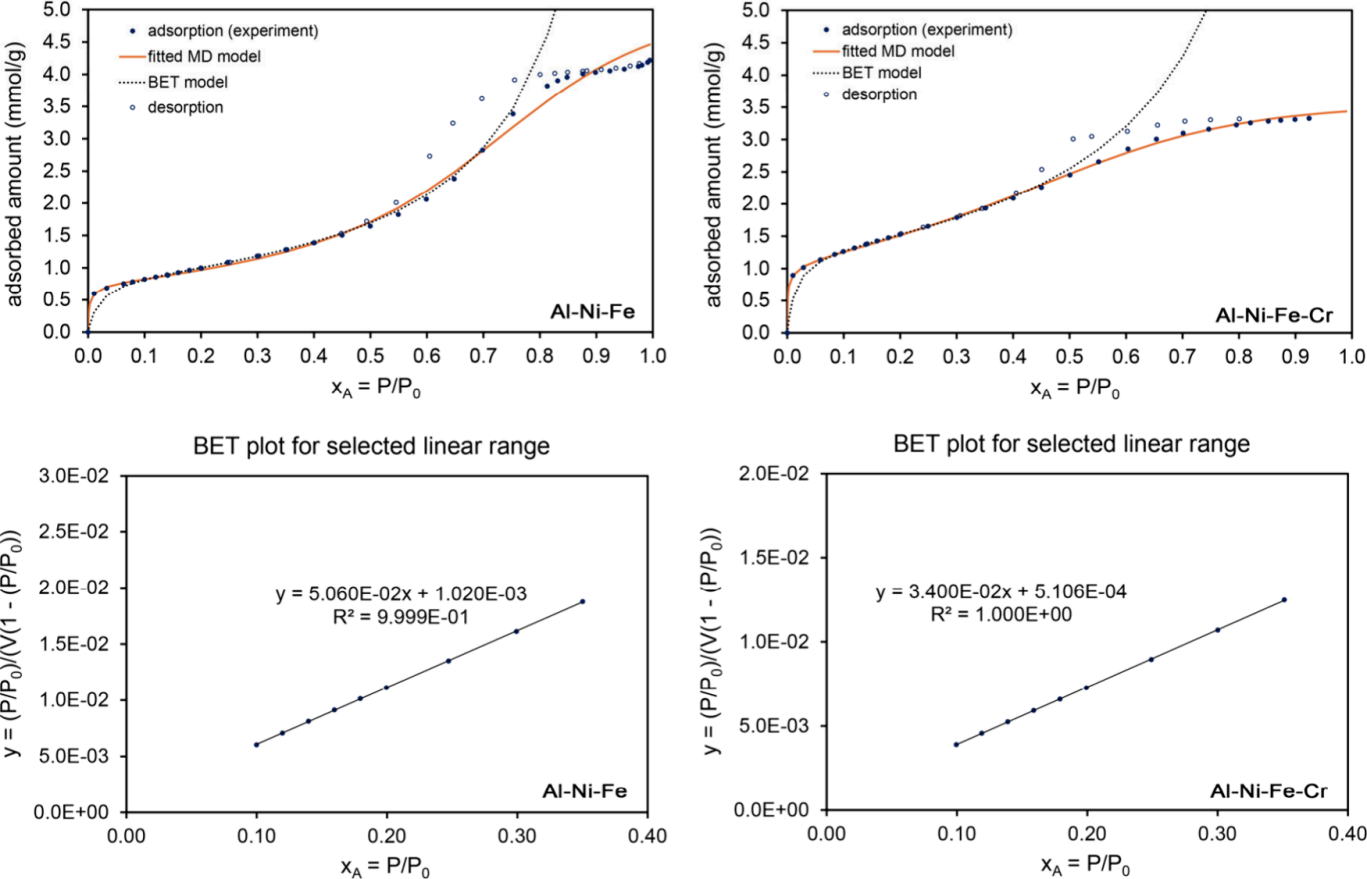
Analysis of N_2_ adsorption at 77 K on porous Al–Ni–Fe (*left panels*) and Al–Ni–Fe–Cr (*right panels*) materials after leaching in concentrated aqueous sodium hydroxide. These are Type IV isotherms with hysteresis due to capillary condensation in mesopores. *Top panels:* Comparison of fitted MD and BET model isotherms to the experimental adsorption isotherm. The MD model isotherm fits the experimental data much better than the BET model isotherm. *Bottom panels:* Linear regression to determine the BET parameters.

A key question is how much of the surface area is occupied by transition-metal elements in reduced metallic form. As explained in prior literature, this can be assessed by measuring carbon monoxide (CO) adsorption isotherms twice on a degassed sample.^75,76^ The first adsorption isotherm measures the total chemisorbed (i.e., strongly chemically bound) and physisorbed (i.e., weakly physically bound) CO. After the first CO adsorption isotherm is measured, the sample is pumped down to remove the weakly physically bound CO while the strongly chemically bound CO is not removed during this pump-down. The second adsorption process then reintroduces CO with the new uptake corresponding to just the weakly physically bound CO. Thus, the amount of strongly chemically bound CO equals the average difference between the first and second adsorption isotherms. As shown in Supporting Information Figure S1, the metallic surface areas measured using this method were ∼40 m^2^/g for both the unpromoted and Fe-promoted catalysts. We previously reported a CO-measured metallic surface area of 50 m^2^/g for the Fe–Cr doubly promoted catalyst.^68^ Comparing these results to the total surface areas listed in Table 3, approximately 40–50% of the surface area for each of these catalysts was in reduced metallic form.

### 3.2 Gas Adsorption in Metal–Organic Frameworks (MOFs) and Zeolite

The top left panel in Figure 8 is the benzene adsorption isotherm on the C_46_H_40_N_4_O_10_Zn_2_ MOF at 323 K. At this temperature, the benzene saturation vapor pressure is P_0_ = 0.357 bar. At saturation, nearly 8 benzene molecules are adsorbed per unit cell. This is a Type V isotherm. The experimental data is from Lan et al.^77^ (original measurement) as digitized in the National Institute of Standards and Technology (NIST) Advanced Research Projects Agency Energy (ARPA-E) Database of Novel and Emerging Adsorbent Materials.^78^ For the MD model, the 95% confidence intervals were (0.92, 1.07) for α, (22, 39) for K_1_, (33.8, 34.3) for K_2_, (5.8, 7.4) for m, and (0.93, 1.14) for N.

**Figure 8:**
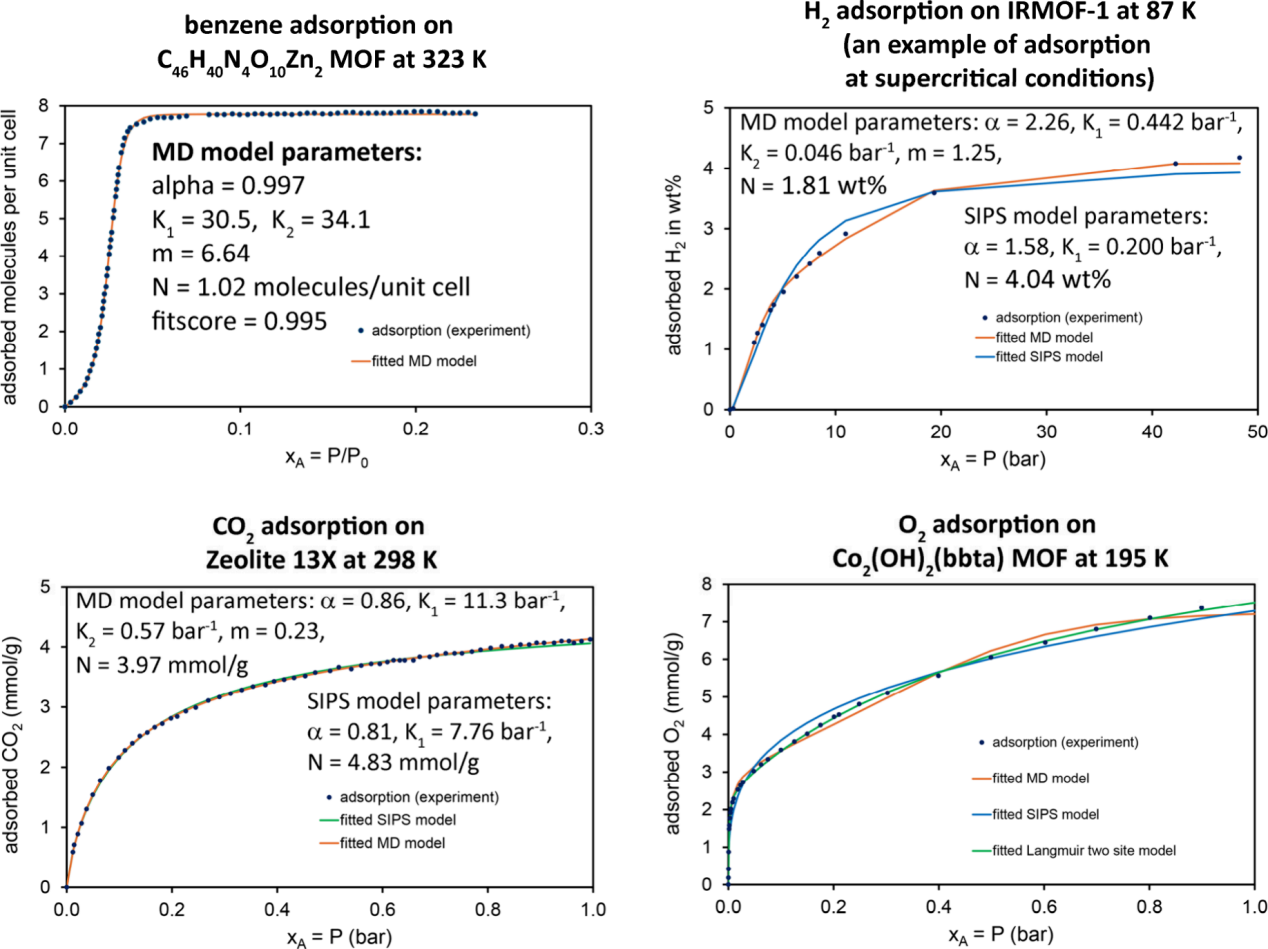
Adsorption of small molecules in MOFs and zeolite. *Top left*: Benzene adsorption on the MOF (C_46_H_40_N_4_O_10_Zn_2_) at 323 K. *Top right:* H_2_ physisorption in IRMOF-1 at 87 K. *Bottom left:* CO_2_ adsorption on Zeolite 13X at 298 K. *Bottom right:* O_2_ adsorption in the Co_2_(OH)_2_(bbta) metal–organic framework at 195 K.

The top right panel in Figure 8 is the H_2_ adsorption isotherm on IRMOF-1 at 87 K. The experimental data is from Panella et al.^79^ as digitized in the NIST ARPA-E Database of Novel and Emerging Adsorbent Materials.^78^ For H_2_, the critical point temperature and pressure are T_c_ = 32.9 K and P_c_ = 12.9 bar.^80^ Consequently, H_2_ does not have a vapor pressure at 87 K. This example demonstrates the MD and Sips models fitted to the adsorption isotherm of a supercritical fluid. Both models fit the experimental data well, with fitscores of 0.990 (MD model) and 0.960 (Sips model). For the MD model, the 95% confidence intervals were (2.16, 2.36) for α, (0.40, 0.48) for K_1_, (0.043, 0.052) for K_2_, (1.09, 1.43) for m, and (1.67, 1.96) for N.

The bottom left panel in Figure 8 is the carbon dioxide adsorption isotherm on zeolite 13X at 298 K. This temperature is just a few degrees below carbon dioxide’s critical temperature of 304 K.^80^ The experimental data is from Maring and Webley^81^ (original measurement) as digitized in the NIST ARPA-E Database of Novel and Emerging Adsorbent Materials.^78^ Both the Sips (fitscore = 0.995) and MD (fitscore = 0.996) isotherms provided excellent fits to the experimental data. For the MD model, the 95% confidence intervals were (0.84, 0.88) for α, (0.4, 12.0) for K_1_, (0.07, 1.13) for K_2_, (0.04, 4.7) for m, and (3.9, 4.4) for N.

As described by Oktawiec et al., O_2_ adsorption on the Co_2_(OH)_2_(bbta) MOF exhibits an unusual adsorption isotherm that contains some features of negative cooperativity.^82^ Specifically, the heat of adsorption (binding affinity) is stronger at lower O_2_ coverages than at higher O_2_ coverages.^82^ This behavior could indicate either that there are multiple types of sites for O_2_ adsorption or else that O_2_ adsorption destabilizes further O_2_ adsorption onto nearby sites.^82^ The bottom right panel in Figure 8 displays Oktawiec et al.’s^82^ experimentally measured O_2_ adsorption isotherm on the Co_2_(OH)_2_(bbta) MOF at 195 K. Table 4 lists optimized parameters and fitscores for the MD, Sips, and two-site Langmuir models. All three models fit the experimental data well, and the two-site Langmuir model provided the best fit. For the MD model, the 95% confidence intervals were (0.6, 0.9) for α, (249, 533) for K_1_, (1.37, 1.94) for K_2_, (1.1, 1.4) for m, and (3.0, 3.6) for N.

**Table 4:** MD, Sips, and Two-Site Langmuir Model Parameters for O_2_ Adsorption onto Co_2_(OH)_2_(bbta) MOF at 195 K

	optimized parameters	fitscore
**MD**	α = 0.729, K_1_ = 387 bar^–1^, K_2_ = 1.65 bar^–1^, m = 1.26, N = 3.21 mmol/g	0.981
**Sips**	α = 0.293, K_1_ = 1.24 × 10^–4^ bar^–1^, N = 109 mmol/g	0.959
**two-site Langmuir**	K_1_ = 927 bar^–1^, K_2_ = 1.60 bar^–1^, N_1_ = 2.45 mmol/g, N_2_ = 8.23 mmol/g	0.993

Several recent journal articles studied the applicability and reproducibility of BET analysis for liquid nitrogen adsorption on MOFs and other materials.^31,32,83–91^ We refer readers to those articles for a detailed discussion of relevant issues associated with applying BET analysis to MOFs.

Table 5 lists MD model parameters, surface areas, and fitscores for liquid N_2_ or liquid Ar adsorption on four MOFs. For comparison, the BET surface areas (computed using the BETSI method^32^) and fitscores are also listed. The BETSI plots for liquid nitrogen adsorption on Al fumarate MOF and IRMOF-1 are presented in Osterrieth et al.^32^ BETSI plots for MOF-IP and Li-SU-102 are presented in the Supporting Information. Examining Table 5, the whole isotherm surface areas (fitted to the MD model) and the BET surface areas were similar to each other for these four materials. As evidenced by the fitscore values, the MD model provided an excellent fit to the experimental isotherm shape, while the BET model provided a poor fit to the experimental isotherm shape.

**Table 5:** MD Model Isotherm Parameters for Liquid N_2_ or Ar Adsorption on Four MOFs^a^

	Al fumarate	IRMOF-1	MOF-IP	Li-SU-102
**adsorbate**	N_2_	N_2_	Ar	N_2_
**temperature (K)**	77	77	87	77
**α**	3.43	1.46	1.62	0.410
	(3.2, 3.7)	(1.42, 1.51)	(1.35, 1.90)	(0.29, 0.55)
**K**_**1**_	1978	191	1875	32808
	(1912, 2044)	(186, 197)	(1562, 2258)	(16930, 65819)
**K**_**2**_	0.846	0.667	0.982	0.667
	(0.747, 0.955)	(0.667, 0.667)	(0.667, 1.50)	(0.667, 0.667)
**m**	0.524	0.103	0.832	0.995
	(0.465, 0.598)	(0.08, 0.12)	(0.68, 1.19)	(0.64, 1.44)
**N** **(mmol/g)**	10.0	35.5	6.99	7.53
	(9.95, 10.05)	(35.2, 35.8)	(6.7, 7.3)	(7.2, 8.1)
**fitscore**	0.975	0.970	0.975	0.973
**surface area** **(m^2^/g)**	976	3460	598	735
	(971, 981)	(3432, 3488)	(571, 623)	(702, 793)
**BET**S.A. **(m^2^/g)**	1007	3252	615	720
**BET fitscore**	0.579	0.571	0.617	0.723

a The MD and BET surface areas and fitscores are also listed. For these systems, the parameters α, K_1_, K_2_, m, and fitscore are unitless. For the MD model, the 95% confidence intervals are shown in parentheses.

This is illustrated graphically in Figure 9, which compares the fitted MD and BET model isotherms to the experimental data. Experimental data for N_2_ adsorption (at 77 K) on Al fumarate and IRMOF-1 is from Osterrieth et al.^32^ as digitized in the NIST ARPA-E Database of Novel and Emerging Adsorbent Materials.^78^ Experimental data for Ar adsorption (at 87 K) on MOF-IP is from Lee et al.^92^ (original measurement) as digitized in the NIST ARPA-E Database.^78^

**Figure 9:**
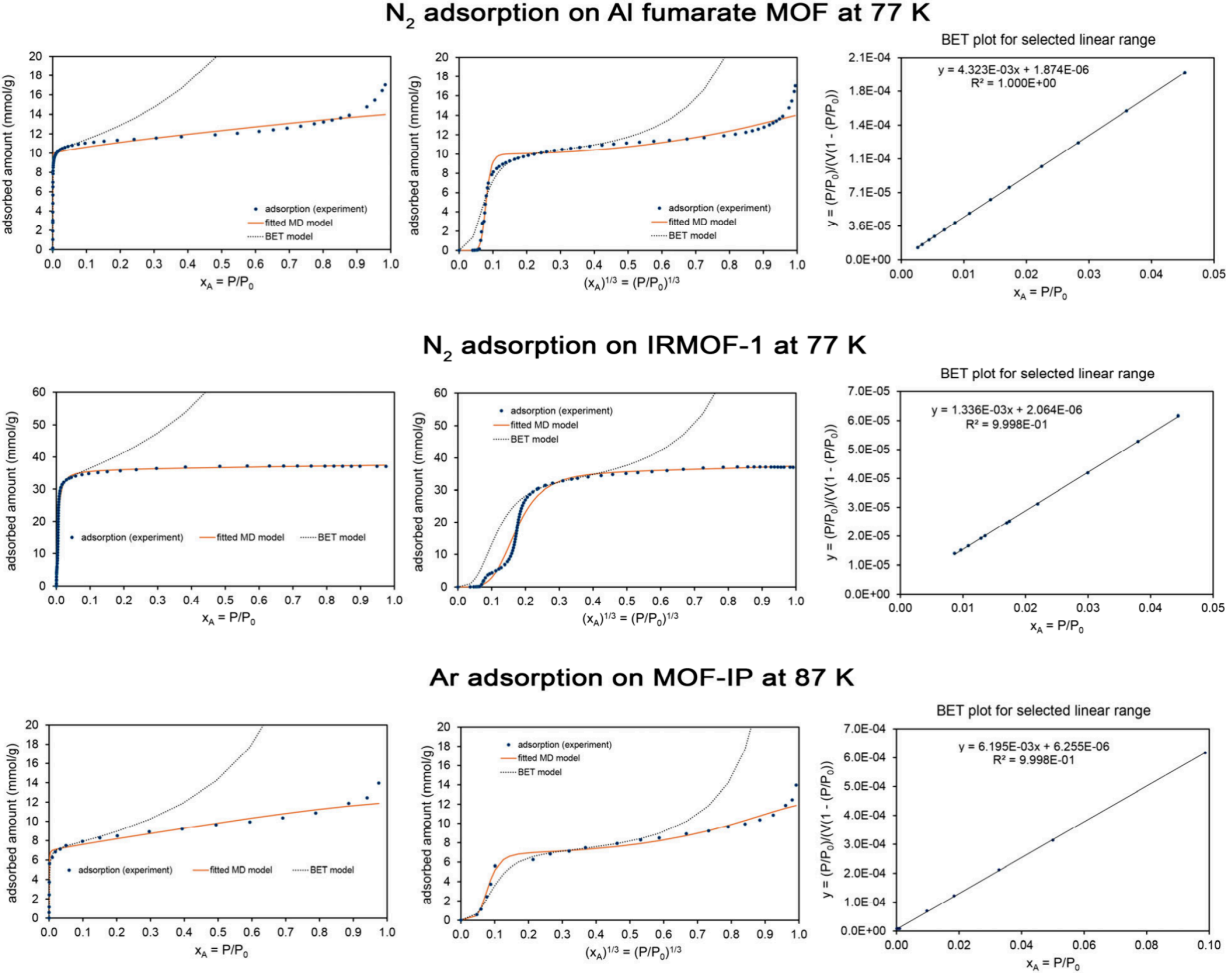
*Top row:* Analysis of N_2_ adsorption at 77 K on Al fumarate MOF. This is a Type IV isotherm. *Middle row:* Analysis of N_2_ adsorption at 77 K on IRMOF-1. This is a Type Ia isotherm*. Bottom row:* Analysis of Ar adsorption at 87 K on MOF-IP. This is a Type IV isotherm. *Left and middle panels:* Comparison of fitted MD and BET model isotherms to experimental adsorption isotherms using P/P_0_ (left panels) and (P/P_0_)^1/3^ (middle panels) on the x-axis. The MD model isotherm fits the experimental data much better than the BET model isotherm. *Right panels:* Linear regression to determine the BET parameters.

Figure 10 is for liquid N_2_ (i.e., at 77 K) and room-temperature (i.e., at 298 K) water adsorption on Li-SU-102. For water adsorption, the offset near zero pressure was removed as indicated by the red arrow in the lower left panel; this offset suggests extremely strong water adsorption on some sites. The fitted MD model parameters for room-temperature water adsorption on Li-SU-102 are listed in Table 6. Comparing the whole isotherm surface area 95% confidence intervals of (702, 793) m^2^/g for N_2_ adsorption to (617, 688) m^2^/g for water adsorption suggests a consensus estimate of ∼700 m^2^/g, with the gap between water and N_2_ confidence intervals possibly due to the uncertainty in cross-sectional area occupied by a water molecule (0.114 nm^2^ per water molecule was used). The lower right panel in Figure 10 shows the BET plot for water adsorption on Li-SU-102 without adjustment. The BET plot for water adsorption after removing the adsorption offset near zero pressure is contained in the Supporting Information zip archive. Both BET plots (i.e., with and without removing the adsorption offset near zero pressure) for water adsorption showed no valid linear range, and this means the BET surface area is not defined for water adsorption on the Li-SU-102 MOF.

**Figure 10:**
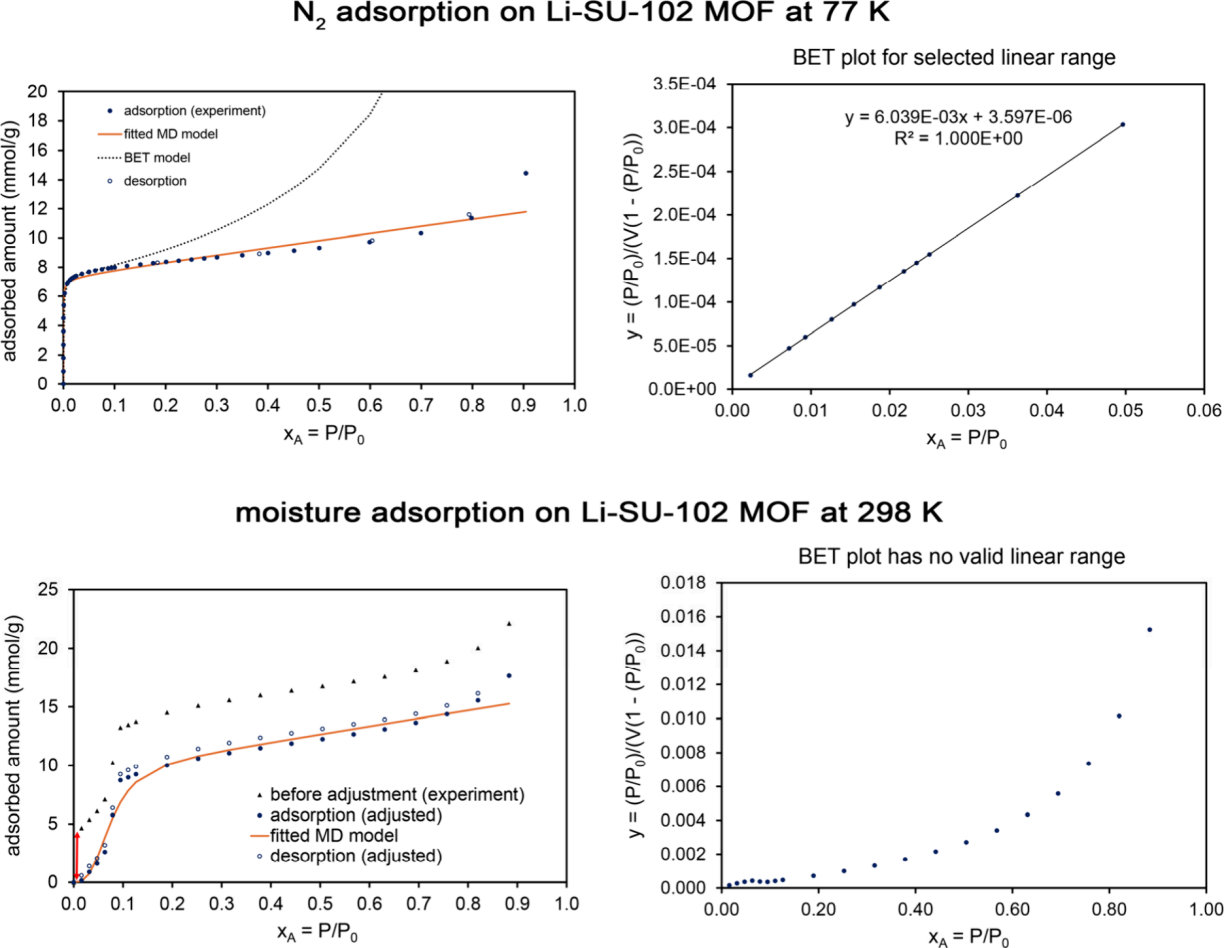
*Top panels*: N_2_ adsorption at 77 K on the Li-SU-102 MOF. *Bottom panels*: H_2_O adsorption at 298 K on the Li-SU-102 MOF. For N_2_ adsorption, the BET plot shows a valid linear range, while, for water adsorption, there is no valid linear range in the BET plot. Both the N_2_ and H_2_O isotherms have no hysteresis between adsorption and desorption. The experimental data is from Oppenheim et al.^93^ For water adsorption, the offset near zero pressure was removed as indicated by the red arrow in the lower left panel.

**Table 6:** Fitted MD Model Parameters for Water Adsorption on Five Materials^a^

	Li-SU-102	potato starch	activated carbon	Ni-DOBDC MOF	UiO-66 MOF
**α**	2.94	0.596	0.533	5.95	3.39
	(2.55, 3.43)	(0.5, 0.7)	(0.479, 0.590)	(5.89, 6.03)	(2.6, 4.1)
**K**_**1**_	13.4	12.8	0.446	50.7	3.53
	(12, 15)	(7, 18)	(0.25, 0.72)	(50.0, 51.3)	(2.9, 4.1)
**K**_**2**_	0.667	0.756	1.50	0.76	1.23
	(0.667, 0.667)	(0.72, 0.79)	(1.5, 1.5)	(0.667, 0.95)	(1.07, 1.5)
**m**	1.10	18.5	4.56	0.34	0.012
	(0.78, 1.5)	(14, 23)	(3.9, 5.2)	(0.24, 0.45)	(0, 0.39)
**N** **(mmol/g)**	9.46	6.10	6.73	23.3	23.6
	(9.0, 10.0)	(5.5, 7.0)	(6.0, 7.7)	(22.9, 23.8)	(18, 24)
**fitscore**	0.957	0.984	0.975	0.988	0.907
**surface area** **(m^2^/g)**	649	419	462	1602	1618
	(617, 688)	(380, 484)	(415, 530)	(1575, 1631)	(1245, 1636)

a In these cases, the parameters α, K_1_, K_2_, m, and fitscore are unitless. The fitscores and whole isotherm surface areas are also listed. For the MD model, the 95% confidence intervals are shown in parentheses.

### 3.3 Water Adsorption in Hydrophobic and Hydrophilic Materials

We studied water adsorption on the five materials shown in Table 6. Table 6 summarizes the MD model parameters, fitscores, and whole isotherm surface areas for these five materials. Some of these materials are hydrophilic, and some are hydrophobic. The water activity, x_A_, was defined as the partial pressure of water divided by the water saturation vapor pressure at the adsorption temperature. For all six materials, water adsorption was performed near room temperature. For each material, the MD model fit the experimental data well.

Water adsorption on various types of agricultural products, foods and foodstuffs, and biomass is commercially important, because these products often need to be dried to enhance their shelf life and storage stability.^52,53,94^ As shown in Figure 11, water adsorption on potato starch has a Type II isotherm corresponding to a large number of adsorption layers. For this material, the surface areas were 419 (MD model) and 315 (BET model) m^2^/g, and the fitscores were 0.984 (MD model) and 0.734 (BET model).

**Figure 11:**
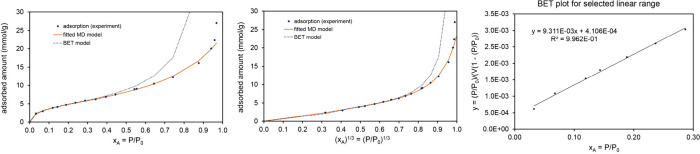
Analysis of water adsorption at room temperature on potato starch. This is a Type II isotherm. *Left and middle panels:* Comparison of fitted MD and BET model isotherms to the experimental adsorption isotherm using P/P_0_ (left panel) and (P/P_0_)^1/3^ (middle panel) on the x-axis. The MD model isotherm fits the experimental data much better than the BET model isotherm. *Right panel:* Linear regression to determine the BET parameters. The experimental data is from Timmermann et al.^52^

For activated carbon (Figure 12), Ni-DOBDC MOF (Figure 13), and UiO-66 MOF (Figure 13), the BET model plot applied to water adsorption had no valid linear range. We confirmed this by analyzing these isotherms using the automated BETSI software procedure.^32^ This stands in contrast to liquid N_2_ adsorption, for which Osterrieth et al. reported BETSI analysis yielded the surface area of 1145 m^2^/g for UiO-66.^32^ Jamil et al. reported liquid N_2_ adsorption BET surface areas of ∼1100 m^2^/g for Ni-DOBDC MOF.^95^ When measuring surface areas of materials, liquid N_2_ adsorption is often preferred over moisture adsorption, because liquid N_2_ adsorption avoids various complications (e.g., hydrophobicity, hydrogen-bonding, solvation, etc.) that may be encountered during moisture adsorption on some materials.

**Figure 12:**
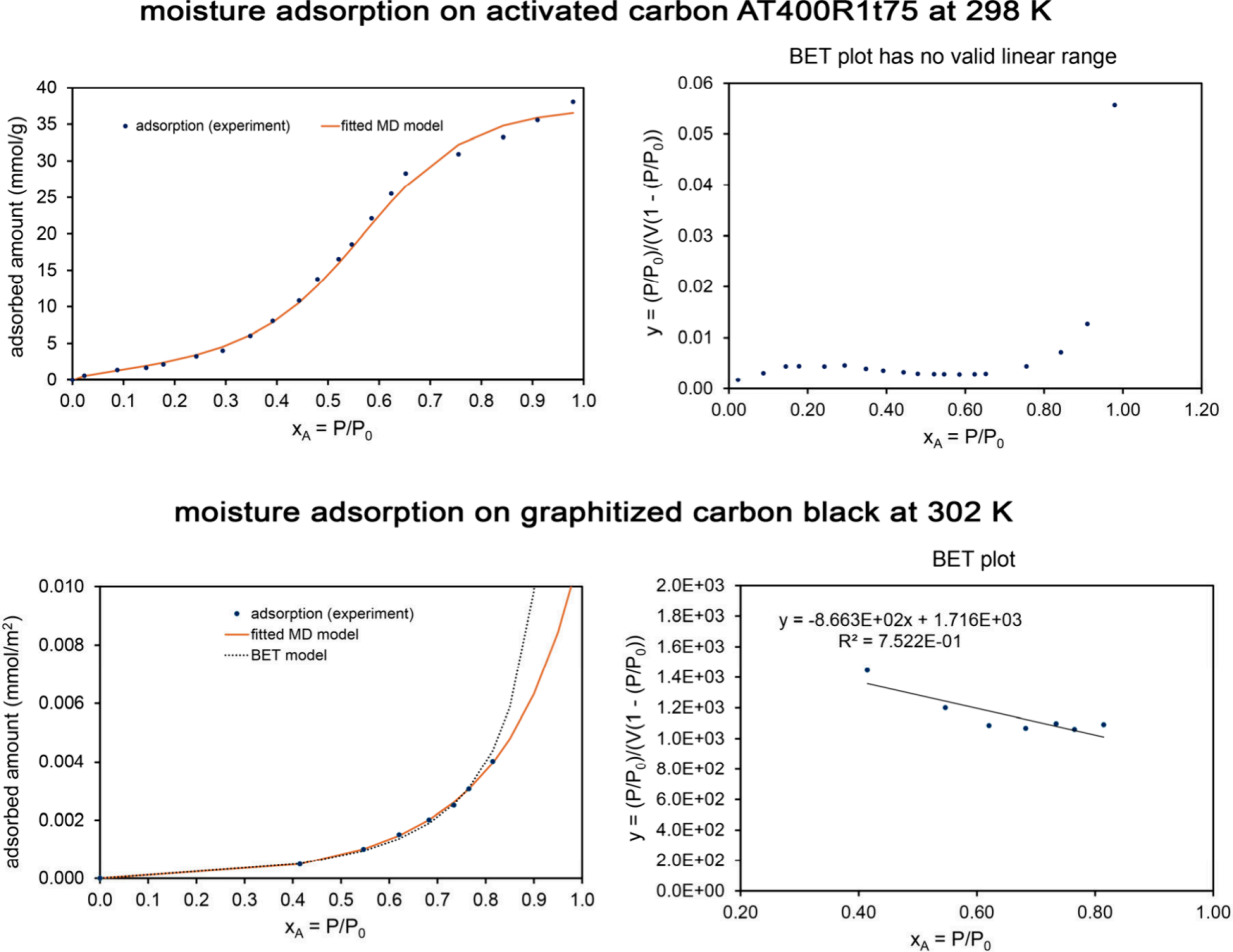
*Top row:* Water adsorption on activated carbon AT400R1t75 at 298 K. This is a hydrophobic material with a Type V isotherm. As shown in the top left panel, the MD model fits the experimental data reasonably well. As shown in the top right panel, there is no valid linear range in the BET plot, and this indicates the BET model cannot fit the experimental data. The experimental data is from de Yuso et al.^97^ (original measurement) as digitized in the NIST ARPA-E Database.^78^ *Bottom row:* Water adsorption on graphitized carbon black at 302 K. The MD model fit the experimental data better than the BET model. The experimental data is from Belyakova et al.^96^ (original measurement) as digitized from the graph in Nguyen et al.^98^

**Figure 13:**
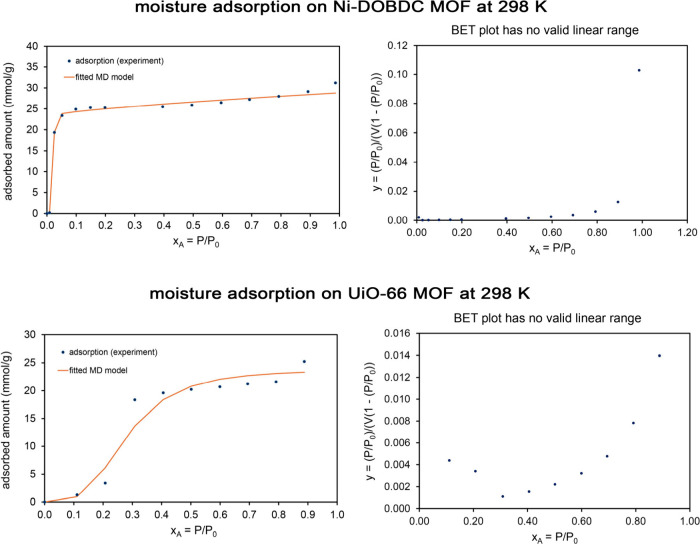
*Top row:* Water adsorption on the Ni-DOBDC metal–organic framework at 298 K. As shown in the left panel, the MD model fits the experimental data reasonably well. As shown in the right panel, there is no valid linear range in the BET plot, and this indicates the BET model cannot fit the experimental data. The experimental data is from Liu et al.^99^ (original measurement) as digitized in the NIST ARPA-E Database of Novel and Emerging Adsorbent Materials.^78^ *Bottom row:* Water adsorption on the UiO-66 metal–organic framework at 298 K. As shown in the left panel, the MD model fits the experimental data reasonably well. As shown in the right panel, there is no valid linear range in the BET plot, and this indicates the BET model cannot fit the experimental data. The experimental data is from Schoenecker et al.^100^ (original measurement) as digitized in the NIST ARPA-E Database of Novel and Emerging Adsorbent Materials.^78^

Belyakova et al. experimentally measured water adsorption on graphitized carbon black with a known surface area.^96^ Using this known surface area and the effective molecular cross-section of 0.114 nm^2^ (refs 31 and 63 ) per water molecule, we computed and fixed the N parameter value in the MD model instead of fitting it. We then optimized the remaining four MD model parameters (α = 1.57, K_1_ = 0.18, K_2_ = 0.74, m = 11.3) for this material using Excel’s GRG solver to maximize the fitscore (0.993). As shown in the bottom row of Figure 12, this is a hydrophobic material with a Type III isotherm. For this material, we optimized the two BET model parameters using Excel’s GRG solver to maximize the fitscore (0.975).

In summary, the MD isotherm is ideally suited for modeling moisture adsorption on various hydrophobic and hydrophilic materials.

### 3.4 Type VI Adsorption Isotherm

Figure 14 shows an example of a Type VI adsorption isotherm. This is an example of step-like layer-by-layer adsorption in which each layer fills up prior to adsorption on the subsequent layer.^101^ The units of adsorption are cm^3^ TPN (Température et Pression Normales) which corresponds to 20 °C and 1 atm. Table 7 compares the fitted MD model parameters using one and two sitegroups. The fitscores were 0.952 (one sitegroup) and 0.983 (two sitegroups, fitted using Excel’s GRG solver). The one sitegroup model did a good job of capturing the average adsorption trend across the entire isotherm; however, it did not capture the clear features individual steps in the isotherm beyond the first step. The two sitegroup model displayed the clear features of both the first and second steps.

**Figure 14:**
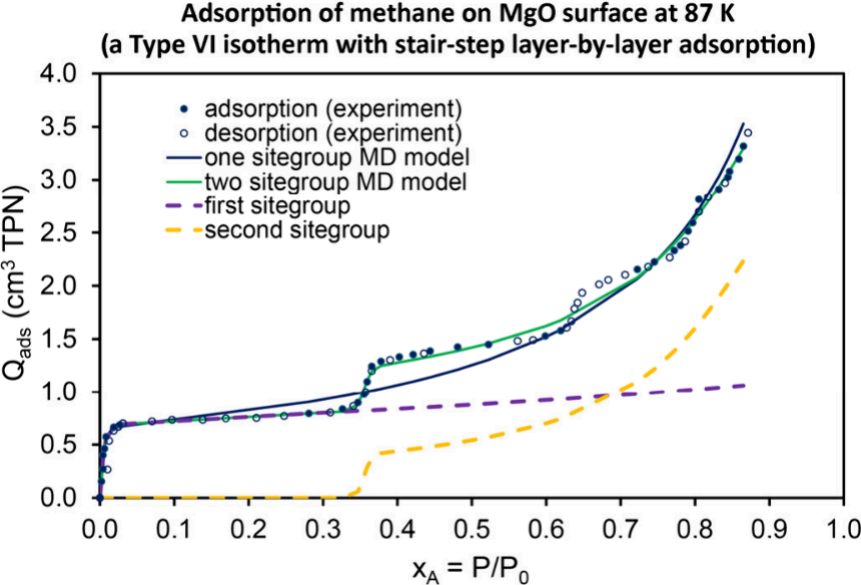
Methane adsorption on a MgO surface at 87 K. The saturation vapor pressure, P_0_, of methane at this temperature is 56 Torr. This is a Type VI isotherm with step-like layer-by-layer adsorption. The adsorption is reversible with no hysteresis between adsorption and desorption. The MD model isotherm using one sitegroup (solid blue line) followed the general trend but did not reproduce the fine structure of the second and third steps. The MD model isotherm using two sitegroups (solid green line) reproduced the fine structure of the first and second steps but only captured the general trend of the third step. The dotted purple and dotted yellow lines show the adsorption isotherm contributions from the first and second sitegroups, respectively. The experimental data is from Gay et al.^101^

**Table 7:** Fitted MD Model Parameters for Methane Adsorption on MgO at 87 K^a^

	one sitegroup	sitegroup 1 of 2	sitegroup 2 of 2
**α**	2.03 (1.80, 2.36)	1.81	74.7
**K**_**1**_	264 (237, 295)	238	2.80
**K**_**2**_	0.938 (0.92, 0.95)	0.384	1.065
**m**	1460^a^	580	16.2
**N** **(cm^3^** **TPN)**	0.665 (0.64, 0.69)	0.709	0.253

a The parameters α, K_1_, K_2_, and m are unitless. For the single sitegroup model, the 95% confidence intervals are shown in parentheses.

b The value of m is ‘large’, and its precise value does not significantly impact the adsorbed amount at any pressure.

### 3.5 Adsorption of Solutes from Liquid-Phase Solutions

Figure 15 studies an example of adsorption of a chemical (specifically, red dye) from aqueous solution onto a form of biomass (specifically, beechwood). As shown in Figure 15, this isotherm exhibits positive cooperativity. As indicated by the fitscore values, both the MD (0.994) and Sips (0.977) models provided a good fit to Muntean et al.’s experimental data.^102^ For the MD model, the 95% confidence intervals were (1.5, 1.7) for α, (0.0106, 0.0166) for K_1_, (0.0096, 0.0123) for K_2_, (0.2, 1.9) for m, and (9.8, 22.6) for N.

**Figure 15:**
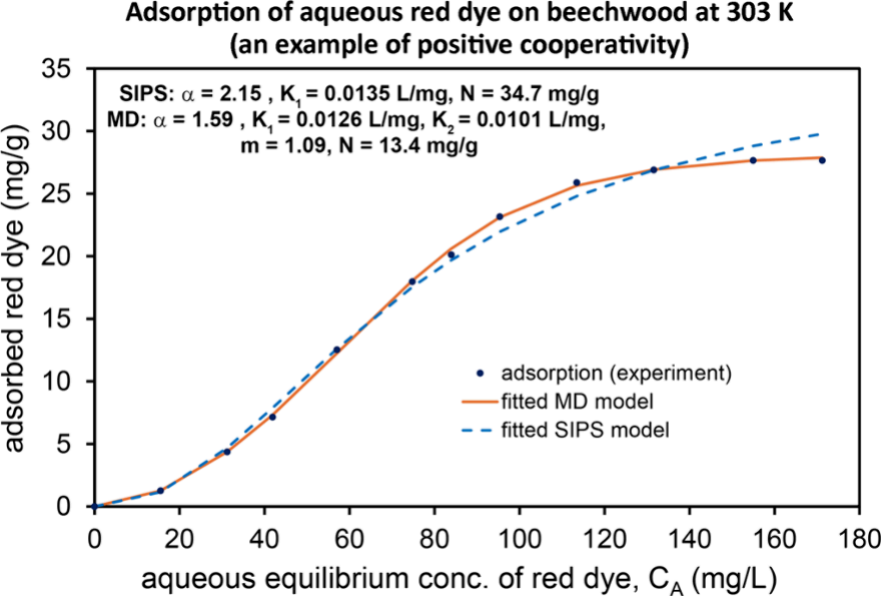
Adsorption of aqueous red dye on beechwood at 303 K. This adsorption isotherm displays positive cooperativity. The experimental data is from Muntean et al.^102^

Adsorption of proteins onto surfaces is an important application of the MD isotherm for the following reasons. Protein adsorption can exhibit both positive and negative cooperativity.^103^ Positive cooperativity means that adsorption of some protein molecules makes it more favorable for additional protein molecules to adsorb, and this corresponds to attractive protein–protein lateral interactions.^103^ Negative cooperativity means that adsorption of some protein molecules makes it less favorable for additional protein molecules to adsorb, and this corresponds to negative protein–protein lateral interactions.^103^ Negative cooperativity can be caused by initial protein molecules adsorbing in an ‘unfolded’ configuration that takes up more surface space than the native protein configuration.^103^ When additional protein molecules adsorb, this suppresses the unfolded configuration which makes the adsorption energy less favorable.^103^ In addition to lateral protein–protein interactions, multilayer stacking of protein molecules can also occur.^103^ The MD isotherm is ideally suited for modeling protein adsorption onto surfaces, because it includes these basic types of interactions.

Figure 16 and Table 8 compare fits of the MD, Sips, and two-site Langmuir models for three proteins (i.e., bovine serum albumin (BSA), the monoclonal antibody mAb2, and alpha-Lactalbumin (LAC)) adsorbed onto two different hydrophobic interaction chromatography resins (Butyl Sepharose (BS) and TOYOPEARL Butyl-650C (TP)). The experimental data from Muca et al. includes adsorption isotherms for BSA and mAb2 adsorbed onto both resins and LAC adsorbed onto the BS resin only.^103^ As shown in Figure 16 and Table 8, all three model isotherms (i.e., MD, Sips, and two-site Langmuir) provided reasonably good fits to the experimental data. As shown in Table 8, the MD isotherm always provides a fitscore greater than or equal to that of the Sips isotherm, because the Sips isotherm is a special case of the MD isotherm. As shown in Table 8, the fitscore for the MD isotherm was usually but not always greater than that of the two-site Langmuir isotherm. Notably, some of the fitted parameters for the two-site Langmuir isotherm were unusually high or unusually low. For BSA on TP and mAb2 on BS, the K_2_ values were around 5 × 10^–5^ mL/nanomol while the N_2_ values were greater than 10^4^ nanomol/mL. Since the largest experimentally measured uptakes in Figure 16 were on the order of 60 to 600 nanomol/mL, those fitted parameter values for the two-site Langmuir model seem to be too extreme. The MD and Sips models having one sitegroup did not exhibit such huge extremes of parameter values.

**Figure 16:**
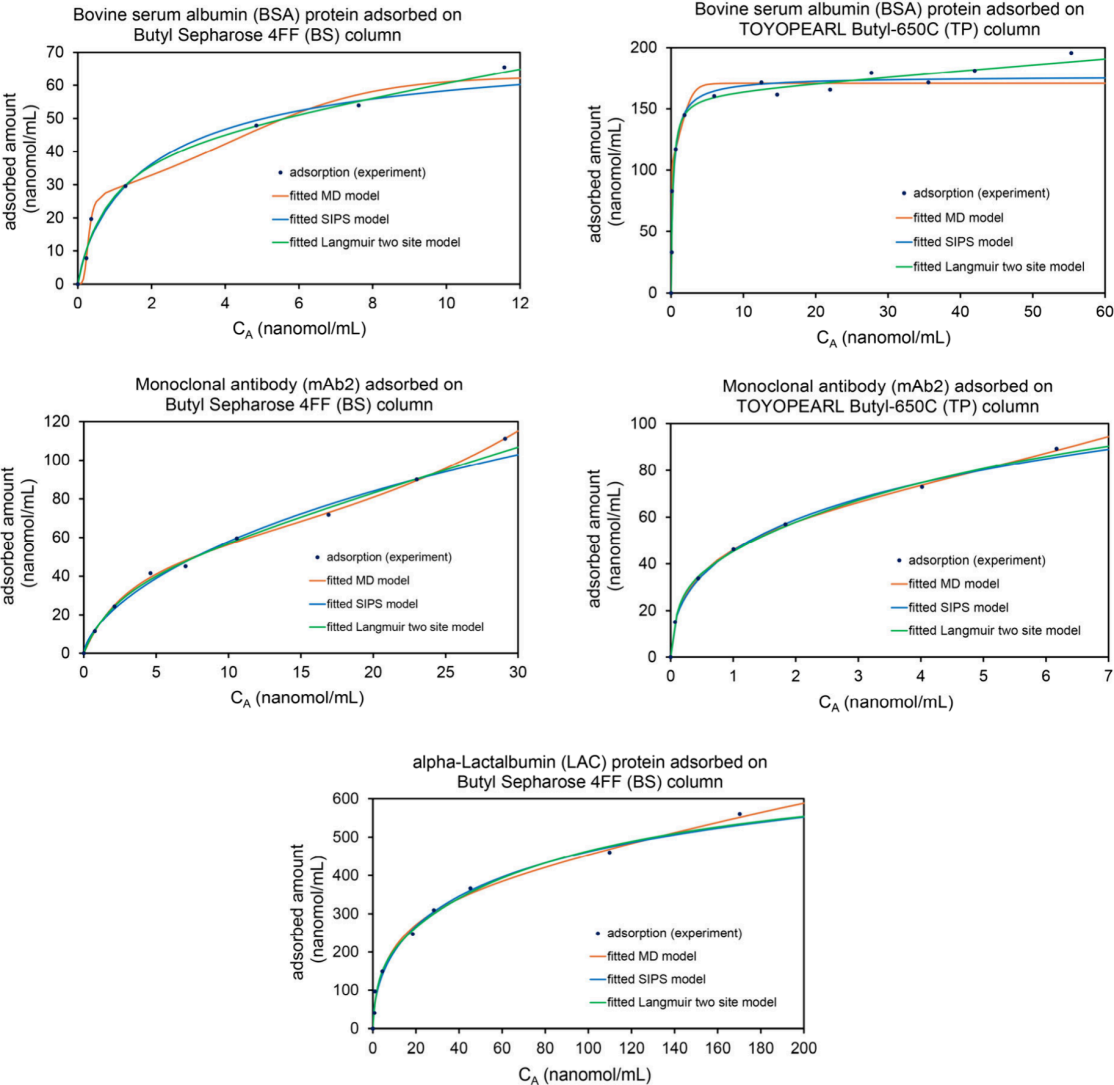
Adsorption of proteins onto hydrophobic interaction chromatography resins. The MD, Sips, and two-site Langmuir models provided reasonably good fits to the experimental data. The experimental data is from Muca et al.^103^

**Table 8:** MD, Sips, and Two-Site Langmuir Model Isotherm Parameters and Fitscores for Protein Adsorption onto Hydrophobic Interaction Chromatography Resins^a^

	BSA on BS	BSA on TP	mAb2 on BS	mAb2 on TP	LAC on BS
*MD Model*					
**α**	4.30	8.47	1.05	0.605	0.710
	(3.9, 4.7)	(6.6, 8.7)	(0.92, 1.23)	(0.55, 0.66)	(0.4, 1.1)
**K**_**1**_	3.50	9.11	0.337	1.19	0.0971
	(3.3, 3.6)	(8.5, 9.2)	(0.2, 0.5)	(0.6, 1.9)	(0.0003, 0.2)
**K**_**2**_	0.130	0.343	0.0174	0.0391	0.00323
	(0.09, 0.18)	(0.04, 0.39)	(0.015, 0.025)	(0.025, 0.050)	(0.0015, 0.026)
**m**	1.49	0.702	5.53	5.15^a^	0.835
	(1.3, 1.7)	(0.46, 0.76)	(2, 7)		(0.00, 144)
**N**	25.2	100.3	57.3	82.3	405.5
	(23, 28)	(98, 130)	(46, 66)	(70, 105)	(278, 2202)
**fitscore**	0.981	0.975	0.985	0.995	0.962
*Sips Model*					
**α**	0.824	0.870	0.654	0.461	0.540
**K**_**1**_	0.463	3.262	0.00674	0.0367	0.00842
**N**	74.83	176.8	395.4	255.6	968.5
**fitscore**	0.948	0.957	0.970	0.989	0.960
*Two-Site Langmuir Model*					
**K**_**1**_	1.191	4.190	0.392	9.795	0.488
**N**_**1**_	45.31	162.5	43.52	34.21	183.3
**K**_**2**_	0.000439	4.528 × 10^–5^	5.166 × 10^–5^	0.146	0.0111
**N**_**2**_	4297	1.065 × 10^4^	4.307 × 10^4^	112.0	540.6
**fitscore**	0.953	0.960	0.979	0.989	0.963

a BSA = bovine serum albumin, mAb2 = monoclonal antibody, LAC = alpha-Lactalbumin, BS = Butyl Sepharose 4FF column, TP = TOYOPEARL Butyl-650C column. The units of K_1_ and K_2_ are mL/nanomol. The units of N, N_1_, and N_2_ are nanomol/mL. The other parameters are unitless. For the MD model, the 95% confidence intervals are shown in parentheses.

b In this case, the confidence interval on m is unbounded, because some of the bootstrapped m values led to unconfined (i.e., m approaches infinite) multilayer adsorption.

## 4 Conclusions

This article introduced a new adsorption model isotherm having extremely wide versatility that applies to all six IUPAC-classification types of physisorption isotherms. A key application of this model isotherm is to extract whole-isotherm surface areas of porous materials from the experimentally measured adsorption experiments over the entire pressure range. This model isotherm is well-suited for modeling: (a) liquid N_2_ (at 77K) and liquid Ar (at 87 K) physisorption, (b) moisture adsorption onto various hydrophobic and hydrophilic materials, (c) protein adsorption onto surfaces, (d) adsorption of various gases (e.g., H_2_, CH_4_, CO_2_, O_2_, benzene, etc.) onto porous materials such as metal–organic frameworks and zeolites, (e) adsorption of solutes from liquid-phase solutions onto porous solids. Our model isotherm is formulated to apply to fluids both below and above the critical temperature and critical pressure.

The main utility of our MD model isotherm is that it provides a mathematical unification of several existing isotherm families without introducing a radically different adsorption mechanism. Section 2.2 above explained relationships between our model isotherm and several model isotherms from prior literature. Some notable limiting cases of our model isotherm include the following. In the extremely low coverage limit, the Freundlich isotherm is recovered, and this reduces to the Henry’s isotherm when α=1. When adsorption is single-layer (i.e., m = 0) at any coverage, the Sips isotherm is recovered, and this reduces to the Langmuir isotherm when α=1. The BET isotherm is recovered from our MD isotherm by choosing m → ∞, K_2_ = 1, α = 1, and x_A_ = p/p_0_, where p_0_ is the saturation pressure at the adsorption temperature. Anderson’s isotherm is a special case of the MD isotherm corresponding to the situation for which m → ∞, 0 < K_2_ < 1, α = 1, and x_A_ = p/p_0_. The GAB isotherm, which is often used to model moisture adsorption on materials, is similarly recovered when m → ∞, 0 < K_2_ < 1, α = 1, and x_A_ is the water activity.

A model adsorption isotherm should fulfill a sort of ‘Goldilocks condition’ (aka ‘happy medium’ condition) in which there are neither too many nor too few adjustable parameters. When a model adsorption isotherm has too few adjustable parameters, its flexibility is too limited so it cannot accurately reproduce a wide range of experimentally measured adsorption isotherm shapes. When a model adsorption isotherm has too many adjustable parameters, this causes an overfitting problem in which some of the parameter values are poorly determined due to multicollinearity issues. In this case, too many values of the adjustable parameters can reproduce nearly the same isotherm shape.

When using a single sitegroup, our new model adsorption isotherm has five fitted parameters: α, K_1_, K_2_, m, and N. This usually provides a ‘Goldilocks condition’ in which there are neither too many nor too few adjustable parameters.

However, there are situations in which these five adjustable parameters are more than needed. To avoid overfitting, it is preferred to fix the value of one or more parameters and adjust the remaining ones. Examples of this situation included: (a) water adsorption at on graphitized carbon black (see Figure 12), and (b) butylamine adsorption from liquid solution onto sponge nickel catalysts (see Supporting Information Section S2). For (b), the Langmuir model used corresponds to setting the MD parameters m = 0 and α = 1. The Sips isotherm corresponds to setting the MD parameter m = 0.

On the other hand, there are also situations in which more than five adjustable parameters are needed to fit the shape of the adsorption isotherm. This situation can occur for Type VI isotherms with step-like layer-by-layer adsorption such as methane adsorption on a MgO surface at 87 K as shown in Figure 14. In such cases, using more than one sitegroup provides additional flexibility to fit the MD model to the experimentally measured isotherm. Caution must be exercised to avoid overfitting.

Section 2.4 explains the recommended procedure for optimizing the MD model parameters. Specifically, the optimized parameter values maximize the fitscore. This fitscore was defined to provide a robust, stable, and balanced goodness-of-fit measure. Its value is always between 0 and 1, where a value of 1 denotes perfect fit. Section 2.5 explained the equation for computing the whole isotherm surface area from N. Section 2.6 presented the algorithm for computing 95% confidence intervals on the optimized parameter values and whole isotherm surface area. The Supporting Information contains a Matlab code implementing this fitting algorithm along with usage instructions and worked examples.

In this article, we applied our new model isotherm to the following specific examples: (a) liquid N_2_ adsorption onto several porous nickel alloy sponges and metal–organic frameworks (MOFs), (b) adsorption of several gas molecules into MOFs, (c) moisture (i.e., water) adsorption onto various kinds of hydrophilic and hydrophobic materials including activated carbons, MOFs, and an agricultural product, (d) five examples of protein adsorption onto hydrophobic interaction chromatography resins, (e) CO_2_ adsorption on Zeolite 13X, (f) butylamine adsorption from methanol–water solvent solution onto sponge nickel catalysts, (g) adsorption of red dye from aqueous phase onto beechwood, (h) step-like layer-by-layer adsorption of methane onto a MgO surface at 87 K. These examples spanned: (i) each of the six IUPAC physisorption isotherm types, (ii) positive cooperative, negative cooperative, and noncooperative adsorption, (iii) single-layer and multilayer adsorption, (iv) adsorption with and without capillary condensation, and (v) adsorption modeled by a single sitegroup and by multiple sitegroups. Please see Figure 17 for a summary. These results demonstrate that our MD isotherm is a good general-purpose model for physical adsorption under diverse conditions on diverse materials.

**Figure 17:**
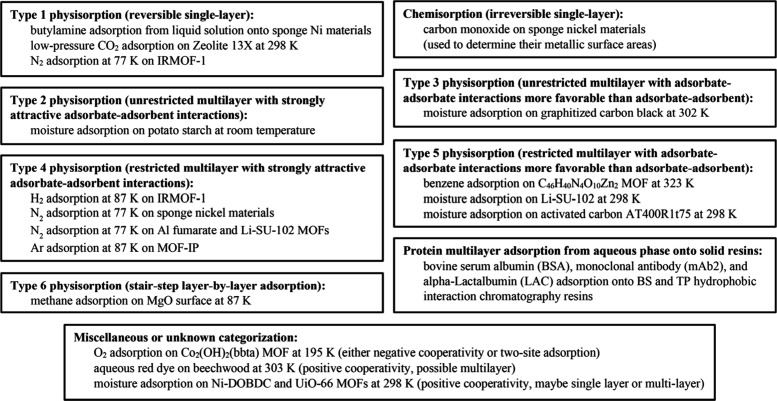
Categorization of the various adsorption isotherms studied in this article. The MD model isotherm was fitted to all of these except the carbon monoxide chemisorption and the liquid-solution butylamine adsorption onto sponge nickel materials. The butylamine adsorption required only the Langmuir model isotherm, which is a limiting case of the MD model isotherm. The carbon monoxide chemisorption was irreversible and used to determine the metallic surface areas. As shown in the bottom box, a few of the adsorption isotherms were classified as ‘miscellaneous or unknown categorization’.

For some of the examples described above, we compared whole-isotherm surface areas (obtained by fitting the MD model to the entire experimentally measured pressure range) to BET surface areas (obtained by fitting the BET model to a small pressure range). Examples in which the MD whole-isotherm and BET surface areas were similar included: liquid N_2_ adsorption onto sponge nickel materials, liquid Ar adsorption onto MOF-IP, water adsorption onto potato starch, and liquid N_2_ adsorption onto the MOFs Al fumarate, IRMOF-1, and Li-SU-102. For some gas adsorption isotherms, the MD whole-isotherm surface area exists but a BET surface area does not exist, because there is no linear region on the BET plot. Examples of this situation included: water adsorption onto activated carbon (AT400R1t75) and the MOFs Ni-DOBDC, UiO-66, and Li-SU-102. Accordingly, the whole isotherm surface area is more versatile than the BET surface area.

What are the limitations of the MD model isotherm? From a conceptual and theoretical perspective, the main limitation is that the MD model assumes each adsorption step except the first and last steps has the same adsorption constant K_2_. The first adsorption step has the adsorption constant K_1_. If m > 0, then the effective adsorption constant for the last adsorption step is increased relative to K_2_ via the condensation term. For example, in a five-step adsorption process, the MD model isotherm assumes that K_2_ = K_3_ = K_4_ < K_5_ with K_1_ being independent. Another limitation is that the MD model isotherm assumes a particular form for the condensation term (see eqns [Disp-formula eq10] and 11). This form is usually a reasonable approximation, but it is not exact. The MD model isotherm does account for different areas in different adsorption layers, but it assumes these areas follow a geometric progression across adsorption steps except the first and last steps. Correctly, the MD model isotherm does not assume the first and second adsorption layers have the same areas. This is a rather subtle consequence that arises due to the folding of the area fraction f into the effective K_2_, K_1_, and m values as described in Sections 2.1 and 2.2 above.

In summary, the MD model isotherm and MD whole-isotherm surface area should find broad applications for modeling physical adsorption (aka physisorption) from gases, liquid solutions, and supercritical fluids onto porous and nonporous solids.

## Supplementary Material





## Data Availability

The data supporting this article have been included as part of the Supporting Information.

## Notes

Procedure used to measure N_2_ adsorption at 77 K on sponge nickel materials; scientific description of sponge nickel materials; Brunauer–Emmett–Teller Surface Identification (BETSI) plots; derivation of the form of condensation term; proof that Q_A_ is non-negative and monotonically increasing; description of how to use the enclosed programs to compute MD model optimized parameters, surface area, and confidence intervals (PDF)
Files containing experimental adsorption data, fitted adsorption model parameters and fitscore values, 95% confidence intervals, and (where applicable) computed surface areas for each material studied in this article; Matlab codes for optimizing the MD model parameters and computing 95% confidence intervals (ZIP)

## ‡

Deceased August 17, 2021.
