# Supplementary material for: A General-Purpose Model Adsorption Isotherm and Whole Isotherm Surface Area Measurement Method
Source: ACS Omega. 2026 Mar 31;11(14):22171–97. doi: 10.1021/acsomega.5c13463 (PMC13084505; doi:10.1021/acsomega.5c13463)
Supplement: Supplementary file 1 [file ao5c13463_si_001.pdf]

Electronic Supporting Information for

A General-Purpose Model Adsorption Isotherm and Whole Isotherm Surface Area Measurement Method

Thomas A. Manz and W. Nicholas Delgass
*corresponding author email: thomasamanz@gmail.com

Contents

Procedure used to measure N_2 adsorption at 77 K on sponge nickel materials

Scientific description of sponge nickel materials

Brunauer-Emmett-Teller Surface Identification (BETSI) plots

Derivation of the form of condensation term

Proof that Q_A is non-negative and monotonically increasing

How to use the enclosed programs to compute MD model optimized parameters, surface area, and confidence intervals

S1. Procedure used to measure N_2 adsorption at 77 K on sponge nickel materials

After leaching, the material was washed with deionized water several times. The general procedure used to measure N_2 adsorption at 77 K was:

1. Approximately 5 mL deionized water were added into a small flask containing the porous material powder to form a slurry. The sample was weighed under water. Approximately 0.5–2.0 grams of sample were used.
2. Using a glass pipette, the sample was transferred into the glass sample holder. This glass sample holder was 28.5 cm in length. The top portion of the holder was a glass tube with an inside diameter of 1 cm and an outside diameter of 1.2 cm. The bottom portion of the tube was spherical in shape with a diameter of 2.8 cm. During this transfer, the tip of the pipette was kept covered in water to minimize exposure of the sample to air.
3. The sample was rinsed with deionized (DI) water. Next, a pipette was used to remove as much of the water as possible.
4. The glass sample holder containing the sample was attached to a Micromeritics ASAP 2000 unit (aka 'adsorption apparatus') using an o-ring connector.
5. The tube connecting the sample holder to the turbo pump passed through a dewar flask filled with liquid nitrogen. This liquid nitrogen trap condensed gases and prevented them from entering the turbo pump.
6. A small heating sleeve was placed around the sample and the temperature set to 120 °C. The sample was outgassed at 120 °C until the pressure fell below 0.006 torr.
7. Usually, it was necessary to de-ice the liquid nitrogen trap during outgassing. The valve above the sample was closed and the rate of pressure drop in the manifold monitored. If the pressure dropped immediately to a very low value (< 0.001 torr), the trap was not blocked by ice. If the pressure fell slowly, ice was blocking the trap. To de-ice the trap, the adsorption unit was shut down and the liquid nitrogen dewar removed. To prevent the sample from being exposed to air, the valve above the sample was kept closed during de-icing. The glass trap was removed from the unit and heated under water to melt the ice. Ice was also removed from the tube attached to the adsorption unit which inserted into the trap. All components were then dried and put back together and the unit was turned on.
8. After the pressure fell below the desired value (0.006 torr), the heating sleeve was removed and a closed tube used to measure the saturation pressure of liquid nitrogen was placed next to the sample holder. A dewar filled with liquid nitrogen was placed below the sample, and the analyzer program was begun.
9. The analyzer program evacuated the sample for an additional pre-set time. After this time, it raised the liquid nitrogen dewar to submerse the sample. Time was given for the pressure and temperature to equilibrate.
10. When the program was ready to begin analysis, it introduced small measured volumes of nitrogen into the manifold and measured the pressure. The valve to the sample was opened and the pressure remeasured. The volume of adsorbed gas was calculated based on the pressure drop and the volume of the sample holder.
11. The volume of adsorbed gas versus pressure gave the nitrogen adsorption isotherm. After the adsorption measurements were completed, the pressure was decreased by fixed intervals and the desorption isotherm was measured. Hysteresis between these two isotherms occurred due to capillary condensation.

After measurements were completed, the sample tube was backfilled with nitrogen and removed.

S2. Scientific description of sponge nickel materials

We prepared several sponge nickel catalysts by leaching Mn-Ni and Al-Ni alloys. Synthesis and characterization details of the Mn-Ni alloys and leached materials are described refs [S1, 2].

The Al-Ni alloys were made by W.R. Grace & Co. Sponge nickel catalysts and Al-Ni alloys marketed by W.R. Grace & Co. are called Raney nickel. Raney nickel is a registered trademark of W.R. Grace & Co. This catalyst type was originally developed and patented by Murray Raney for hydrogenation reactions.[S3-8] In 1963, Raney's company was bought by W.R. Grace. In 2022, the Raney brand of nickel catalysts received the National Historic Chemical Landmark Award from the American Chemical Society.

The Al-Ni alloys were leached in concentrated sodium hydroxide.[S9-11] In basic solutions, some of the hydroxide ions are typically complexed with the leached cation. Two of the aqueous products of leaching aluminum in basic sodium hydroxide solutions are $Al(OH)_3$ and $[Al(OH)_4]^-$:

$$\underset{\text{(solid)}}{NiAl_x} + \underset{\text{(aqueous)}}{3(x-y)H_2O} + \underset{\text{(aqueous)}}{(x-y)NaOH} \rightarrow \underset{\text{(solid)}}{NiAl_y} + \underset{\text{(aqueous)}}{(x-y)\left[Al(OH)_4\right]^{-1}} + \underset{\text{(gas)}}{1.5(x-y)H_2} + \underset{\text{(aqueous)}}{(x-y)Na^{+1}} \quad \text{(S1)}$$

$$\underset{\text{(solid)}}{Al(OH)_3} + \underset{\text{(aqueous)}}{NaOH} \leftrightarrow \underset{\text{(aqueous)}}{\left[Al(OH)_4\right]^{-1}} + \underset{\text{(aqueous)}}{Na^{+1}} \quad \text{(S2)}$$

The ability of Al^{+3} to complex in basic solutions as $[Al(OH)_4]^-$ instead of as $Al(OH)_3$ makes it soluble in basic solutions, and this solubility is an extremely important factor during the leaching process. However, a portion of the $Al(OH)_3$ remains undissolved (as a precipitate) in the catalyst's pores. Some of the aluminum atoms that remain bonded to nickel atoms in the leached catalyst are in the metallic (i.e., zero) oxidation state.[S9,16]

Metallic oxides which can undergo acid-base reactions (in which hydrogen ions are exchanged between chemical species) to dissolve in both acidic and basic solutions are called 'amphoteric'. Aluminum oxide is amphoteric, which means that it is soluble in both strong acids and bases.

Manganese(II) oxide is not amphoteric and will not dissolve in strong bases. Leaching manganese alloys in alkaline solutions is not desirable, because the insoluble manganese(II) hydroxide formed will plug the catalyst's pores. For example, Fasman et al. found that an Al-Ni-Mn (50 wt.% Al, 25% Ni, 25% Mn) alloy when leached in basic solution produced a catalyst in which $Mn(OH)_2$ and other oxidized forms of Mn blocked the reduced Ni metal surface.[S12] An acidic leach is needed for this element. To prevent nickel dissolution, the pH must be above 3. The relevant half reactions are:

$$\underset{\text{(solid)}}{Mn} + \underset{\text{(aqueous)}}{2H^+} \rightarrow \underset{\text{(aqueous)}}{Mn^{2+}} + \underset{\text{(gas)}}{H_2} \quad E^0 = 1.18 \text{ volt} \quad E^{pH3} = 0.92 \text{ volt} \quad \text{(S3)}$$

$$\underset{\text{(solid)}}{Ni} + \underset{\text{(aqueous)}}{2H^+} \rightarrow \underset{\text{(aqueous)}}{Ni^{2+}} + \underset{\text{(gas)}}{H_2} \quad E^0 = 0.26 \text{ volt} \quad E^{pH3} = 0.00 \text{ volt} \quad \text{(S4)}$$

The potential for manganese oxidation to Mn^{2+} or $Mn(OH)_2$ is positive (i.e., favorable) at all pH's. Further oxidation to +3 or higher oxidation states is not favored. Voltages for these half-reactions were computed from the standard potentials reported by Bard et al.[S13]

A key question is how much of the surface area is occupied by transition-metal elements in reduced metallic form. As explained in prior literature, this can be assessed by measuring carbon

monoxide (CO) adsorption isotherms twice on a degassed sample.[S14, 15] The first adsorption isotherm measures the total chemisorbed (i.e., strongly chemically bound) and physisorbed (i.e., weakly physically bound) CO. After the first CO adsorption isotherm is measured, the sample is pumped down to remove the weakly physically bound CO while the strongly chemically bound CO is not removed during this pump-down. The second adsorption process then reintroduces CO with the new uptake corresponding to just the weakly physically bound CO. Thus, the amount of strongly chemically bound CO equals the average difference between the first and second adsorption isotherms.

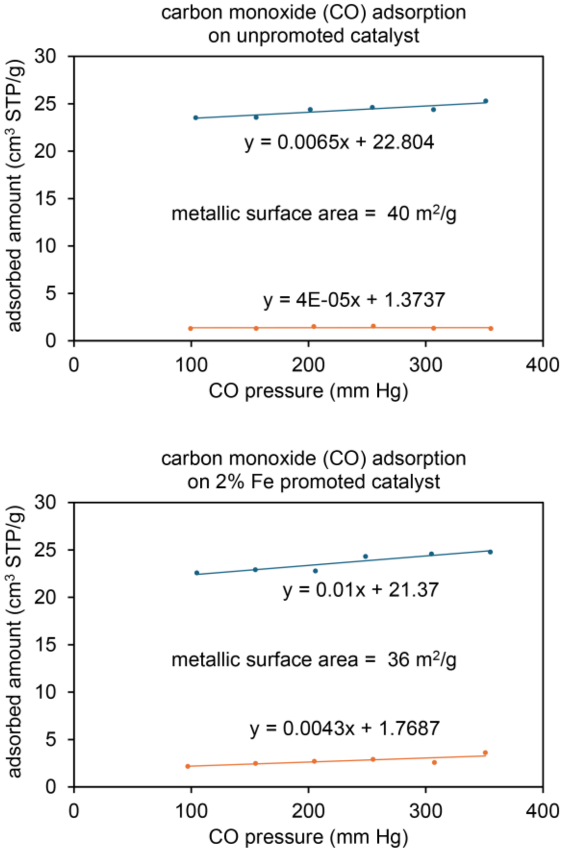

Figure S1: Adsorption of carbon monoxide (CO) to determine metal surface areas of nickel catalysts. The blue data is for the first adsorption, which includes both reversibly and irreversibly adsorbed CO. The orange data is for the second adsorption, which is the reversibly adsorbed CO. The difference between the blue trendline and the orange trendline quantifies the amount of irreversibly adsorbed CO, which was converted into a metallic surface area.

Figure S1 shows the results of this procedure for the unpromoted catalyst (at 0 °C) and for the Fe-promoted catalyst (at 33 °C). A 10-second equilibration interval was used for each adsorption point measurement. Using a vacuum pump, the sample was pumped down for one hour between the first and second isotherms. As shown in Figure S1, the metallic surface areas measured using this method were ~40 m^2/g for both the unpromoted and Fe-promoted catalysts. We previously reported a CO-measured metallic surface area of 50 m^2/g for the Fe-Cr doubly-promoted catalyst.[S16] Comparing these results to the total surface areas listed in the main text of

this article, approximately 40–50% of the surface area for each of these catalysts was in reduced metallic form.

Figure S2 shows adsorption isotherms of butylamine onto the unpromoted and Fe-Cr doubly-promoted Raney nickel catalysts at room temperature in methanol-water solvent under flowing hydrogen. These isotherms were satisfactorily described by the Langmuir model, which is a special case of the MD isotherm. As described in prior literature, addition of NaOH to the reaction mixture affects the activity and selectivity of reactions over sponge nickel catalysts.[S16-18] Various bases, salts, and acids have been tested as additives to sponge nickel catalysts during hydrogenation reactions.[S18, 19] For hydrogenation of nitriles to amines, the addition of NaOH increases the selectivity of the reaction towards primary amine.[S16, 17, 22, 23] As shown in Figure S2 and ref [S16], the addition of base decreases butylamine adsorption. Figure S2 and ref [S16] also show that the Fe-Cr doubly-promoted catalyst had lower butylamine adsorption compared to the unpromoted catalyst. These results explain why condensation reactions involving adsorbed butylamine as a one of the reactants are suppressed by base addition and by the Fe-Cr promoters.[S16]

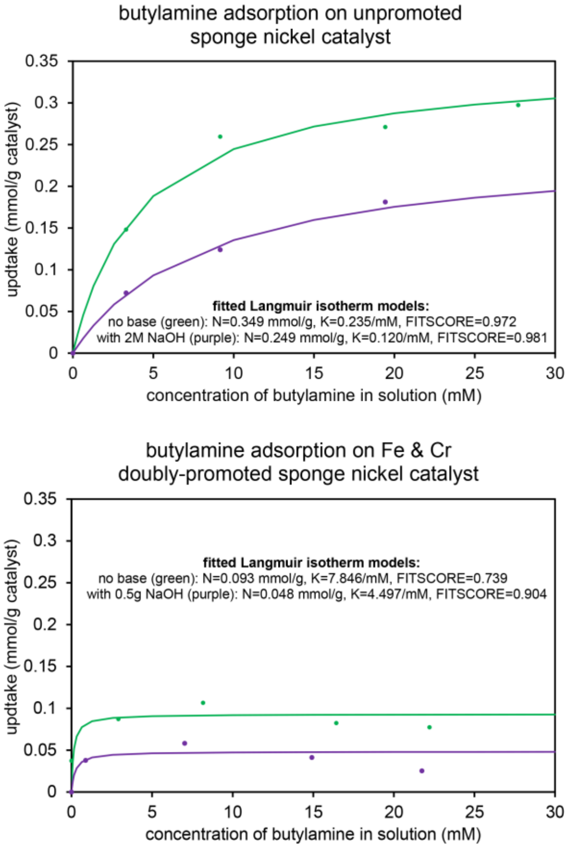

Figure S2: Adsorption of butylamine onto sponge nickel catalysts at room temperature in 50 mL methanol plus 10 mL water solvent under flowing hydrogen (1 atm). Results in the top panel are for the unpromoted sponge nickel catalyst. Results in the bottom panel are for the Fe and Cr doubly-promoted sponge nickel catalyst. Data collected in the absence of NaOH base addition is colored green. The addition of NaOH base suppressed butylamine adsorption, as shown by the data colored purple. The experimental data was fit to Langmuir adsorption isotherm models (solid curves). The Langmuir model isotherm is a special case of the MD model isotherm, corresponding to $\alpha = 1$, $m = 0$, $K_2 = 0$.

S3. Brunauer-Emmett-Teller Surface Identification (BETSI) plots

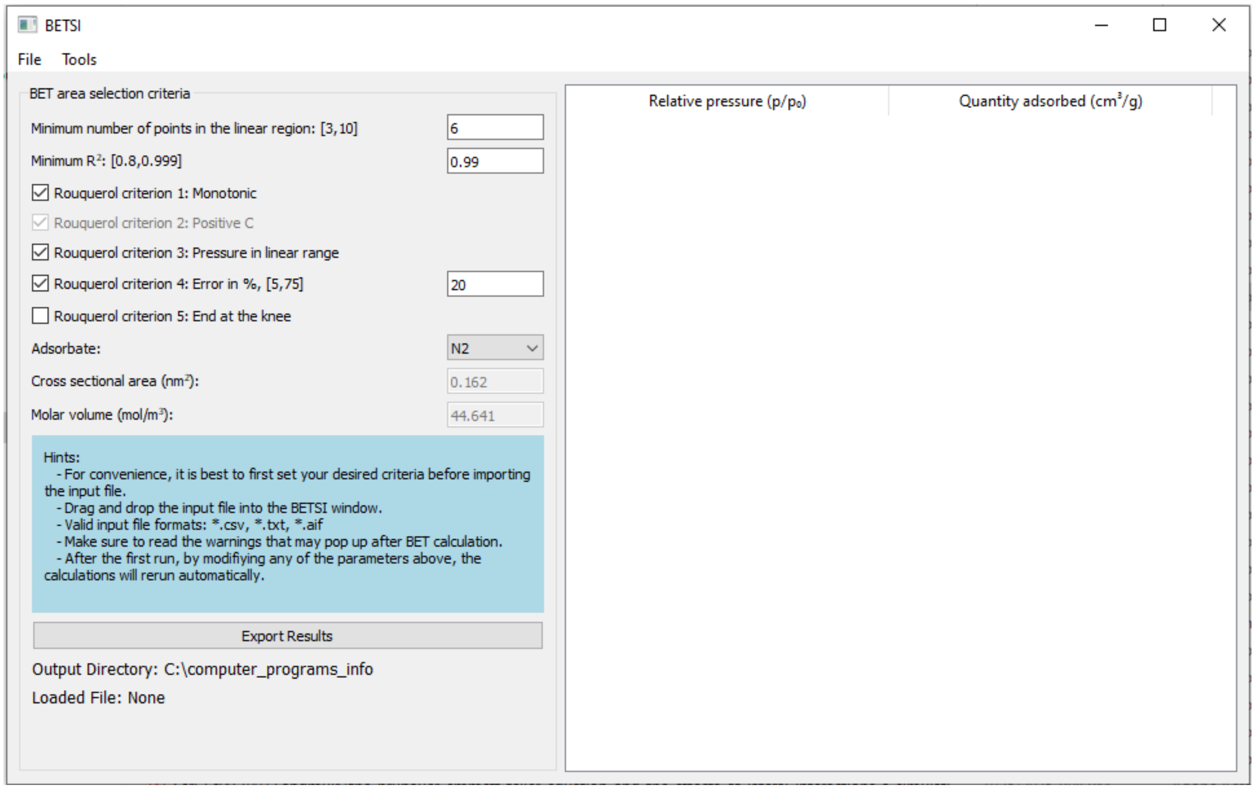

Figure S3: BETSI settings for liquid N_2 adsorption.

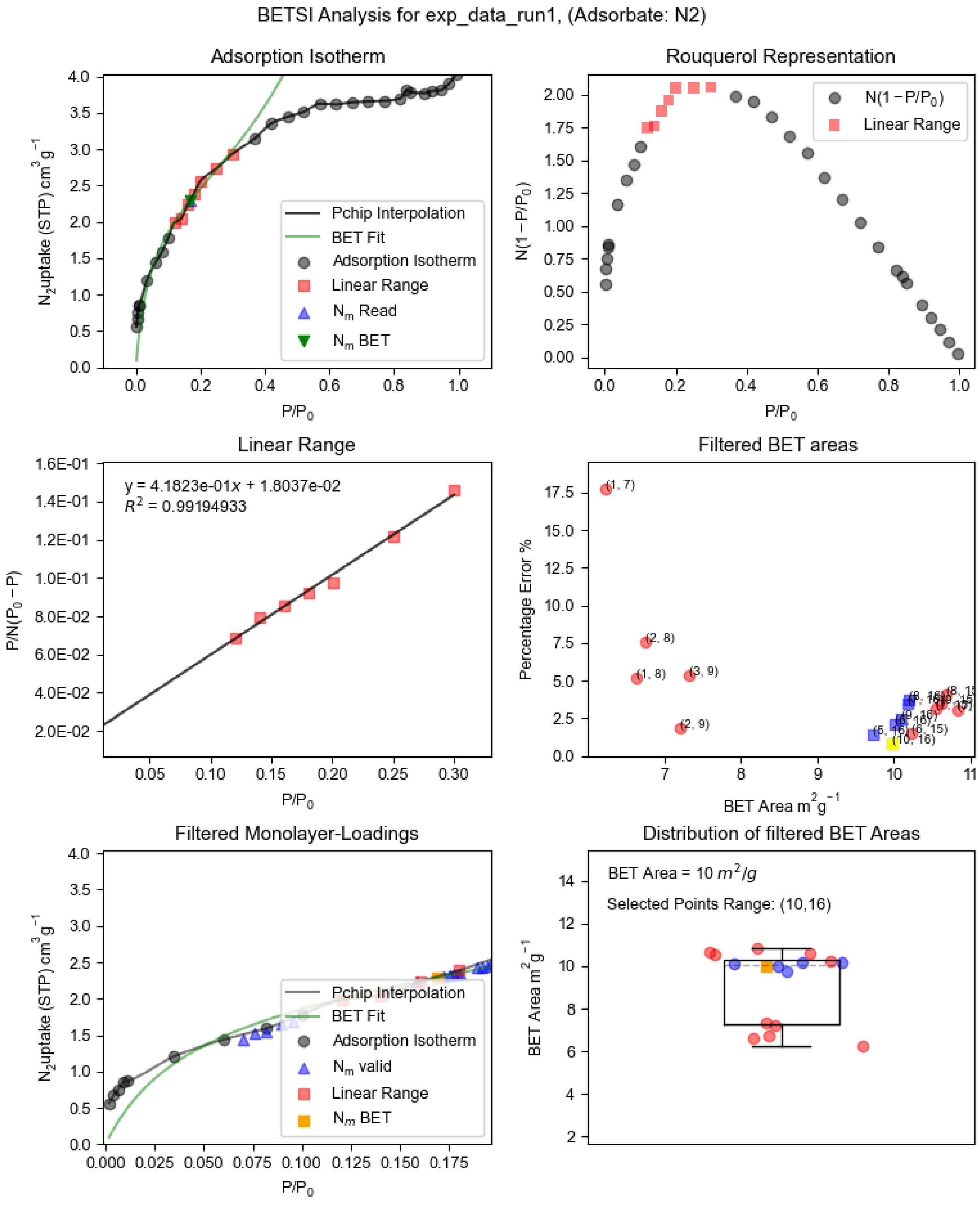

Figure S4: BETSI plots for liquid N_2 adsorption on porous Mn-Ni after 7 days leaching in concentrated aqueous acetic acid. These plots are for experimental run 1.

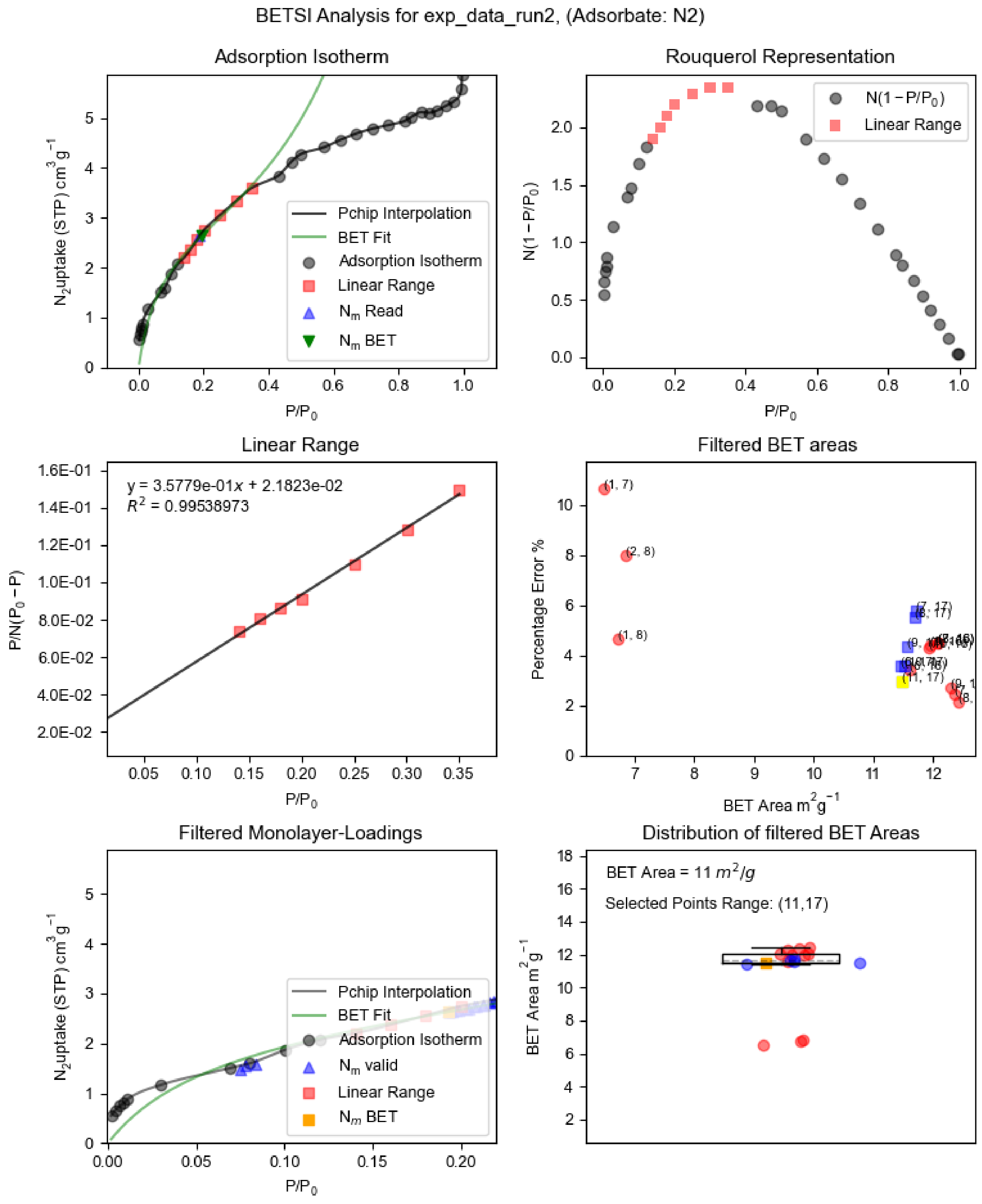

Figure S5: BETSI plots for liquid N_2 adsorption on porous Mn-Ni after 7 days leaching in concentrated aqueous acetic acid. These plots are for experimental run 2.

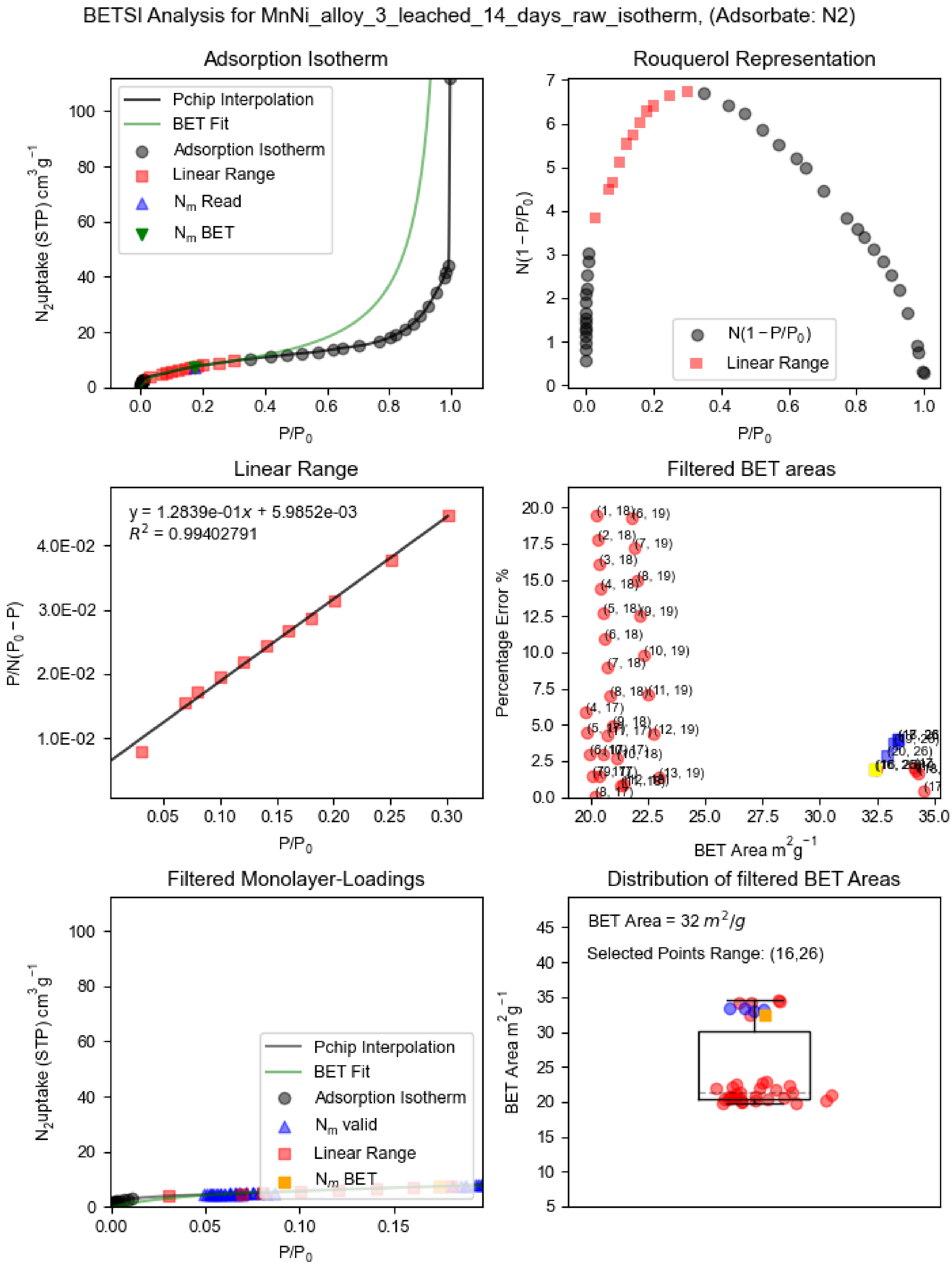

Figure S6: BETSI plots for liquid N_2 adsorption on porous Mn-Ni after 14 days leaching in concentrated aqueous acetic acid.

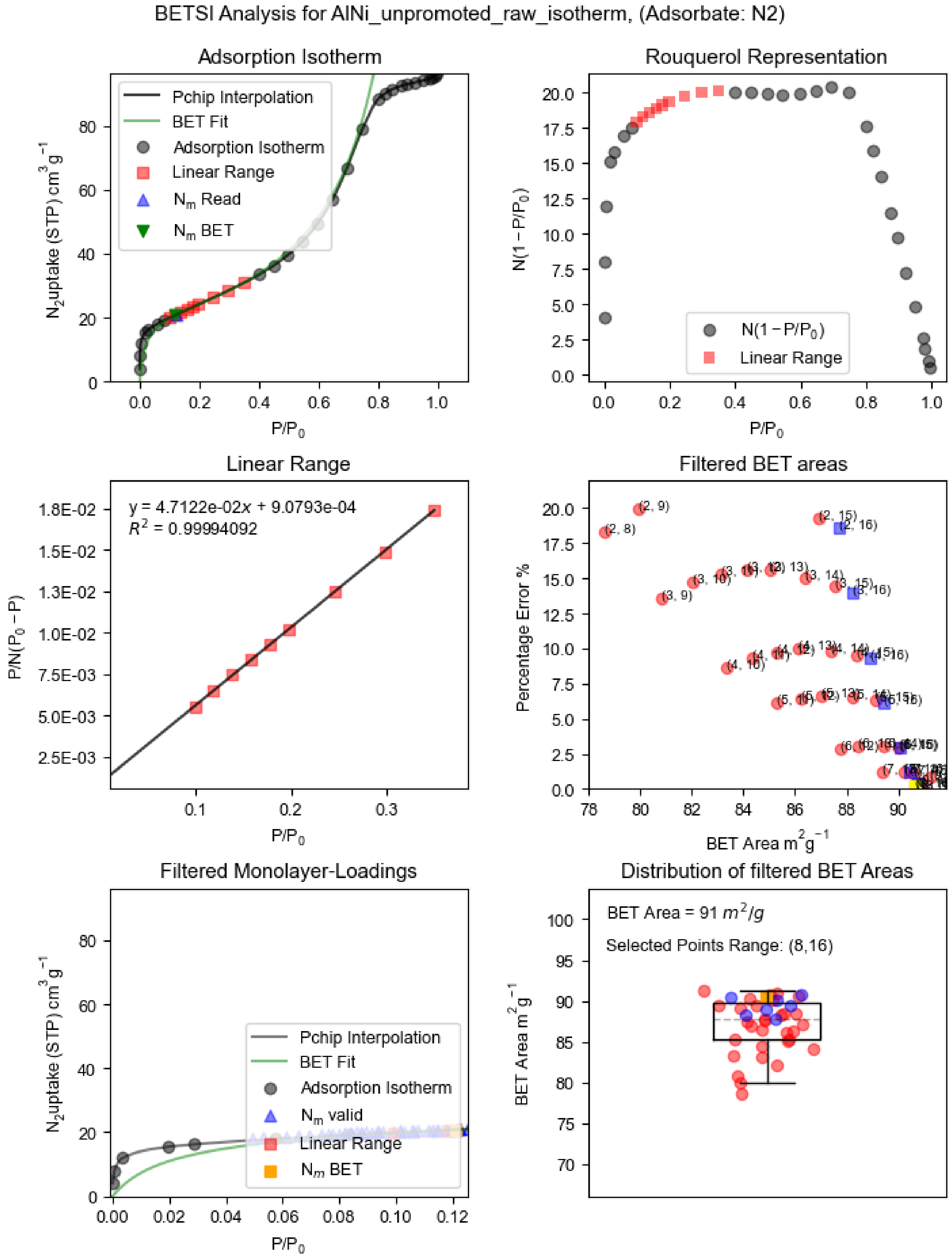

Figure S7: BETSI plots for liquid N_2 adsorption on porous Al-Ni material after leaching in concentrated aqueous NaOH.

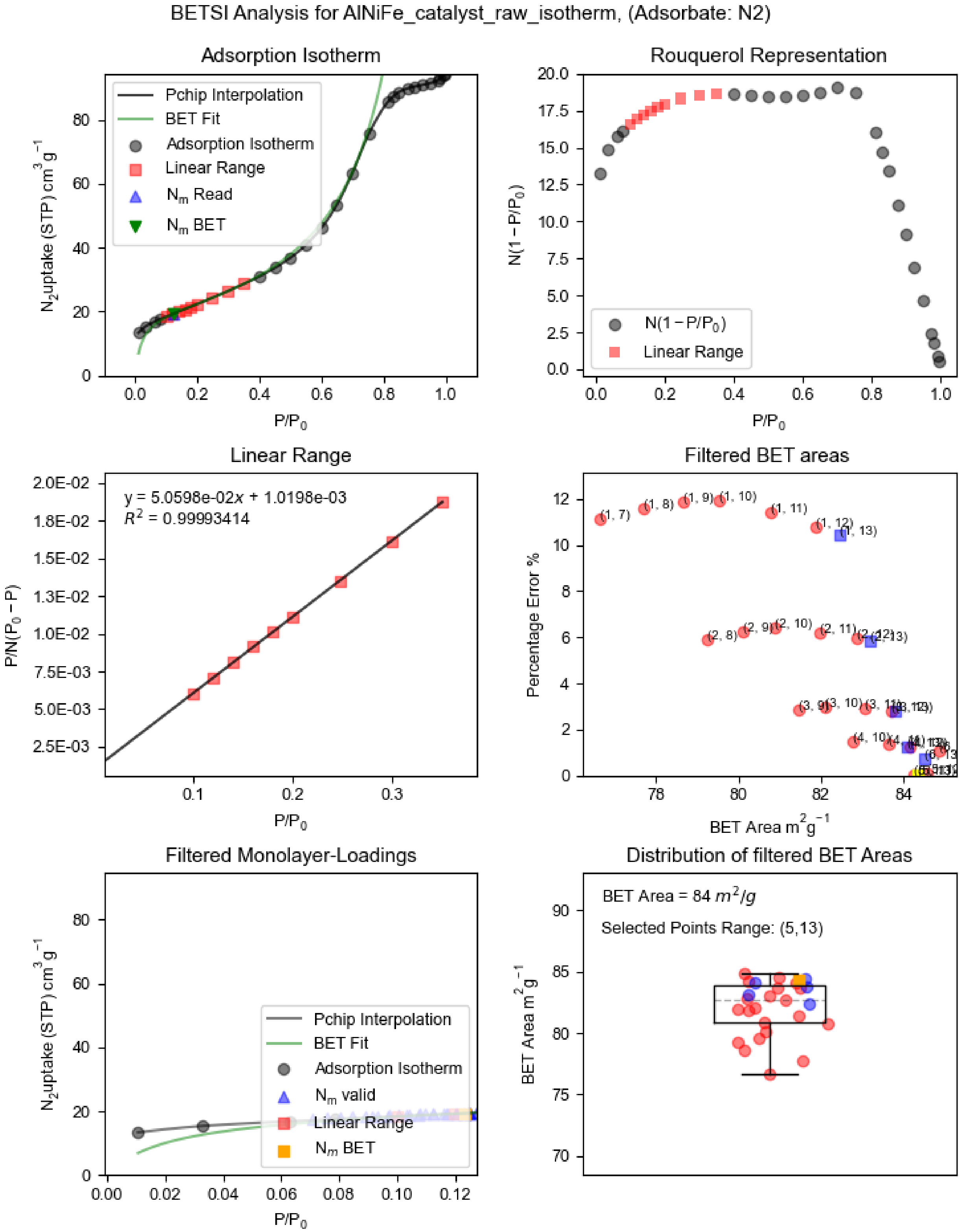

Figure S8: BETSI plots for liquid N_2 adsorption on porous Al-Ni-Fe material after leaching in concentrated aqueous NaOH.

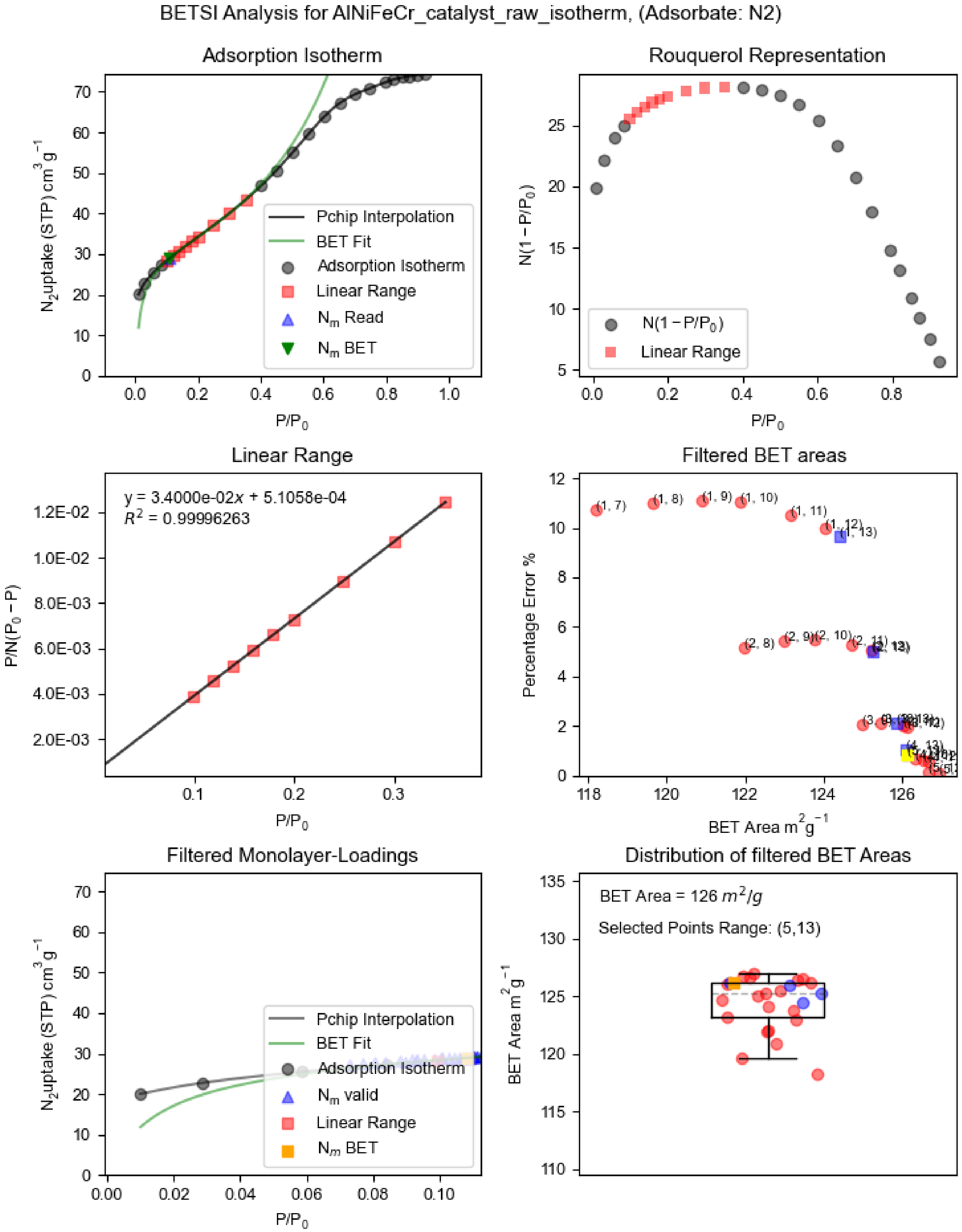

Figure S9: BETSI plots for liquid N_2 adsorption on porous Al-Ni-Fe-Cr material after leaching in concentrated aqueous NaOH.

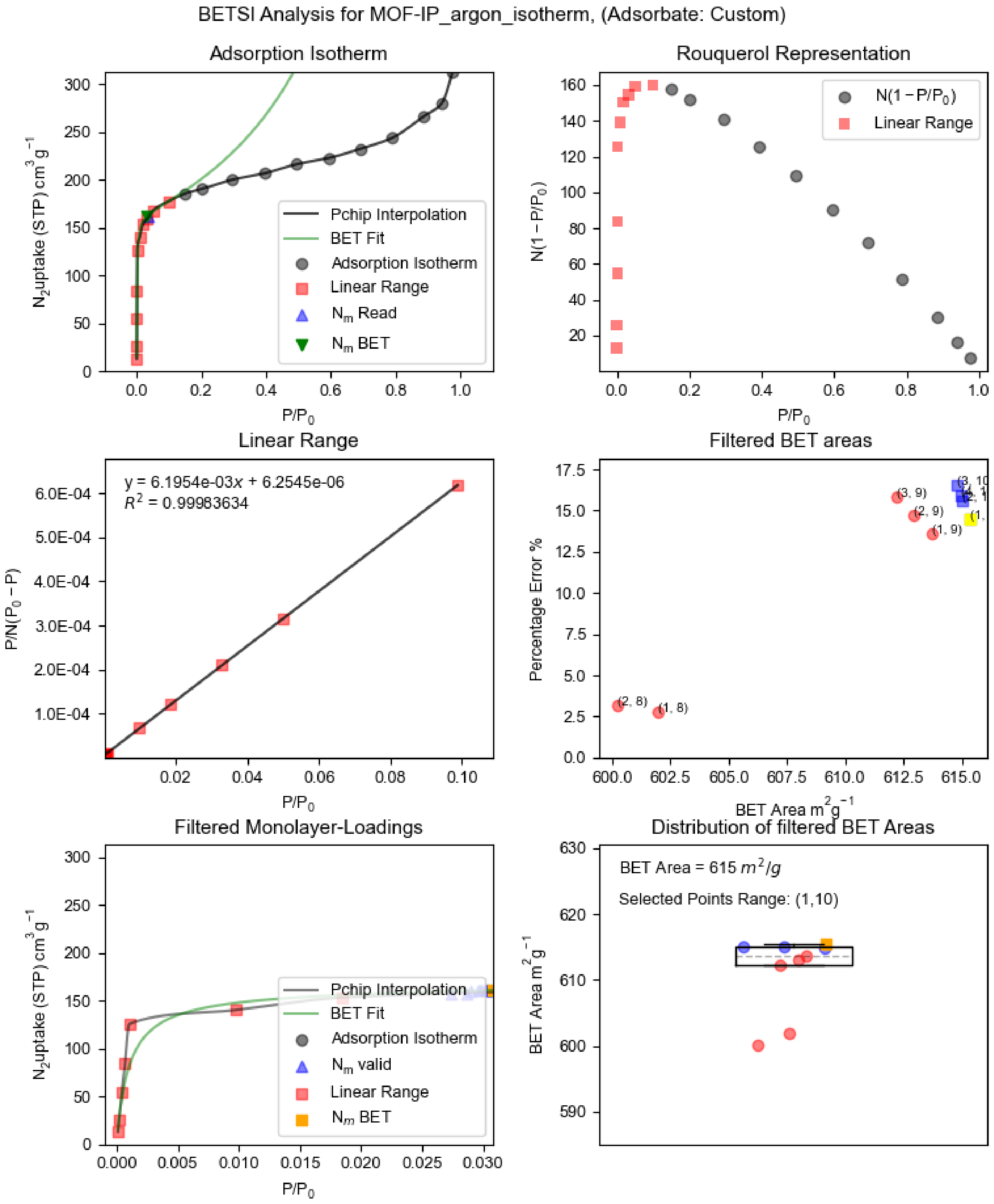

Figure S10: BETSI plots for Ar adsorption at 87 K on MOF-IP.

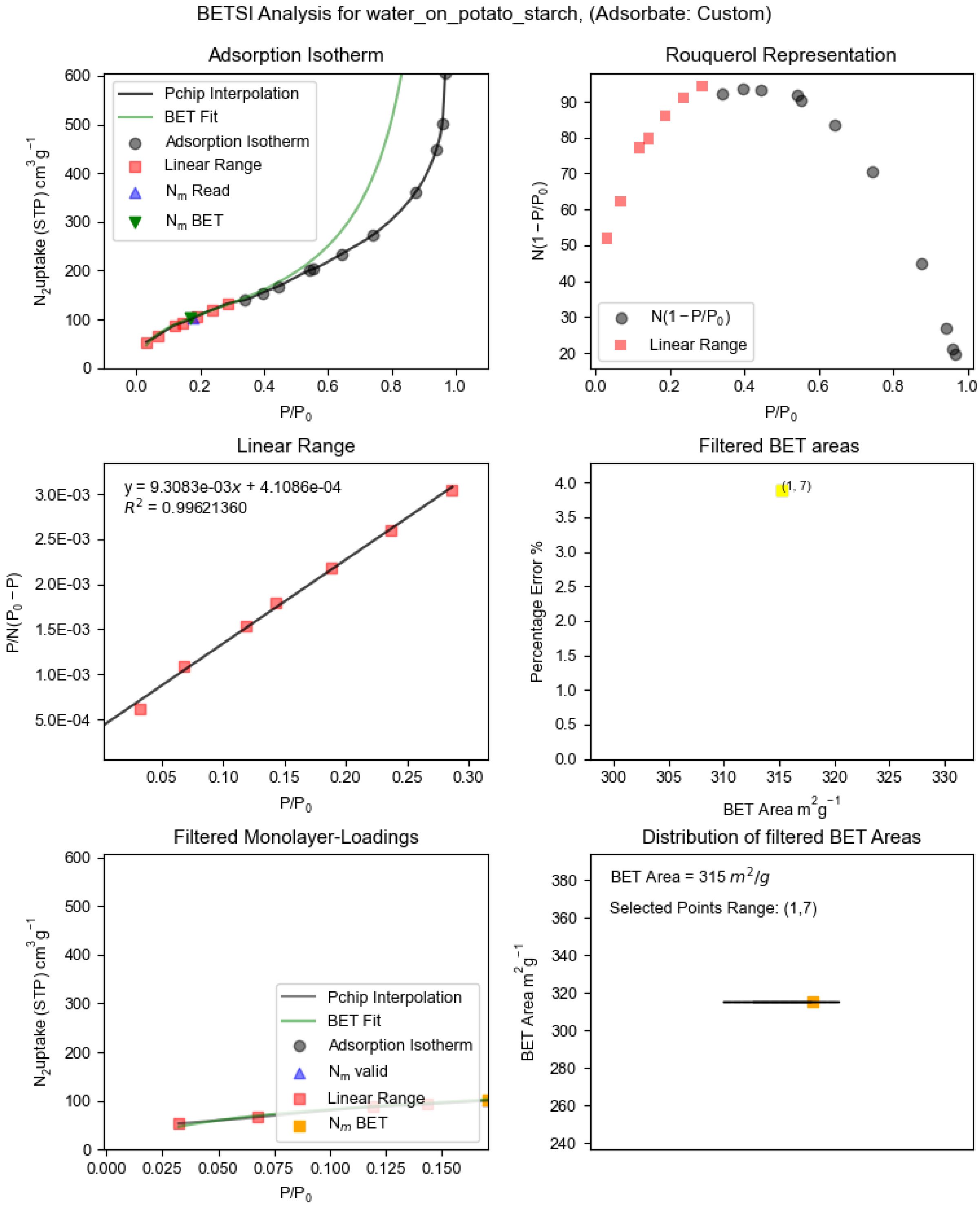

Figure S11: BETSI plots for water adsorption at room temperature on potato starch.

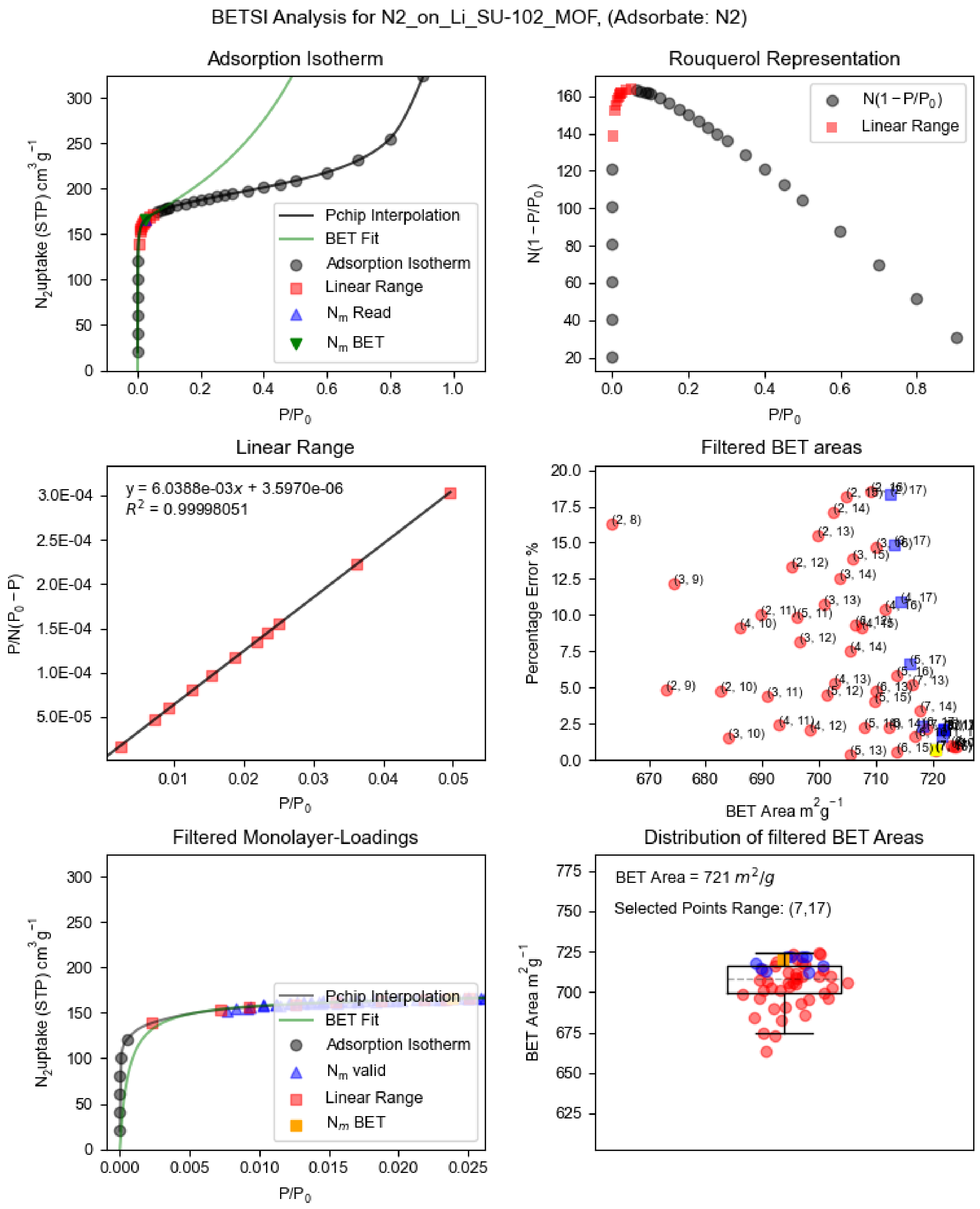

Figure S12: BETSI plots for liquid N_2 adsorption on Li-SU-102 MOF.

S4. Derivation of the form of condensation term

When m > 0 (i.e., multilayer adsorption), the condensation term makes the effective equilibrium constant for the last adsorption step (i.e., step number (1+m)) greater than K_2. The underlying physical reason for this is that in a multilayer adsorption layer (1+m) is surrounded on both sides by adsorbed adsorbate molecules when it is inserted. Since the adsorbate-adsorbate interaction is energetically favorable, the final layer (i.e., layer (1 + m)) has a stronger adsorption energy and hence larger effective adsorption equilibrium constant than K_2 for the previous multilayers.

For example, suppose that m = 10, so that there is a large number of adsorption layers. The first adsorption layer adjacent to each wall of the pore is governed by the equilibrium constant K_1. The subsequent multilayers (excluding the first and last layers) are governed by the equilibrium constant K_2. These layers are stacked up like building blocks, where each layer adsorbs on top of the underlying layer. A layer does not have to be completely full before the next layer is started. For example, part of the fourth layer can start stacking on filled portions of the third layer even if some other parts of the third layer have not yet been filled up. For these layers, a block (i.e., molecule) is placed on top of the underlying blocks (i.e., molecules). However, for the last adsorption layer (i.e., layer (1 + m)), the block is inserted as the last missing puzzle piece and is adjacent to blocks (i.e., adsorbed molecules) on both sides when it is inserted. This makes insertion of molecules into the final layer (i.e., layer (1 + m)) different from the previous multilayers. The goal of the condensation term is to capture the increased equilibrium constant for the last layer.

One option is to make the effective equilibrium for the last (aka 'nth') layer inversely proportional to $(1-x_A)$ as Pickett[S20] did: $[A_n S]=\left(x_A/(1-x_A)\right)[A_{(n-1)} S]$. This enforces complete condensation and complete pore filling as $x_A \to 1$. Pickett effectively assumed that $K_2 = 1$.

Two possible extensions to $K_2 \neq 1$ are possible. The first possible extension keeps $(1-x_A)$ in the denominator and sets $[A_{(1+m)} S]=\left(K_2 x_A/(1-x_A)\right)[A_m S]$, where we have now used (1+m) as the index for the last layer. This extension cannot be applied to adsorption at supercritical temperatures, because x_A cannot be defined as P/P_{sat} since P_{sat} does not exist at supercritical temperatures. The second possible extension replaces $(1-x_A)$ with $(1-K_2 x_A)$ in the denominator to give

$$[A_{(1+m)} S]=\left(K_2 x_A/(1-K_2 x_A)\right)[A_m S] \qquad \text{(S5)}$$

This second extension can even be applied to adsorption at supercritical temperatures up to the maximum pressure value $0 \le x_A = P_A \le P_{max} = (1/K_2)$. For this second extension, when $K_2 x_A = 1$ (which corresponds to $x_A = P_A = P_{max}$), the adsorption isotherm saturates to give $Q_A = (1+m)N$.

If m = 0, then adsorption is restricted to a single layer, so that Q_A should reduce to the Sips (for any α > 0 value) or Langmuir (for α = 1) isotherms. This suggests the condensation enhancement should be eliminated when m = 0. Accordingly, we can revise the above proposal to

$$[A_{(1+m)} S]=\left(1+\left(\frac{m}{m+1}\right)(C.T.-1)\right)(K_1 x_A)^{\alpha}(K_2 x_A)^{m}[S] \qquad \text{(S6)}$$

where C.T. is the 'condensation term'. If we tentatively postulate that $C.T. \approx (1-K_2 x_A)^{-1}$, then this would yield:

$$[A_{(1+m)}S] \approx \left(1+\frac{m}{(1+m)}\left(\left(\frac{1}{1-K_2x_A}\right)-1\right)\right)(K_1x_A)^{\alpha}(K_2x_A)^m[S] \quad \text{(tentative, not final form)} \tag{S7}$$

When $m \gg 1$, this becomes something close to eqn (S5) above. When m is close to zero, then the condensation enhancement is eliminated and eqn (S6) reduces to $[A_1S]=(K_1x_A)^{\alpha}[S]$. By using eqn (S6) instead of eqn (S5), the Sips and Langmuir isotherms are easily recovered in the $m \to 0$ limit.

The next observation concerns the value of the slope when $K_2x_A \to 1$. When using eqn (S7) to define adsorption for the last step, the total adsorbed amount can be arranged into the following form:

$$Q_A^{\text{tentative}} = N\frac{(1-K_2x_A)(\text{stuff\_1})+m(K_1x_A)^{\alpha}(K_2x_A)^m}{(1-K_2x_A)(\text{stuff\_2})+\left(\frac{m}{1+m}\right)(K_1x_A)^{\alpha}(K_2x_A)^m} \tag{S8}$$

where stuff_1 and stuff_2 remain finite in the $K_2x_A \to 1$ limit. Eqn (S8) yields the limit $Q_A^{\text{tentative}}=(1+m)N$ as $K_2x_A \to 1$. To analyze the slope in such a limit, consider $K_2x_A = 1-\varepsilon$, where ε is a positive infinitesimal, so that

$$\begin{aligned}\lim_{K_2x_A\to 1}\frac{dQ_A^{\text{tentative}}}{d(K_2x_A)} &= \frac{N(1+m)}{\varepsilon}\left(1-\frac{\varepsilon(\text{stuff\_1})+m(K_1x_A)^{\alpha}}{\varepsilon(1+m)(\text{stuff\_2})+m(K_1x_A)^{\alpha}}\right)\\ &= N(1+m)\left(\frac{(1+m)(\text{stuff\_2})-(\text{stuff\_1})}{m(K_1x_A)^{\alpha}}\right)\end{aligned} \tag{S9}$$

Examining eqn (S9), the slope $dQ_A^{\text{tentative}}/d(K_2x_A)$ is not necessarily zero in the limit $K_2x_A \to 1$.

However, once the pore has filled up due to capillary condensation, no more adsorption in the pore should be feasible as x_A (i.e., the gas pressure) increases, because the pore is already full. This implies the slope $dQ_A/d(K_2x_A)$ should approach zero after capillary condensation has occurred, and thus the above capillary condensation formula are incorrect. To make the slope approach zero as the pore fills due to capillary condensation, we can simply raise the $(1-K_2x_A)$ term to a power slightly greater than one. Specifically, modify eqn (S7) to give

$$[A_{(1+m)}S] \approx \left(1+\frac{m}{(1+m)}\left(\left(\frac{1}{1-K_2x_A}\right)^{3/2}-1\right)\right)(K_1x_A)^{\alpha}(K_2x_A)^m[S] \tag{S10}$$

so the modifications of eqn (S8) and (S9) become

$$Q_A^{\text{tentative}} = N\frac{(1-K_2x_A)^{3/2}(\text{stuff\_1})+m(K_1x_A)^{\alpha}(K_2x_A)^m}{(1-K_2x_A)^{3/2}(\text{stuff\_2})+\left(\frac{m}{1+m}\right)(K_1x_A)^{\alpha}(K_2x_A)^m} \tag{S11}$$

$$\begin{aligned}\lim_{K_2x_A\to 1}\frac{dQ_A^{\text{tentative}}}{d(K_2x_A)} &= \frac{N(1+m)}{\varepsilon}\left(1-\frac{\varepsilon^{3/2}(\text{stuff\_1})+m(K_1x_A)^{\alpha}}{\varepsilon^{3/2}(1+m)(\text{stuff\_2})+m(K_1x_A)^{\alpha}}\right)\\ &= N(1+m)\left(\frac{(1+m)(\text{stuff\_2})-(\text{stuff\_1})}{m(K_1x_A)^{\alpha}}\right)\varepsilon^{1/2}=0\end{aligned} \tag{S12}$$

Examining eqn (S12), the slope $dQ_A^{\text{tentative}}/d(K_2x_A)$ is zero in the limit $K_2x_A \to 1$, because this limit corresponds to $\varepsilon \to 0$ and the slope is proportional to $\varepsilon^{1/2}$.

In microporous and mesoporous materials, the saturation vapor pressure within a capillary is reduced relative to the saturation pressure of the pure fluid.[S21] Consequently, small narrow pores undergo capillary condensation when $x_A = x_A^{cc} < 1$, and this in turn implies that $K_2 > 1$ for such small pores. Examining eqn (S10), the quantity $[A_{(1+m)} S]$ becomes ill-behaved for $K_2 x_A > 1$. However, physically, it is perfectly reasonable for the applied pressure to exceed the capillary condensation pressure. For example, suppose that in a N_2 adsorption experiment at 77 K, some of the small diameter pores undergo capillary condensation when $x_A = x_A^{cc} = 0.75$ which corresponds to a capillary condensation pressure of 0.75 atmospheres. For these pores, $K_2 = 4/3$. Now, it is perfectly reasonable to experimentally measure the adsorbed amounts for gas pressures above the capillary condensation pressure, and this corresponds to measurements within the range $x_A^{cc} < x_A < 1$. Hence, we must remove the singularity in the capillary condensation formula that occurs when $K_2 x_A = 1$.

The easiest and most straightforward way to do this is to replace $1/(1 - K_2 x_A)$ with the first several terms of its series expansion:

$$\left(\frac{1}{1-K_2 x_A}\right)^{3/2} \approx \left(1+(K_2 x_A)+(K_2 x_A)^2+(K_2 x_A)^3+(K_2 x_A)^4\right)^{3/2} = \text{C.T.} \quad \text{(S13)}$$

When $0 \le (K_2 x_A) \ll 1$, the left and right sides of eqn (S13) are approximately equal to each other. When $K_2 x_A = 1$, then the condensation term (C.T.) becomes equal to $5^{(3/2)}$ = 11.18 ... which corresponds to approximately an order of magnitude enhancement relative to 1.0. (An order of magnitude enhancement is the 'appropriate' amount.) By using eqn (S13), the C.T. is monotonically increasing and does not exhibit any singularities. Revisiting the above example, for the pores undergoing capillary condensation near $x_A = x_A^{cc} = 0.75$ (with $K_2 = 4/3$), the C.T. will become much larger than one as the gas pressure approaches and exceeds the capillary condensation pressure. Eqn (S13) has the effect of 'softening' the capillary condensation, so that the singularity is removed. This also has the distinct advantage of providing smoother behavior for fluids at supercritical temperatures where P_{sat} does not exist. This extends the feasible range of allowed pressures and x_A values to

$$0 \le x_A, P_A \le \infty \quad \text{(S14)}$$

In summary, the specific form of condensation term used in this work is physically motivated.

S5. Proof that Q_A is non-negative and monotonically increasing

S5.1 Proof that the value of $Q_{A,g}^{MD}$ is always non-negative.

$Q_{A,g}^{MD}$ can be rewritten in the form

$$Q_{A,g}^{MD} = N_g \frac{(K1_g x_A)^{\alpha_g}(\text{term\_1}+\text{term\_2})}{1+(K1_g x_A)^{\alpha_g}(\text{term\_3}+\text{term\_4})} \quad \text{(S15)}$$

Since $N_g \ge 0$, $K1_g \ge 0$, $x_A \ge 0$, and $\alpha_g > 0$, it follows that $Q_{A,g}^{MD}$ is necessarily always non-negative if it can be proved that term_1, term_2, term_3, and term_4 are all non-negative.

Using the substitution of variables,

$$y = K2_g x_A \quad \text{(S16)}$$

each of these four terms expands as

$$\text{term\_1} = \frac{1-(m_g+2)y^{m_g+1}+(m_g+1)y^{m_g+2}}{(1-y)^2} \tag{S17}$$

$$\text{term\_2} = m_g y^{m_g}(\text{C.T.}-1) \tag{S18}$$

$$\text{term\_3} = \frac{1-y^{m_g+1}}{1-y} \tag{S19}$$

$$\text{term\_4} = \left(\frac{m_g}{m_g+1}\right) y^{m_g}(\text{C.T.}-1) \tag{S20}$$

Since $K2_g \geq 0$ and $x_A \geq 0$, it directly follows that

$$(\text{C.T.}-1) = \left((1+y+y^2+y^3+y^4)^{3/2}-1\right) \geq 0 \tag{S21}$$

Since $m_g \geq 0$, it follows from eqn (S18), (S20), and (S21) that

$$\text{term\_2} \geq 0 \tag{S22}$$
$$\text{term\_4} \geq 0$$

For term_3, when $y < 1$ then both $(1-y)$ and $(1-y^{m_g+1})$ are positive, leading to $\text{term\_3} > 0$. When $y > 1$, then both $(1-y)$ and $(1-y^{m_g+1})$ are negative, leading to $\text{term\_3} > 0$. In the limit $y \to 1$, then

$$\lim_{y\to 1}\left(\frac{1-y^{m+1}}{1-y}\right) = (m+1) > 0 \tag{S23}$$

Therefore, in all cases,

$$\text{term\_3} \geq 0 \tag{S24}$$

A graphical approach will be used to show that

$$\text{term\_1} \geq 0 \tag{S25}$$

Term_1 can be visualized graphically as a family of curves in which m is treated as a curve parameter and y is treated as the independent variable. Since $y = K2_g x_A$ (eqn (S16)), if every such curve is non-negative and increases monotonically with increasing y ≥ 0, then it is proved that term_1 is non-negative and always increases monotonically with increasing x_A. The plotted curves shown in Figure S13 do indeed show that each constant m curve is non-negative and increases monotonically with increasing y ≥ 0.

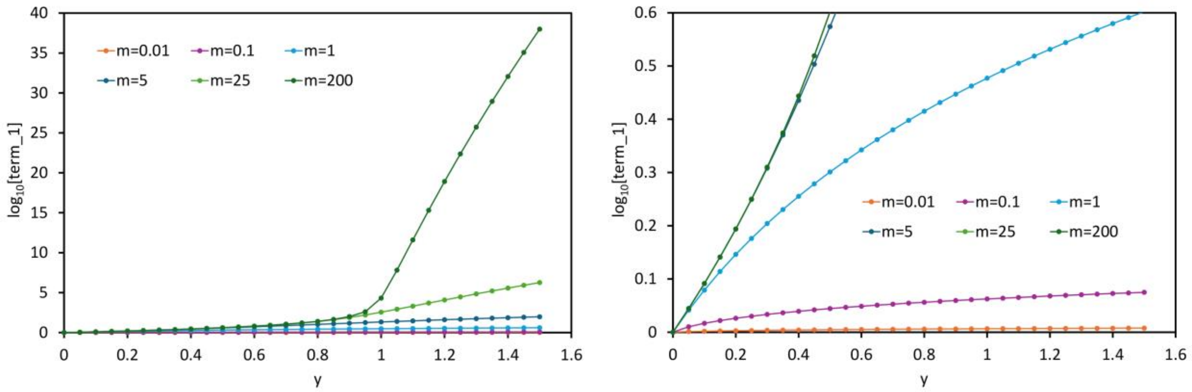

Figure S13: Plots demonstrating that term_1 is non-negative and increases monotonically with increasing y value for each fixed m value. The underlying numeric values (see spreadsheet in the enclosed zip archive) were carefully examined to confirm this. The right-hand panel replots the same data as the left-hand panel, except it uses a different y-axis range to better show the m = 0.01 and m =0.1 curves.

Since term_1, term_2, term_3, and term_4 are all non-negative, it is proved that in all cases

$$Q_{A,g}^{MD} \geq 0 \tag{S26}$$

S5.2 Proof that $Q_{A,g}^{MD}$ increases monotonically with increasing x_A.

We begin by rewriting $Q_{A,g}^{MD}$ as the product of two non-negative factors times N_g:

$$Q_{A,g}^{MD} = N_g\left(\text{factor\_1}\right)\left(\text{factor\_2}\right) \tag{S27}$$

where the first factor is

$$0 \leq \text{factor\_1} = \frac{\left(K1_g x_A\right)^{\alpha_g}\left(\text{term\_3} + \text{term\_4}\right)}{1 + \left(K1_g x_A\right)^{\alpha_g}\left(\text{term\_3} + \text{term\_4}\right)} < 1 \tag{S28}$$

and the second factor is

$$\text{factor\_2} = \frac{\left(\text{term\_1} + \text{term\_2}\right)}{\left(\text{term\_3} + \text{term\_4}\right)} \geq 0 \tag{S29}$$

Note that if some quantity q ≥ 0 is monotonically increasing then, q/(1 + q) is also monotonically increasing. Examining eqn (S28), this means that factor_1 will be monotonically increasing if

$$q = \left(K1_g x_A\right)^{\alpha_g}\left(\text{term\_3} + \text{term\_4}\right) \tag{S30}$$

is monotonically increasing with increasing x_A. Since $\alpha_g > 0$, when $K1_g > 0$ it follows that $\left(K1_g x_A\right)^{\alpha_g}$ is monotonically increasing with increasing x_A. Accordingly, q will be monotonically increasing if $\left(\text{term\_3} + \text{term\_4}\right)$ is monotonically increasing. Let's examine term_3 and term_4 separately.

Term_3 can be visualized graphically as a family of curves in which m is treated as a curve parameter and y is treated as the independent variable. Since $y = K2_g x_A$ (eqn (S16)), if every such curve is non-negative and increases monotonically with increasing y ≥ 0, then it is proved that

term_3 is non-negative and always increases monotonically with increasing x_A. The plotted curves shown in Figure S14 do indeed show that each constant m curve is non-negative and increases monotonically with increasing $y \geq 0$.

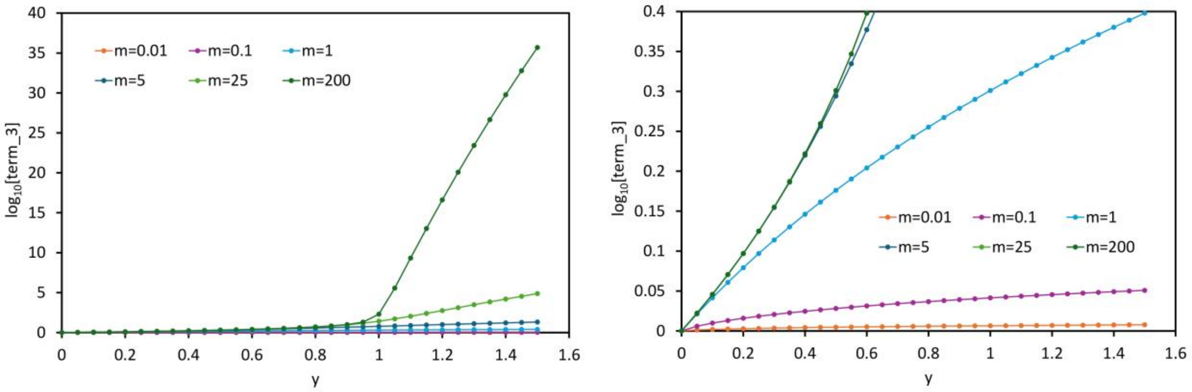

Figure S14: Plots demonstrating that term_3 is non-negative and increases monotonically with increasing y value for each fixed m value. The underlying numeric values (see spreadsheet in the enclosed zip archive) were carefully examined to confirm this. The right-hand panel replots the same data as the left-hand panel, except it uses a different y-axis range to better show the m = 0.01 and m =0.1 curves.

Now, let's examine the derivative of term_4:

$$\begin{aligned}\frac{d(\text{term\_4})}{dy} &= \left(\frac{m_g}{m_g+1}\right)\frac{d}{dy}\left(y^{m_g}\left(\left(1+y+y^2+y^3+y^4\right)^{3/2}-1\right)\right) \\ &= \left(\frac{m_g}{m_g+1}\right)\left(m_g y^{m_g-1}\left(\left(1+y+y^2+y^3+y^4\right)^{3/2}-1\right)+\frac{3}{2}y^{m_g}\sqrt{\left(1+y+y^2+y^3+y^4\right)}\left(1+2y+3y^2+4y^3\right)\right)\end{aligned} \quad (S31)$$

$$\frac{d(\text{term\_4})}{dy} \geq 0 \quad (S32)$$

This proves term_4 is monotonically increasing.

Since both term_3 and term_4 are monotonically increasing, this concludes the proof that factor_1 always increases monotonically with increasing x_A.

A graphical approach will be used to show that factor_2 is always increases monotonically with increasing x_A. This factor can be written as a function of the two variables y and m:

$$\text{factor\_2}[y,m] = \frac{\dfrac{1-(m+2)y^{m+1}+(m+1)y^{m+2}}{(1-y)^2}+my^m\left(\left(1+y+y^2+y^3+y^4\right)^{3/2}-1\right)}{\dfrac{1-y^{m+1}}{1-y}+\left(\dfrac{m}{m+1}\right)y^m\left(\left(1+y+y^2+y^3+y^4\right)^{3/2}-1\right)} \quad (S33)$$

This function can be visualized graphically as a family of curves in which m is treated as a curve parameter and y is treated as the independent variable. Since $y = K2_g x_A$ (eqn (S16)), if every such curve increases monotonically with increasing $y \geq 0$, then it is proved that factor_2 always increases monotonically with increasing x_A. The plotted curves shown in Figure S15 do indeed show that each constant m curve increases monotonically with increasing $y \geq 0$.

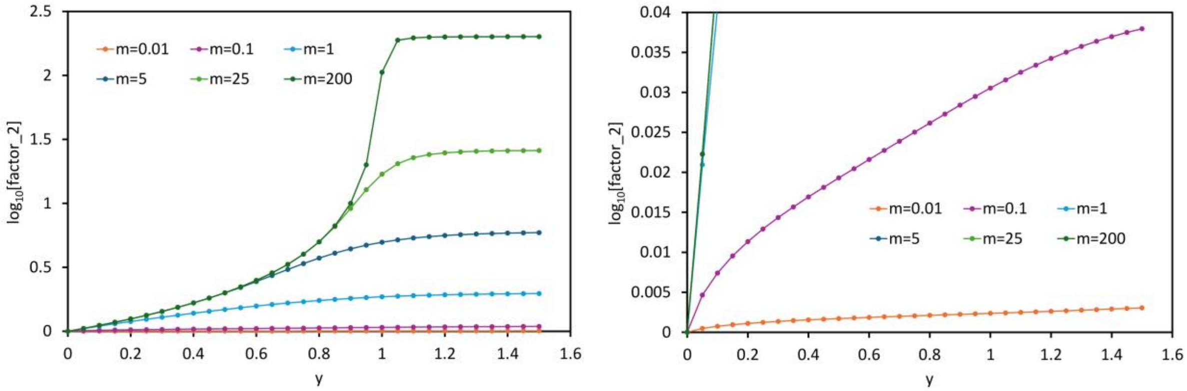

Figure S15: Plots demonstrating that factor_2 increases monotonically with increasing y value for each fixed m value. The underlying numeric values (see spreadsheet in the enclosed zip archive) were carefully examined to confirm this. The right-hand panel replots the same data as the left-hand panel, except it uses a different y-axis range to better show the m = 0.01 and m =0.1 curves.

Since factor_1 and factor_2 are always non-negative and increase monotonically with increasing x_A, it directly follows that the product of these two factors always increases monotonically with increasing x_A. This completes the proof that $Q_{A,g}^{MD}$ always increases monotonically with increasing x_A.

S6. How to use the enclosed programs to compute MD model optimized parameters, surface area, and confidence intervals

S6.1 Worked examples and instructions including whole isotherm surface area calculation

This section describes the program usage when computing MD model parameters and whole isotherm surface areas from N_2 adsorption (at 77 K), argon adsorption (at 87 K), or moisture adsorption (at room temperature).

Step #1: Make sure that you have the Optimization Toolbox installed in Matlab. (Note: This program uses the fmincon function that is part of Matlab's Optimization Toolbox, so you must have this toolbox installed to use it.)

Step #2: In the Supporting Information zip archive, navigate to the folder program_files/MD_program_with_surface_area. Unzip this folder. In Matlab, navigate to the MD_program_with_surface_area folder and open it as your current working directory.

Step #3: Open the experimental_adsorption_data.m file. Update the following items:

- In the array xA_data, put your experimental P/P_0 data here, where P_0 is the saturation pressure at the experiment's temperature. The first entry should be 0.0000 and correspond to zero pressure. The remaining entries should be monotonically increasing and ≤1.0000.
- In the array QA_exp, put your experimental absolute adsorption amounts (in mmol/g) here. The first entry should be 0.0000 and corresponds to no uptake at zero pressure. The remaining entries should be monotonically increasing. Note: The pair (xA_data(j),QA_exp(j)) represents the P/P_0 and Q_A value for the jth experimental datapoint. Always include (0.00,0.00) as your first datapoint.

- Set the value of the adsorbate_cross_section variable equal to the appropriate molecule cross-sectional area (in nm^2). The following values are suggested: 0.162 for N_2 at 77 K, 0.142 for Ar at 87 K, 0.114 for H_2O at 298 K.

Step #4: Run the experimental_adsorption_data.m file. You can do this by clicking on the green 'Run' triangle or by typing experimental_adsorption_data in Matlab's command window. The program may take up to 2 hours to run.

Step #5: When the program runs, it prints the output to a file called 'diary' in the current working directory. Rename this file to something more useful. (For example, water_adsorp_on_activated_carbon_AT400R1t75_Matlab_opt_with_confidence_intervals.txt) Open this file in your favorite text editor. Search for 'params' to get to the part where the optimized parameter values are printed. The optimized parameter values, fitscore, and surface area are printed as alpha = #, K1 = #, K2 = #, m = #, N = #, fitscore = #, surface_area = #, where the calculated value is printed in place of '#'. Next, scroll to the bottom of this file to read out the computed 95% confidence interval for each model parameter and the surface area. These are clearly labeled as 'The 95% confidence interval on m is ....', 'The 95% confidence interval on surface area (m^2/g) is ....', etc.

Worked examples: The following folders in the Supporting Information zip archive were run with the above procedure and provide worked examples: argon_adsorption_on_MOF_IP, Li_SU_102_MOF (with subfolders for N_2 and water adsorption), N2_adsorption_on_Al_fumarate, N2_adsorption_on_IRMOF_1, N2_adsorption_on_sponge_nickel_materials (with subfolders for five different materials), water_adsorption_on_activated_carbon_AT400R1t75, water_adsorption_on_Ni_DOBDC, water_adsorption_on_potato_starch, water_adsorption_on_UiO66.

S6.2 Worked examples and instructions without surface area calculation

This section describes the program usage when computing MD model parameters without surface area calculation. This is useful for adsorbates such as carbon dioxide, methane, alkanes, proteins, dyes, organic compounds, etc.

Step #1: Make sure that you have the Optimization Toolbox installed in Matlab. (Note: This program uses the fmincon function that is part of Matlab's Optimization Toolbox, so you must have this toolbox installed to use it.)

Step #2: In the Supporting Information zip archive, navigate to the folder program_files/MD_program_without_surface_area. Unzip this folder. In Matlab, navigate to the MD_program_without_surface_area folder and open it as your current working directory.

Step #3: Open the experimental_adsorption_data.m file. Update the following items:

- In the array xA_data, put your experimental data here for concentration or pressure. You can use any units for concentration, pressure, etc. The first entry should be 0.0000 and correspond to zero concentration or zero pressure. The remaining entries should be monotonically increasing.
- In the array QA_exp, put your experimental absolute adsorption amounts here. You can use any units. The first entry should be 0.0000 and corresponds to no uptake at zero concentration or zero pressure. The remaining entries should be monotonically increasing.

Note: The pair (xA_data(j),QA_exp(j)) represents the x_A and Q_A value for the jth experimental datapoint. Always include (0.00,0.00) as your first datapoint.

Step #4: Run the experimental_adsorption_data.m file. You can do this by clicking on the green 'Run' triangle or by typing experimental_adsorption_data in Matlab's command window. The program may take up to 2 hours to run.

Step #5: When the program runs, it prints the output to a file called 'diary' in the current working directory. Rename this file to something more useful. (For example, CO2_adsorbed_on_Zeolite_13X_Matlab_opt_with_confidence_interval.txt) Open this file in your favorite text editor. Search for 'params' to get to the part where the optimized parameter values are printed. The optimized parameter values and fitscore are printed as alpha = #, K1 = #, K2 = #, m = #, N = #, fitscore = #, where the calculated value is printed in place of '#'. Next, scroll to the bottom of this file to read out the computed 95% confidence interval for each model parameter. These are clearly labeled as 'The 95% confidence interval on m is ....', 'The 95% confidence interval on N is ....', etc.

Please note that α and m are dimensionless. The units for K1 and K2 are the inverse of units for x_A. For example, if x_A is in bar, then K1 and K2 are in bar^{-1}. If x_A is in mmol/L, then K1 and K2 are in L/mmol. The units for N are the same as the units for Q_A. For example, if Q_A is in mmol/g then N is also in mmol/g. If Q_A is in mg/g then N is also in mg/g.

Worked examples: The following folders in the Supporting Information zip archive were run with the above procedure and provide worked examples:
aqueous_red_dye_on_beechwood, benzene_adsorption_on_Zn_MOF,
CH4_on_MgO_type_6_isotherm, CO2_adsorption_on_Zeolite_13X,
H2_adsorption_on_IRMOF_1, O2_adsorption_on_Co_MOF,
protein_adsorption_on_hydrophobic_interaction_chromatography_resins (with subfolders for five different materials).

S6.3 Program procedure and solver settings

Figure S16 illustrates the process used to optimize MD model parameters and compute 95% confidence intervals.

The program automatically generates several different initial guesses, which have different m values. The objective is to formulate several initial guesses that are very different from each other so the solver starts from different locations. This enhances the probability that one of the solver algorithms will find the global minimum starting from at least one of the several different initial guesses.

In the current code version, the initial guess generation is summarized as follows. It seems reasonable to set $\alpha = 1.0$ as an initial guess. The value of m is set to various values (e.g., 0.5, 2, 4, or 6) in different initial guesses. If we assume the adsorption isotherm approaches saturation near the highest included experimental concentration or pressure, this means that N is approximately equal to QA_exp[Ndata]/(1 + m). So, this value is used as the initial guess for N. The initial guess for K1 equals the initial slope of the adsorption isotherm divided by N, as quantified by the first four datapoints: K1 = (QA_exp[4] - QA_exp[1])/(N*(xA_data[4] - xA_data[1])). If surface area calculation is included, then the initial guess for K2 is set to 1.0, since this represents the bulk

liquid interaction value. If surface area calculation is not included, then the initial guess for K2 is set to 1.0/xA_data[Ndata] which corresponds to K_2x_A approaching unity near the highest x_A value.

Ultimately, initial guesses cannot be 'correct' or 'incorrect' as they just serve as starting points for the solver. So far, with a couple dozen different adsorption isotherms analyzed to date, these four initial guesses were sufficient starting points to locate the global minimum solution in all cases. However, if through the process of time involving the analysis of more adsorption isotherms new situations are discovered in which these four starting points are not sufficient to locate the global minimum solution, then new initial guesses should be added to the list in the run_fmincon_optimization.m and run_fmincon_optimization_with_SurfaceArea.m files.

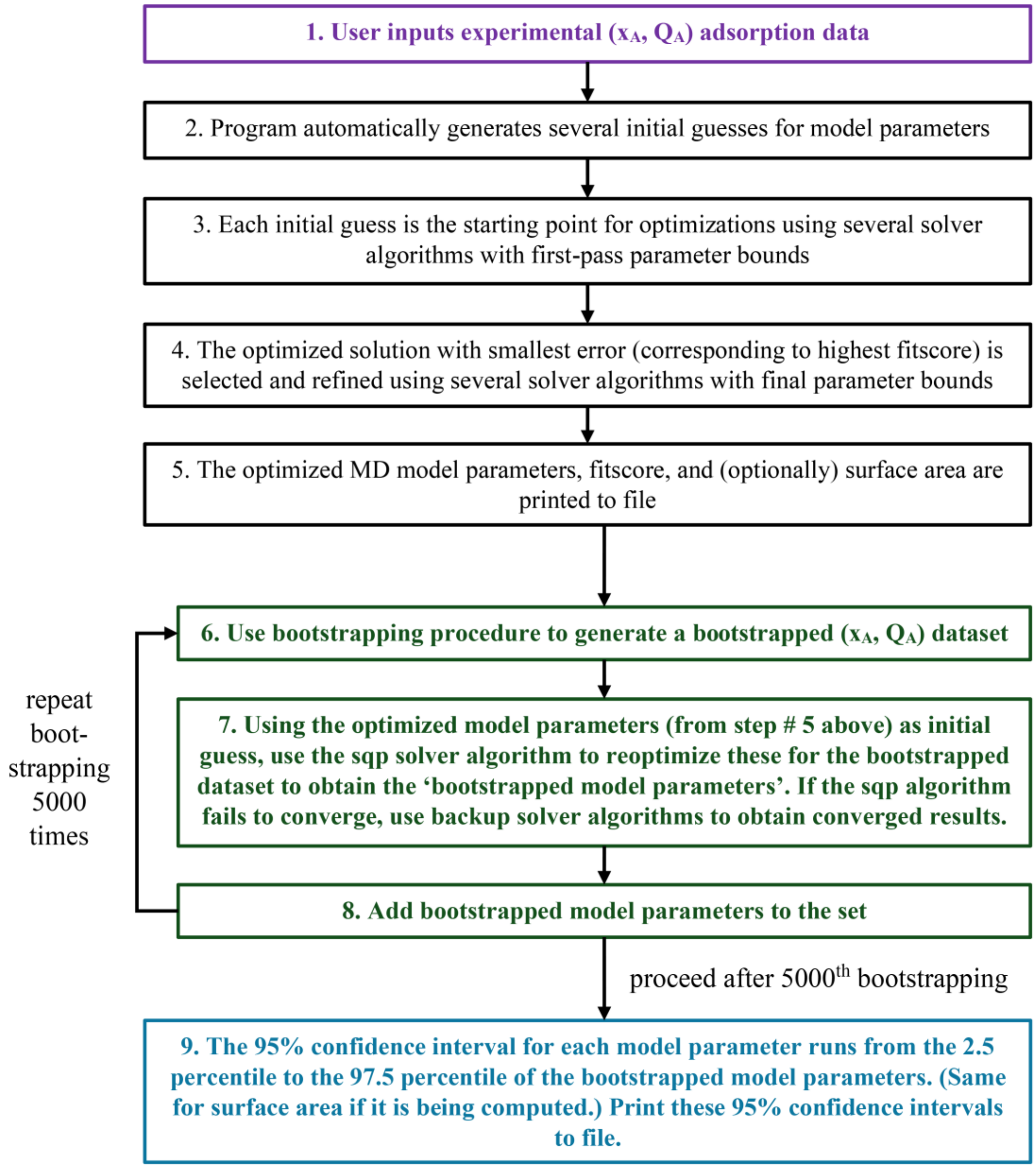

Figure S16: Flow diagram for the program that optimizes MD model parameters and computes 95% confidence intervals. The boxes are colored as follows: reading input files (purple), computing the optimized parameter values (black), bootstrapping (green), 95% confidence intervals (blue).

Each initial guess is the starting point for optimizations using several solver algorithms with first-pass parameter bounds. Since each solver algorithm follows a somewhat different trajectory during the optimization sequence, using multiple solver algorithms guarantees the optimization landscape is more fully explored. This increases the probability that one of the solver algorithms converges to the global minimum starting from one of the initial guesses. In the current code version, the following fmincon solver algorithms are used: 'sqp', 'interior-point', 'active-set', and 'sqp-legacy'. There is nothing magical about these particular algorithms; the objective here is to use several algorithms. Each initial guess is the starting point for optimizations using each of these algorithms.

If one of the solver algorithms is taking too long, it can be disabled by commenting out the corresponding code section. For example, while running the MD model parameter optimization for LAC_adsorbed_on_BS (i.e., alpha-Lactalbumin (LAC) protein adsorbed on the Butyl Sepharose 4FF (BS) resin), the active-set algorithm was running too slow (i.e., not making observable progress for an hour or more) and was commented out in the use_initial_guess_to_optimize_solution.m file. This had the effect of skipping the active-set algorithm when optimizing from the initial guesses. The program ran fine, because at least one of the remaining fmincon solver algorithms (e.g., 'sqp', 'interior-point', 'sqp-legacy') found the solution.

During the first-pass optimizations starting from the initial guesses, the constraint $\alpha \geq 0.25$ is imposed (together with the other constraints listed at the end of this subsection). This constraint prevents the solver from getting 'trapped' near $\alpha = 0$, which occurred in some cases when this constraint was not used. This kind of getting trapped is a symptom of the initial guess being far away from the optimized solution. The program keeps track of the 'best' solution that has the lowest error (i.e., highest fitscore value).

After the first-pass optimizations using the constraint $\alpha \geq 0.25$, a second pass optimization is performed without this constraint. So, the constraint $\alpha \geq 0.25$ is not imposed on the final set of optimized parameter values. The starting point for this second-pass optimization is the 'best' solution that was found from the whole set of first-pass optimizations. For this second-pass optimization, the fmincon function with 'sqp', 'interior-point', 'active-set', and 'sqp-legacy' algorithms was used with central finite differences, OptimalityTolerance = 10^{-12}, ConstraintTolerance=10^{-12}, StepTolerance=10^{-12}, MaxIterations=10000, MaxFunctionEvaluations = 10000. All four algorithms are run from the same starting point (the 'best' solution found from the whole set of first-pass optimizations), and the 'best' second-pass solution is identified as the converged result having the lowest error (i.e., highest fitscore value).

If one of the solvers exits without converging during the first pass or during the second pass, this does not create any problems. The non-converged results are simply ignored, and the program automatically selects the 'best' first-pass and second-pass solutions from among the converged results. An error would be thrown (and the program would exit with the message 'Solver did not converge. Program will terminate.') only in the extremely rare case that none of the algorithms was able to converge to a solution.

During bootstrapping, the optimized parameters from step #5 of the flowchart in Figure S16 are used as the starting point for the model parameter optimization of the bootstrapped data.

In such a way, the bootstrapped data generates a 'perturbed' set of parameter values that slightly differ from the parameter values optimized to the experimental data. During bootstrapping, fmincon is used with a sequence of solver algorithms: 'sqp' (with 10^{-9} convergence tolerances), 'interior-point' (with 10^{-9} convergence tolerances), 'sqp' (with 10^{-8} convergence tolerances), 'interior-point' (with 10^{-8} convergence tolerances), 'active-set' (with 10^{-8} convergence tolerances). As soon as one of the solver algorithms converges, the solution is recorded and the remaining solver algorithms are skipped. Consequently, for the vast majority of bootstrapped data, the bootstrapped parameter values are found via 'sqp' (with 10^{-9} convergence tolerances). Only in rare cases where that algorithm fails to converge does the next algorithm (i.e., 'interior-point' (with 10^{-9} convergence tolerances)) get used.

During bootstrapping, the second, third, fourth, etc. solver algorithms are employed only as backups. For example, the fourth solver algorithm gets used only in the extremely rare situations where the first three algorithms failed to converge. This is different from the initial guess optimizations described at the beginning of this subsection, where all algorithms were used to optimize every initial guess.

When there is no surface area computation, parameter bounds during final (i.e., second-pass) and bootstrapping optimizations simply constrain each model parameter to be non-negative with no upper bounds: $\alpha \geq 0,\ \mathrm{K1} \geq 0,\ \mathrm{K2} \geq 0,\ \mathrm{m} \geq 0,\ \mathrm{N} \geq 0$.

If surface area calculation is included, parameter bounds during final (i.e., second-pass) and bootstrapping optimizations are as follows. K2 is constrained to be between 2/3 and 3/2: $(2/3) \leq \mathrm{K2} \leq 1.5$. Since $\mathrm{K2} = 1.0$ is the bulk liquid interaction value, it is reasonable to constrain K2 so that it does not differ too much from this value. This constraint ensures that K2 does not reach unphysical values. When surface area calculation is included, it is reasonable to assume that the highest adsorbed amount covers at least half of a monolayer, and this corresponds to the constraint $0 \leq \mathrm{N} \leq 2\mathrm{QA\_exp[Ndata]}$. The remaining parameters are simply constrained to be non-negative: $\alpha \geq 0,\ \mathrm{K1} \geq 0,\ \mathrm{m} \geq 0$.

S6.4 Program updates

If any updates to the Matlab code for computing optimized MD model parameters, whole isotherm surface area, or 95% confidence intervals is needed, the updated Matlab codes will be posted to the MATLAB Central File Exchange:

https://www.mathworks.com/matlabcentral/fileexchange/183302-md-model-isotherm-parameter-and-surface-area-program

S6.5 Excel's GRG solver: What if you want to fix one of the parameters or use multiple sitegroups?

If you want to hold one of the parameters fixed at a particular value and not optimize it, then it is recommended to do this in Excel using the Generalized Reduced Gradient (GRG) solver. An example of this is provided in the spreadsheet water_physisorption_on_graphitized_carbon_black_with_isotherm_fitting_02_14_2026.xls where the value of N was held rigid. (Since the surface area of the measured sample was 1 m^2, the value of N was fixed accordingly.)

To use the GRG solver, first click on the cell holding the fitscore value. Then, click on the 'Solver' icon under the 'Data' tab in Excel. Set the objective cell to the cell holding the fitscore

value. Click on the 'Max' radial button to tell the solver to maximize the fitscore value. Under the 'By Changing Variable Cells:' select all of the cells containing the model parameters that you wish to optimize. Check the box 'Make Unconstrained Variables Non-Negative'. Select 'GRG Nonlinear' from the dropdown menu as the solving method. Click on the 'Options' button and set the options to: (a) constraint precision = 10^{-6}, (b) convergence tolerance = 10^{-6}, (c) use central finite differences, (d) use automatic scaling. Click the 'OK' button, and then the 'Solve' button to generate the optimized solution.

Excel's GRG solver was used to obtain the MD model parameters when using more than one sitegroup. An example of this is shown in the 'two_sitegroups' worksheet in the workbook methane_physisorption_MgO_with_isotherm_fitting_02_16_2026.xls

Excel's GRG solver was also used to optimize the parameters of other models including: Sips, Langmuir, and two-site (aka 'dual site') Langmuir. Several spreadsheets in the enclosed zip archive provide examples of this.

References:

(S1) Manz, T.A. "Selective Hydrogenation of Butyronitrile on Promoted Raney(r) Nickel Catalysts," Master's thesis, Major professor: W.N. Delgass, Purdue University, West Lafayette, Indiana, 1998.

(S2) Manz, T. A.; Delgass, W. N.; Gaskell, D. R.; Trumble, K. P.; Liu, Z.; Roberts, D.; Hager, C.; Sengupta, S. K.; Koch, T. A., Porous metals formed by leaching Mn-Ni alloys, submitted to *ACS Omega*, 2026.

(S3) Raney, M. Method of producing finely-divided nickel, *U.S. Patent US1628190,* 1927.

(S4) Dou, S. X.; Zhang, Z. H.; Gao, D.; Zhang, B. W.; Qiang, G. R.; Sun, Z. H. 100 years of Raney Ni catalyst. *Adv. Mater.* **2026**, *38*, e21850, doi: 10.1002/adma.202521850.

(S5) Fouilloux, P. The nature of Raney nickel, its adsorbed hydrogen and its catalytic activity for hydrogenation reactions (review). *Appl. Catal.* **1983**, *8*, 1-42, doi:10.1016/0166-9834(83)80051-7.

(S6) Schmidt, S.R. The Raney(r) catalyst legacy in hydrogenation. In *Hydrogenation*, Jackson, S.D., Ed.; De Gruyter: Boston, 2018; pp. 19-42.

(S7) Raney, M. Method of preparing catalytic material, *U.S. Patent US1563587A,* 1925.

(S8) Raney, M. Catalysts from alloys: nickel catalysts. *Ind. Eng. Chem. Res.* **1940**, *32*, 1199-1203, doi:10.1021/ie50369a030.

(S9) Klein, J.C.; Hercules, D.M. Surface analysis of Raney-nickel alloys. *Anal. Chem.* **1981**, *53*, 754-758, doi:10.1021/ac00229a003.

(S10) Choudhary, V.R.; Chaudhari, S.K.; Gokarn, A.N. A kinetic model for leaching process in preparation of Raney nickel catalyst. *Ind. Eng. Chem. Res.* **1989**, *28*, 33-37, doi:10.1021/ie00085a007.

(S11) Bakker, M.L.; Young, D.J.; Wainwright, M.S. Selective leaching of $NiAl_3$ and Ni_2Al_3 intermetallics to form Raney nickels. *J. Mater. Sci.* **1988**, *23*, 3921-3926, doi:10.1007/BF01106814.

(S12) Fasman, A.B.; Nurumbetov, K.A.; Almashev, B.K.; Nalibayev, T.N. Diffractometric study of structure of Raney-nickel containing added zirconium, tantalum and manganese. *React. Kinet. Catal. Lett.* **1975**, *2*, 89-96, doi:10.1007/BF02060957.

(S13) Bard, A.J.; Parsons, R.; Jordan, J. *Standard Potentials in Aqueous Solution*; International Union of Pure and Applied Chemistry, Marcell Dekkar Inc.: New York, 1985.

(S14) Huff, J.R.; Jasinski, R.J.; Parthasarathy, R. Adsorption of gases on Raney nickel. *Ind. Eng. Chem., Process Des. Dev.* **1964**, *3*, 159-164, doi:10.1021/i260010a011.

(S15) Schmidt, S.R. Surfaces of Raney(r) Catalysts. In *Catalysis of Organic Reactions*, Scaros, M.G., Prunier, M.L., Eds.; Chemical Industries; Marcel Dekker: New York, 1995; pp. 45-59.

(S16) Thomas-Pryor, S.N.; Manz, T.A.; Liu, Z.; Koch, T.A.; Sengupta, S.K.; Delgass, W.N. Selective hydrogenation of butyronitrile over promoted Raney(r) nickel catalysts. In *Catalysis of Organic Reactions*, Herkes, F., Ed.; Mercel Dekker: New York, 1998; pp. 195-206.

(S17) Greenfield, H. Catalytic hydrogenation of butyronitrile. *Ind. Eng. Chem. Res.* **1967**, *6*, 142-144, doi:10.1021/i360022a014.

(S18) Lieber, E.; Morritz, F.L. The uses of Raney nickel. *Adv. Catal.* **1953**, *5*, 417-455, doi:10.1016/S0360-0564(08)60647-1.

(S19) Csuros, Z.; Petro, J. Possibilities of increasing the activity of catalysts of Raney-nickel type. *Period. Polytech. Chem. Eng.* **1959**, *3*, 123-142.

(S20) Pickett, G. Modification of the Brunauer-Emmett-Teller adsorption theory. *J. Am. Chem. Soc.* **1945**, *67*, 1958-1962, doi:10.1021/ja01227a027.

(S21) Barrett, E. P.; Joyner, L. G.; Halenda, P. P. The determination of pore volume and area distributions in porous substances .1. Computations from nitrogen isotherms. *J. Am. Chem. Soc.* **1951**, *73*, 373-380, doi:10.1021/ja01145a126.

(S22) Lee, Y. H.; Lee, S. W.; Kim, H. J.; Kim, Y. T.; Lin, K. Y. A.; Lee, J. C. Hydrogenation of adiponitrile to hexamethylenediamine over Raney Ni and Co catalysts. *Appl. Sci.-Basel* **2020**, *10*, 7506, doi:10.3390/app10217506.

(S23) Allgeier, A. M.; Sengupta, S. K. Nitrile hydrogenation. In *Hydrogenation*, Jackson, S. D. Ed.; De Gruyter: Boston, 2018; pp 107-154.
